# Metal-Free Catalytic Synthesis of Tetrasubstituted Furans from *α*-Hydroxy Ketones and Cyano Compounds

**DOI:** 10.3390/molecules30081832

**Published:** 2025-04-19

**Authors:** Yu Zeng, Shi-Hang Yang, Ji-Lin Guo, Yun Li, Ting Lin, Zhao-Yang Wang

**Affiliations:** School of Chemistry, South China Normal University, Guangzhou Key Laboratory of Analytical Chemistry for Biomedicine, GDMPA Key Laboratory for Process Control and Quality Evaluation of Chiral Pharmaceuticals, Key Laboratory of Theoretical Chemistry of Environment, Ministry of Education, Guangzhou 510006, China; 2023022534@m.scnu.edu.cn (Y.Z.); 2024022743@m.scnu.edu.cn (S.-H.Y.); 2024022665@m.scnu.edu.cn (J.-L.G.); 2024022879@m.scnu.edu.cn (Y.L.); 20232421068@m.scnu.edu.cn (T.L.)

**Keywords:** *α*-hydroxy ketones, cyano compounds, tetrasubstituted furans, green synthesis, metal-free catalysis

## Abstract

A novel method for the efficient and straightforward synthesis of tetrasubstituted furans is presented, employing a base-catalyzed reaction of *α*-hydroxy ketones and cyano compounds. The reaction proceeds under relatively mild conditions, utilizes readily available starting materials, and exhibits good functional group tolerance and high yields. Notably, this reaction obviates the need for expensive metal catalysts and introduces crucial functional groups such as amino and cyano moieties. Furthermore, it avoids the prerequisite functionalization of substrates, thereby enhancing atomic economy.

## 1. Introduction

**Figure 1:**
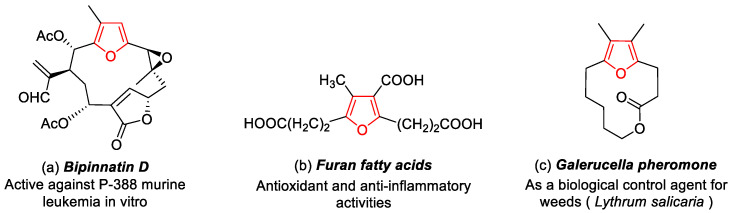
Some biologically active polysubstituted furan compounds.

Polysubstituted furan rings are prevalent in numerous natural products [1] and pharmaceutical molecules [2]; some key examples of furan-containing structures are shown in Figure 1 [3,4,5]. Furthermore, the furan ring motif serves as a valuable structural scaffold, playing a crucial role in diverse fields such as polymeric materials [6], organic functional materials [7], and synthetic chemistry [8]. Consequently, polysubstituted furans, particularly tetrasubstituted furan compounds, have garnered increasing attention from the scientific community [9], leading to the development of dedicated methodologies for the synthesis of functionalized tetrasubstituted furan compounds [10,11].

**Scheme 1:**
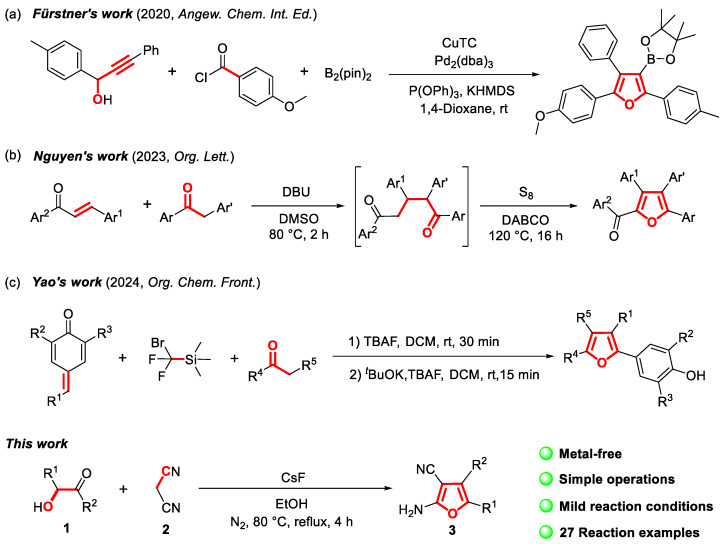
Strategy for obtaining tetrasubstituted furan compounds [18,19,20].

Generally, the furan scaffold can be constructed via metal catalysis [12], non-metal catalysis [13,14], photocatalysis [15], and electrocatalysis [16]. Among these methods, the cyclization of carbonyl compounds and enynols using transition metal catalysis to afford the desired furan products is relatively common [17]. For instance, Fürstner’s group [18] recently reported the synthesis of tetrasubstituted furans (Scheme 1a) using propargylic alcohols, bis(pinacolato)diboron (B_2_(pin)_2_), and acyl chlorides as starting materials. The reaction, catalyzed by a palladium/copper metal catalytic system with potassium bis(trimethylsilyl)amide (KHMDS) as the base and P(OPh)_3_ as the ligand, proceeds through a series of steps including the borylation of propargylic alcohol and acyl chloride, regioselective acylation, cyclization and dehydration, and Suzuki coupling.

It is evident that the development of these synthetic reactions without the involvement of metal catalysts would be more economical and environmentally benign. Consequently, Nguyen’s group [19] reported the efforts in this area when focusing on the synthesis of tetrasubstituted furans. Their approach involves the reaction between chalcones and desoxybenzoins in the presence of S_8_/1,4-diazabicyclo [2.2.2] octane (DABCO)/DMSO. The reaction is efficiently catalyzed by 1,8-diazabicyclo[5.4.0]undec-7-ene (DBU), initially yielding 1,2,3,5-tetraarylpentan-1-ones. Then, these intermediates undergo further oxidative cyclization, ultimately leading to the formation of tetrasubstituted furans (Scheme 1b).

Despite this progress in the research without the use of expensive transition metal catalysts, the challenge of simultaneously circumventing the higher temperatures and the prolonged reaction time remains a significant area for investigation. In this context, Yao’s group [20] recently reported a three-component, one-pot reaction involving difluorobromomethyltrimethylsilane (TMSCF_2_Br), *para*-quinone methides (*p*-QMs), and carbonyl compounds. This reaction, employing potassium *tert*-butoxide as a base and tetrabutylammonium fluoride (TBAF) as an additive, was successfully conducted at room temperature, with a total reaction time of less than 1 h (Scheme 1c). The reaction sequence undergoes a serial process, including tandem desilylation/cyclopropanation mediated by TBAF, intermolecular S_N_2 reaction, reverse 1,6-fluoro-addition, keto-enol tautomerization, intramolecular nucleophilic vinyl substitution (SNV) reaction, and aromatization.

Moreover, the synthesis of tetrasubstituted furans under mild conditions has been reported by only using an organic base catalyst, without the need for additional additives. For instance, Gewald’s group [21] synthesized the tetrasubstituted furans in a 51–67% yield by using triethylamine as the base and DMF as the solvent, reacting at room temperature for 1–4 h. Similarly, Hsieh’s group [22] achieved the synthesis of tetrasubstituted furans in a 40–67% yield after a 16-h reaction with diethylamine as the base. Recently, Farouk’s group [23] also synthesized the tetrasubstituted furans by using diethylamine as the base, reacting at room temperature for 24 h. It can be seen that these reactions are often characterized by suboptimal yields or an extended reaction time. Consequently, further developing the mild conditions for the synthesis of the tetrasubstituted furans, with a focus on improving the yield and reducing the reaction time, is of paramount importance.

In a word, much significant progress has been made in the green synthesis of tetrasubstituted furans by employing 1,3-dicarbonyl compounds [24], alkenynes [25,26], and ketenes [27] as substrates. Despite these advancements, the development of more flexible and straightforward methodologies for the synthesis of diverse polysubstituted furans remains an active area of investigation [28,29,30]. Especially, it remains important to continuously explore the environmentally benign synthetic routes featuring milder reaction conditions, such as a lower temperature and shorter reaction time, and to avoid the use of expensive transition metal catalysts and harsh oxidants [31].

On the other hand, *α*-hydroxy ketone compounds are important, not only because the structural motif of *α*-hydroxy ketone is found in natural products and pharmaceuticals [32]. The presence of both carbonyl and hydroxyl groups in *α*-hydroxy ketones endows them with unique reactivity [33]. For instance, they can act as nucleophiles under the catalysis of basic or Lewis acid to form C–C bonds with electrophiles [34]. Furthermore, they can serve as 1,3-dipoles in cycloaddition reactions, providing an efficient and convenient route to highly functionalized oxygen-containing heterocycles [35].

Despite the utility of α-hydroxyketones as starting materials, the efficient synthesis of tetrasubstituted furans remains relatively unexplored. Only Jiang’s group [36] inadvertently reported the synthesis of tetrasubstituted furans, showing one example with a moderate yield of 68% during the condition optimization for other synthesis, by employing cesium fluoride as a base and methanol as a solvent under a nitrogen atmosphere at 110 °C for 24 h. However, this reaction suffers from a longer reaction time and a relatively higher temperature. Simply, as far as we know, the methodology has not been extensively investigated within the existing literature.

Herein, we hypothesized that the sequential reaction of cyano compounds (using malononitrile as an example in Scheme 1, ***this work***) with the carbonyl and hydroxyl groups of benzoin, under an appropriate base catalysis, would enable the construction of the target tetrasubstituted furan products. Gratifyingly, the reaction can proceed as anticipated. This transformation is achieved by employing cesium fluoride as the base under reflux in ethanol at a relatively lower temperature. Thus, this methodology not only facilitates a green synthesis, incorporating functional groups such as amino and cyano moieties, but also circumvents the need for a pre-functionalization of the substrate, thereby enhancing the atomic economy and aligning with the tenets of green chemistry and sustainable development.

## 2. Results and Discussion

### 2.1. Optimization of Reaction Conditions

Using benzoin **1a** and malononitrile **2a** as the model substrates, we conducted a reaction condition optimization for the synthesis of product **3a**. We investigated the influence of different factors, such as the base, solvent, temperature, reaction time, and reactant molar ratio on the isolated yield of **3a**. The results are presented in Table 1.

Initially, the impact of varying atmospheres on the yield of product **3a** was investigated by employing cesium fluoride as the base and ethanol as the solvent. The molar ratio of reactants was maintained at **1a**:**2a**:Base = 1:2:3, with a reaction time of 3 h. The experimental results reveal that the yield of product **3a** is only 47% under an air atmosphere (Entry 1). Conversely, the yield can be significantly increased to 90% under an N_2_ atmosphere (Entry 2). This observation suggests that α-hydroxyketone **1a** is susceptible to oxidation in the presence of air, which adversely affects the reaction progress and reduces the yield. The use of an inert nitrogen atmosphere effectively prevents the oxidation of the α-hydroxyketone reactant, thereby facilitating a more efficient reaction and enhancing the yield. These findings underscore the critical importance of an inert atmosphere for this reaction and provide essential insights for subsequent optimization of the reaction conditions.

In addition, for the experimental atmosphere and pressure environment, as the same conditions in Entry 2, a reaction was also conducted in a Schlenk pressure tube as the sealed reaction vessel, giving product **3a** a yield of 72% (Entry 3). There is significantly lower efficiency compared to the reaction conducted in a flask. This discrepancy may be attributed to the generally inferior sealing of the flask relative to the sealed tube. As the reaction progresses, the pressure within the sealed tube is increased. At a certain inner temperature and high pressure, the water molecules generated in the reaction may be vaporized, inhibiting the forward reaction. Therefore, a lower pressure may be more conducive to the reaction.

Subsequently, we investigated the influence of various bases (Entries 4–17). The experimental outcomes reveal that employing bases such as cesium carbonate (Cs_2_CO_3_), sodium acetate (AcONa), potassium *tert*-butoxide (*^t^*BuOK), and 2-methylimidazole (2-MI) [37] only results in a maximum yield of 61% (Entry 16). This is significantly lower than the yield of **3a** (90%) when cesium fluoride is utilized as the base (Entry 2).

Next, we investigated the impact of common solvents, including methanol (MeOH), *N*,*N*-dimethylformamide (DMF), and dimethyl sulfoxide (DMSO), on reaction outcomes (Entries 18–28; the majority of reactions were conducted at the solvent’s boiling point; for DMF and DMSO, the temperature is maintained at 110 °C for the comparative analysis with solvents like toluene). Notably, DMSO can exhibit superior performance as a solvent (Entry 22, 89%), albeit with a slightly reduced yield compared to anhydrous ethanol (Entry 2, 90%).

Of course, we also investigated the impact of the reaction temperature and time on the yield. Obviously, using EtOH as a solvent, lowering the temperature produces a lower yield (Entries 29–30 vs. Entry 2). When the reaction time is increased from 3 h to 4 h, the yield is increased from 90% (Entry 2) to 94% (Entry 31). However, a further increase of 1 h in reaction time results in a slight decrease in yield (Entry 32, 90%). It is evident that both the shorter and longer reaction time may lead to the lower yield of **3a**. Therefore, 4 h is determined to be the optimal reaction time for this reaction (Entry 31, 94%).

Finally, based on the aforementioned findings, we investigated the impact of reactant stoichiometry on the reaction (Entries 33–36 vs. Entry 31). The optimal reaction outcome can be observed with a molar ratio of **1a**:**2a**:Base at 1:2:3 (Entry 31, 94%).

Thus, the optimized reaction conditions were established as follows: using benzoin **1a** (0.3 mmol) and malononitrile **2a** (0.6 mmol) as substrates, cesium fluoride (0.9 mmol) as the base, anhydrous ethanol (5 mL) as the solvent, under an N_2_ atmosphere, and refluxing at 80 °C for 4 h. Under these conditions, the yield can reach a maximum of 94% (Entry 31).

### 2.2. Scope of Substrates

Under the optimized reaction conditions, we explored the substrate scope to further assess the generality of this reaction.

We investigated the impact of *α*-hydroxy ketones with various substituents on the reaction (Table 2), including common electron-donating groups (e.g., methyl, ethyl, isopropyl, *tert*-butyl), halogens (e.g., fluorine, chlorine, bromine), and electron-withdrawing groups (e.g., trifluoromethyl) attached to the benzene ring.

The experimental results demonstrate that most of the products can be obtained in satisfactory yields when using different *α*-hydroxy ketones **1** to react with malononitrile **2a**. Of course, the various substituents in substrates **1** show different effects on the reaction yield, which may be related to the mechanism in many cases.

For example, the reaction may proceed with high yields when the aryl group (Ar) has electron-donating substituents at the para position of the benzene ring, such as methyl, ethyl, isopropyl, or *tert*-butyl groups (**3b**–**3e**, 73–81%). The reaction also proceeds smoothly when the para substituent is a halogen atom, such as fluorine, chlorine, or bromine (**3f**–**3h**, 81–87%), and these yields are slightly higher compared to electron-donating groups.

The target product **3i** can be obtained in a higher yield (95%) when the para substituent is the strongly electron-withdrawing trifluoromethyl group. Especially, the target product **3u** is obtained in the highest yield (97%) when R is a phenyl group. Therefore, in general, the presence of electron-withdrawing groups can result in higher yields of the target products compared to electron-donating groups. This may be due to the enhanced reactivity of the carbonyl carbon caused by the presence of electron-withdrawing groups, which facilitates the attack by the nucleophile malononitrile **2a**.

Furthermore, this reaction exhibits good tolerance for *α*-hydroxy ketones **1** with meta-substituted R groups, affording the desired products in good yields (**3j**–**3n**, 76–84%). The experimental outcomes consistently demonstrate that the yields of meta-substituted products are inferior to those of para-substituted products. This observation can be attributed to a combination of electronic, steric, and resonance effects.

Specifically, when the substituent is a methyl group, the yield for the meta-substituted product is lower than that of the para-substituted product **3b** (81%) vs. **3j** (77%), likely due to electronic and steric influences. The electron-donating effect of the methyl group at the para position enhances the electron density on the oxygen anion in the intermediate (the intermediate **II**), thereby increasing its reactivity and facilitating the reaction. Conversely, when the methyl group is at the meta position, the electron-donating effect may be negligible, while steric hindrance becomes a significant factor. Obviously, the latter is not conducive to reaction.

Similarly, the yields of meta-substituted products are generally lower than those of para-substituted products when the substituents are halogens. For instance, when the substituent is fluorine, **3f** (87%) vs. **3k** (84%); when the substituent is chlorine, **3g** (85%) vs. **3l** (82%); and when the substituent is bromine, **3h** (81%) vs. **3m** (76%). This phenomenon may be attributed to the electronic effect, steric hindrance, and resonance effect. No matter whether the halogen atom existed in the para- or meta-position, there is a negative impact on the activity of the oxygen anion due to the electron-withdrawing effect. However, when the halogen atom existed in the para-position, due to the resonance effect, the electron density of the oxygen anion in the intermediate may increase, enhancing its activity and facilitating the reaction. For the halogen atom in the meta-position, there is no resonance effect. Furthermore, the steric hindrance effect is significant in meta-substitution, thus resulting in generally lower yields compared to para-substitution.

When the substituent is the strongly electron-withdrawing trifluoromethyl group, the yield of the meta-substitution is also significantly lower than that of the para-substitution, **3i** (95%) vs. **3n** (80%). This may be due to the fact that it not only has a significant steric hindrance effect, which hinders the reaction process, but also the strong electron-withdrawing effect in the meta-position significantly reduces the electron density of the oxygen anion, leading to a substantial decrease in reaction activity.

We also investigated the applicability of the protocol to *α*-hydroxy ketone substrates bearing two substituents. Gratifyingly, the desired products can be obtained in moderate yields when methyl, fluoro, or chloro groups are present at the C-3 and C-5 positions of the phenyl ring (**3o**–**3q**, 65–71%). However, the yields of the disubstituted products are generally lower than those of the monosubstituted counterparts, possibly due to the increased steric hindrance. For instance, the yield of the 3,5-disubstituted product is the lowest of all products with the same group, for methyl, **3o** (65%) vs. **3j** (77%) vs. **3b** (81%); for fluorine, **3p** (71%) vs. **3k** (84%) vs. **3f** (87%); and for chlorine, **3q** (68%) vs. **3l** (82%) vs. **3g** (85%). Another reason for the relatively low yield may be involved in the product stability. Obviously, compared to the structures of the monosubstituted counterparts **3g** and **3s** (their molecular structural figures can be seen in Table 2), the presence of two R groups significantly increases steric hindrance, potentially leading to a deviation of the furan ring from its planar conformation. This distortion may bring out greater disruption for the intramolecular conjugation in the product. Notably, the target product **3t** (67%) also can be obtained in a moderate yield when the aryl group is the furyl group.

In addition, we investigated the substrate scope of **2** under the above-mentioned conditions, but the reaction of reactant **1** with ethyl cyanoacetate (**2b**) instead of malononitrile (**2a**) failed to yield the desired product. Therefore, by maintaining similar conditions, we employed piperidine as the base and toluene as the solvent, but only refluxing the reaction at 80 °C for 8 h to keep the same temperature as that of the reaction of **2a**. It can be observed that the predicted target products **3v**–**3aa** are formed.

Though there is a similar yield change when comparing to the reaction of **2a**, overall, the reactions of the monocyano-substituted **2b** exhibit inferior performance compared to those employing malononitrile **2a**, obviously resulting in the lower isolated yields. According to the proposed mechanism (see the discussion in the reaction mechanism section for details), we hypothesize that the reduced opportunity for the oxygen anion to attack the cyano carbon in the monocyano substrate **2b** affects the reaction yield.

In a word, we extensively investigated the substrate scope, resulting in the synthesis of a total of 27 target compounds (including 18 novel compounds). And their structures are well characterized with NMR, HR-MS, and single-crystal X-ray diffraction analysis, especially for **3g**, **3s**, and **3v** (their structural figures can be seen in Table 2 or the Supplementary Materials) [38].

### 2.3. Mechanism Investigation

**Scheme 2:**
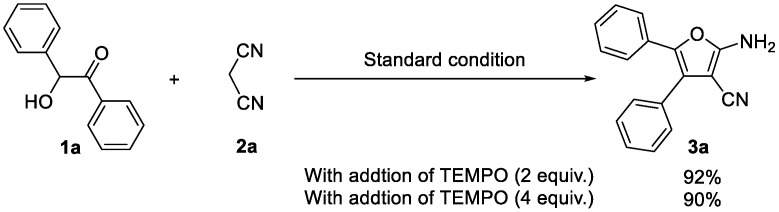
Free radical control experiment.

To further elucidate the reaction mechanism, a control experiment was conducted. Under the standard reaction conditions using **2a** as a reactant, the radical inhibitor 2,2,6,6-tetramethylpiperidine-1-oxyl (TEMPO) [39] was added. It can be observed that the reaction is not significantly inhibited upon the addition of 2 equiv. or 4 equiv. of TEMPO (Scheme 2). And the desired product **3a** is still obtained in a high yield, indicating that the reaction does not involve a radical process.

**Figure 2:**
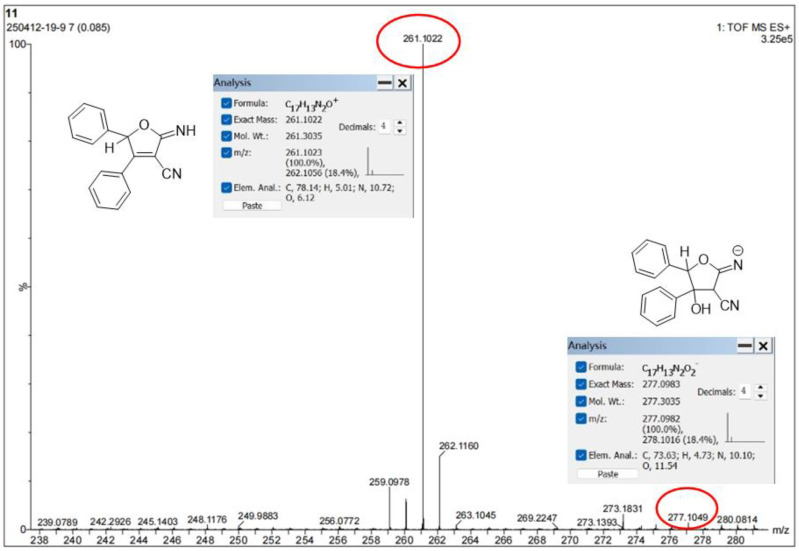
HRMS test for the relevant intermediates.

**Scheme 3:**
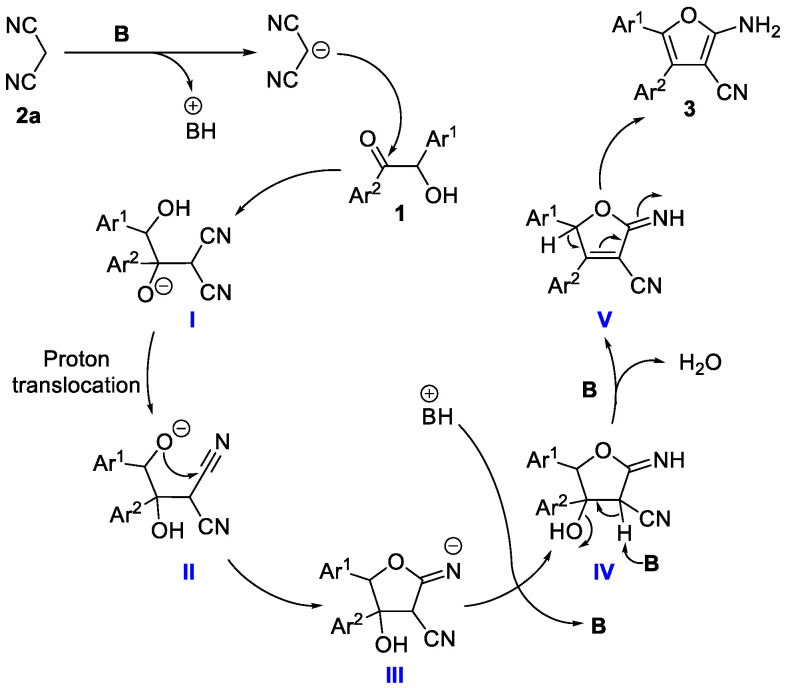
Possible reaction mechanism.

Based on this control experiment, the HRMS test for the reaction solution (the relevant mass spectrometry data involving the intermediates are presented in Figure 2), and prior literature [36], we proposed a plausible reaction mechanism, as depicted in Scheme 3 using the reaction of **2a** as the example.

Initially, malononitrile **2a**, under the influence of cesium fluoride (Base, denoted as **B**), undergoes deprotonation to generate a carbanion [40]. This carbanion subsequently attacks the carbonyl carbon of benzoin **1**, affording intermediate **I**. Then, intermediate **I** undergoes a proton shift, yielding intermediate **II**. The oxygen anion of **II** intramolecularly attacks the adjacent cyano carbon [41], resulting in the formation of cyclic intermediate **III**. Intermediate **III** undergoes a proton transfer to form intermediate **IV**, which further undergoes intramolecular dehydration [42], giving the imine intermediate **V**. Finally, intermediate **V** undergoes keto-enol tautomerization [43], yielding the final product **3**.

The formation of intermediate **II**, as illustrated by the mechanism in Scheme 3, is the crucial step in this reaction, ensuring the attack of the oxygen anion on the cyano carbon. Consequently, conducting the reaction under a nitrogen atmosphere is essential to preserving the hydroxyl group in benzoin, preventing its oxidation to benzil [44,45].

On the other hand, it can be seen from intermediate **II** that, when malononitrile **2a** is employed, the oxygen anion has a higher probability of attacking the cyano group, thereby yielding the desired product. Conversely, when using monocyano compound **2b** as a substrate, the attack probability is obviously reduced, resulting in a lower isolated yield of the target product **3**.

### 2.4. Gram Scale Reaction and Derivatization Applications

**Scheme 4:**
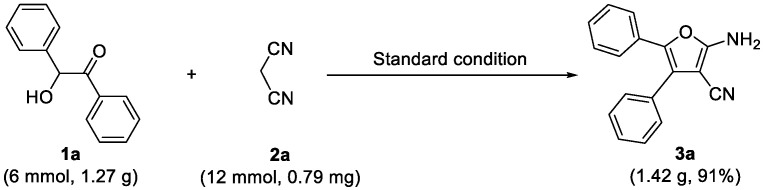
The Gram-scale reaction for the synthesis of **3a**.

Tetrasubstituted furans are highly valuable in organic synthesis and medicinal chemistry [46,47]. Therefore, to further investigate the method’s applicability in practical large-scale preparation, we carried the reaction by scaling up 20-fold from the original usage (Scheme 4). It can be seen that, although the reaction yield is decreased slightly, the target product **3a** is still obtained in a high yield of 91%. This preliminarily demonstrates the potential value of our experimental method in a practical application.

**Scheme 5:**
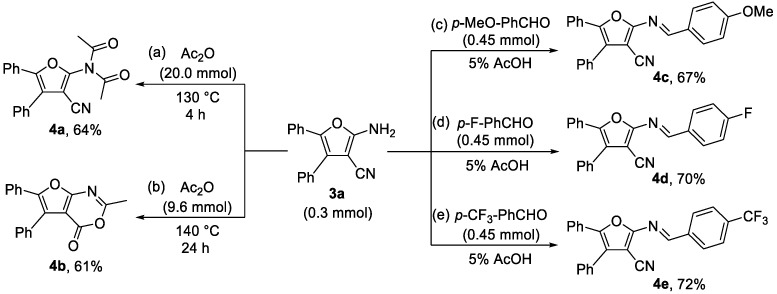
Derivatization reactions of **3a**.

To further investigate the potential application of the synthesized compounds, we conducted the functional group derivatization experiments by using product **3a** as the starting material (Scheme 5).

Initially, the amino group in **3a** is reacted with acetic anhydride [48], affording the target amide compound **4a** in a moderate yield. Furthermore, by carefully controlling the amount of acetic anhydride, reaction temperature, and reaction time [49], the target furan-fused oxazine scaffold compound **4b** also can be obtained in a moderate yield.

At the same time, an aldimine condensation reaction by using the amino group in compound **3a** with *p*-methoxybenzaldehyde, *p*-fluorobenzaldehyde, and *p*-trifluoro- methylbenzaldehyde, respectively, can also be smoothly performed, yielding the corresponding furan Schiff base derivatives **4c**, **4d**, and **4e** in moderate yields (Scheme 5).

## 3. Materials and Methods

### 3.1. General Information

The ^1^H NMR spectra were acquired on a Bruker DRX-600 spectrometer at 600.0 MHz and the ^13^C NMR spectra were acquired at 150.0 MHz, using deuterated chloroform (CDCl_3_) or deuterated dimethyl sulfoxide (DMSO-*d*_6_) as solvents and TMS as an internal standard. High-resolution mass spectra were obtained using a MAT 95XP mass spectrometer (Thermo Fisher Scientific, Waltham, MA, USA) and a Waters G2-XS Qtof mass spectrometer (Waters, Milford, MA, USA). Single-crystal X-ray diffraction data were collected on an Agilent Gemini E diffractometer (Agilent Technologies, Santa Clara, CA, USA) with Mo Kα radiation. Reactions were monitored by thin-layer chromatography (TLC) and visualized under UV light at 254 nm. Plotting with ChemDraw 19.0.0.22. MestReNova 14.0.0-23239 was used for spectral data analysis.

All reagents and solvents were obtained commercially and used without further purification. *α*-Hydroxy ketones **1a**–**1u** were synthesized from various aldehydes according to the procedure reported in the literature [50].

### 3.2. Experimental Procedure for α-Hydroxy Ketones ***1a***–***1u***

**Scheme 6:**
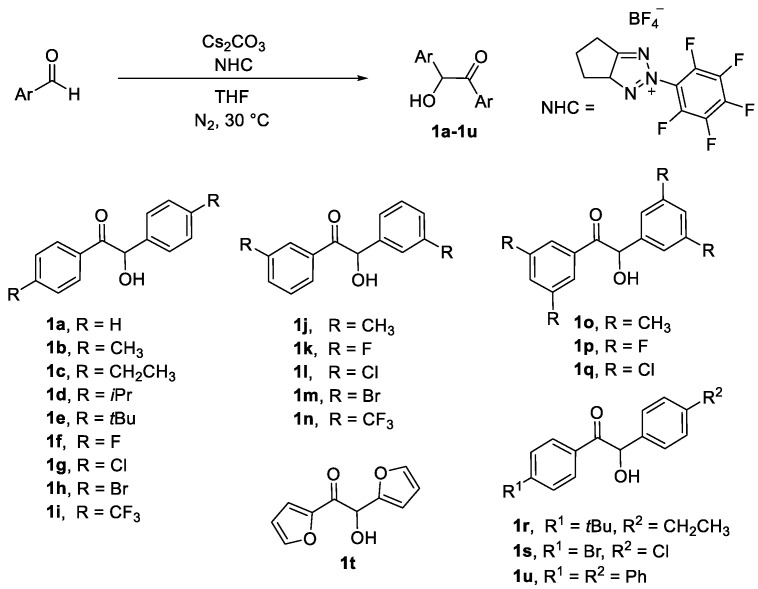
Synthesis of compounds **1a**–**1u**.

According to Scheme 6 and as the reported method [50], the mixture of aryl aldehyde (2 mmol), NHC (0.5% mmol), Cs_2_CO_3_ (0.5% mmol), and THF (100 μL) was stirred under a N_2_ atmosphere for 0.5–12 h as monitored by thin-layer chromatography (TLC). The reaction was quenched when the mixture became a white slurry. The sample was diluted with CH_2_Cl_2_ (10 mL) after quenching with the saturated NH_4_Cl and washed with the saturated NaCl (5 mL), followed by extraction with CH_2_Cl_2_ (3 × 5 mL). The organic phase was then dried over anhydrous Na_2_SO_4_.

Finally, after filtration, the solvent was evaporated under reduced pressure. The crude product was purified by silica gel column chromatography to afford the target compounds **1a**–**1u**.

### 3.3. Experimental Procedure for Compounds ***3a***–***3u***

**Scheme 7:**
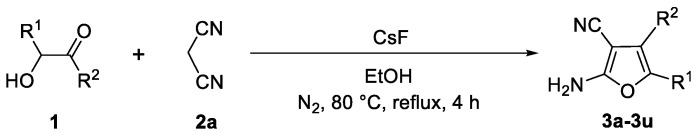
Synthesis of compounds **3a**–**3u**.

According to Scheme 7, the mixture of *α*-hydroxy ketones **1** (0.3 mmol), malononitrile **2a** (0.6 mmol), cesium fluoride (0.9 mmol), and EtOH (5 mL) was stirred under e protection by N_2_ at 80 °C for 4 h. After cooling to room temperature, the sample was diluted with ethyl acetate (EA, 10 mL), washed with the saturated NaCl solution (5 mL), and extracted with EA (3 × 5 mL).

Then, the organic layer was dried with anhydrous Na_2_SO_4_. Finally, after filtration, the solvent was evaporated under reduced pressure. The crude product was purified by silica gel column chromatography to obtain target products **3a**–**3u**.

### 3.4. Experimental Procedure for Compounds ***3v***–***3aa***

**Scheme 8:**
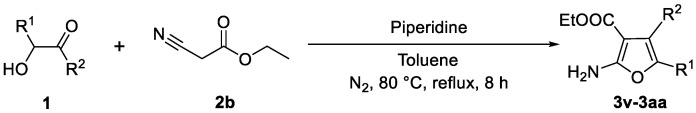
Synthesis of compounds **3v**–**3aa**.

According to Scheme 8, the mixture of *α*-hydroxy ketones **1** (0.3 mmol), ethyl cyanoacetate **2b** (0.6 mmol), piperidine (0.9 mmol), and toluene (3 mL) was stirred under protection by nitrogen at 80 °C for 8 h. After cooling to room temperature, the sample was diluted with EA (10 mL), washed with the saturated NaCl solution (5 mL), and extracted with EA (3 × 5 mL).

Then, the organic layer was dried with anhydrous Na_2_SO_4_. Finally, after filtration, the solvent was evaporated under reduced pressure. The crude product was purified by silica gel column chromatography to obtain target products **3v**–**3aa**.

### 3.5. Experimental Procedure for Compounds ***4a*** and ***4b***

**Scheme 9:**
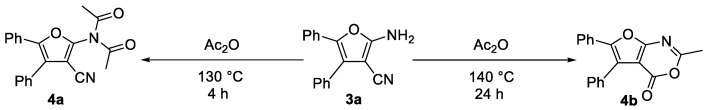
Synthesis of compounds **4a** and **4b**.

According to Scheme 9 and the reported method [48], the mixture of furan compound **3a** (0.3 mmol, 78 mg) and Ac_2_O (2 mL) was refluxed for 4 h at 130 °C under an air atmosphere. After cooling to room temperature, a small amount of methanol was added. After a rotary evaporation, the residue was diluted with EA (10 mL), washed with the saturated NaCl solution (5 mL), and extracted with EA (3 × 5 mL). Then, the organic layer was dried with anhydrous Na_2_SO_4_. Finally, after filtration, the solvent was evaporated under reduced pressure. The crude product was purified by silica gel column chromatography to obtain compound **4a**.

As in the reported method [49], the reaction conditions for compound **4b** were established as follows. The mixture of furan compound **3a** (0.3 mmol, 78 mg) and Ac_2_O (0.9 mL) was refluxed for 24 h at 140 °C under an air atmosphere. All other procedures were identical to those described for compound **4a**.

### 3.6. Experimental Procedure for Compounds ***4c***–***4e***

**Scheme 10:**
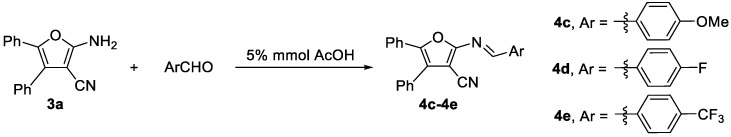
Synthesis of compounds **4c**–**4e**.

According to Scheme 10, after compound **3a** (0.3 mmol, 78 mg), *p*-methoxy- benzaldehyde (0.45 mmol), 5% mmol acetic acid, and 5 mL absolute ethanol were added to the 50 mL round bottom flask, and the mixture was refluxed at 80 °C under an N_2_ atmosphere for 6 h. After the reaction was completed, the solvent was removed by rotary evaporation, and the residual solution was extracted with CH_2_Cl_2_ (3 × 15 mL). Then, the organic layer was dried with anhydrous Na_2_SO_4_. Finally, after filtration, the solvent was evaporated under reduced pressure. The crude product was purified by silica gel column chromatography to obtain the target compounds **4c**.

The reaction method of *p*-fluorobenzaldehyde and *p*-trifluoromethylbenzaldehyde is the same as that of *p*-methoxybenzaldehyde, giving the target compounds **4d** and **4e**.

### 3.7. Characterization Data for All Products ***3a***–***3aa*** and ***4a***–***4e***

(1) *2-Amino-4,5-diphenylfuran-3-carbonitrile* (**3a**): White solid (72.2 mg, yield 93%), m.p. 205.2–205.9 °C (204.0–206.0 °C [51]); ^1^H NMR (600 MHz, DMSO-*d*_6_), *δ*, ppm: 7.72 (*s*, 2H, NH_2_), 7.48–7.44 (*m*, 2H, ArH), 7.43–7.40 (*m*, 1H, ArH), 7.39–7.37 (*m*, 2H, ArH), 7.27–7.22 (*m*, 4H, ArH), 7.19–7.16 (*m*, 1H, ArH).
(2) *2-Amino-4,5-di-p-tolylfuran-3-carbonitrile* (**3b**): White solid (71.7 mg, yield 81%), m.p. 214.8–216.6 °C (216.0 °C [52]); ^1^H NMR (600 MHz, CDCl_3_), *δ*, ppm: 7.33 (*d*, 2H, *J* = 7.8 Hz, ArH), 7.27 (*d*, 2H, *J* = 7.8 Hz, ArH), 7.21 (*d*, 2H, *J* = 7.8 Hz, ArH), 7.06 (*d*, 2H, *J* = 7.8 Hz, ArH), 4.99 (*s*, 2H, NH_2_), 2.40 (*s*, 3H, CH_3_), 2.32 (*s*, 3H, CH_3_).
(3) *2-Amino-4,5-bis(4-ethylphenyl)furan-3-carbonitrile* (**3c**): White solid (75.1 mg, yield 79%), m.p. 156.0–158.0 °C; ^1^H NMR (600 MHz, CDCl_3_), *δ*, ppm: 7.36 (*d*, 2H, *J* = 8.4 Hz, ArH), 7.29 (*d*, 2H, *J* = 8.4 Hz, ArH), 7.23 (*d*, 2H, *J* = 8.4 Hz, ArH), 7.08 (*d*, 2H, *J* = 8.4 Hz, ArH), 5.00 (*s*, 2H, NH_2_), 2.67–2.72 (*q*, *J* = 7.8 Hz, 2H, CH_2_), 2.58–2.63 (*q*, *J* = 7.8 Hz, 2H, CH_2_), 1.28 (*t*, *J* = 7.8 Hz, 3H, CH_3_), 1.21 (*t*, *J* = 7.8 Hz, 3H, CH_3_); ^13^C NMR (150 MHz, CDCl_3_), *δ*, ppm: 161.8, 144.4, 143.7, 139.8, 129.0, 128.5, 128.0, 127.2, 125.4, 121.0, 115.5, 73.3, 28.8, 15.4; ESI-HRMS, *m*/*z*: Calcd for C_21_H_21_N_2_O [M + H]^+^: 317.1648, Found: 317.1644.
(4) *2-Amino-4,5-bis(4-isopropylphenyl)furan-3-carbonitrile* (**3d**): White solid (76.4 mg, yield 74%), m.p. 157.1–158.8 °C; ^1^H NMR (600 MHz, CDCl_3_), *δ*, ppm: 7.39 (*d*, 2H, *J* = 7.8 Hz, ArH), 7.32 (*d*, 2H, *J* = 7.8 Hz, ArH), 7.27 (*d*, 2H, *J* = 7.8 Hz, ArH), 7.12 (*d*, 2H, *J* = 7.8 Hz, ArH), 4.85 (*b*, 2H, NH_2_), 2.98–2.92 (*m*, 1H, CH), 2.90–2.84 (*m*, 1H, CH), 1.30 (*d*, *J* = 6.6 Hz, 6H, 2CH_3_), 1.23 (*d*, *J* = 7.2 Hz, 6H, 2CH_3_); ^13^C NMR (150 MHz, CDCl_3_), *δ*, ppm: 162.1, 154.5, 149.9, 135.2, 130.0, 128.9, 127.0, 126.6, 125.3, 113.7, 72.9, 34.0, 24.0; ESI-HRMS, *m*/*z*: Calcd for C_23_H_24_N_2_ONa [M+Na]^+^: 367.1786, Found: 367.1781.
(5) *2-Amino-4,5-bis(4-(tert-butyl)phenyl)furan-3-carbonitrile* (**3e**): White solid (81.2 mg, yield 73%), m.p. 178.4–180.4 °C; ^1^H NMR (600 MHz, CDCl_3_), *δ*, ppm: 7.42 (*d*, 2H, *J* = 8.4 Hz, ArH), 7.39 (d, 2H, *J* = 8.4 Hz, ArH), 7.34 (*d*, 2H, *J* = 8.4 Hz, ArH), 7.27 (*d*, 2H, *J* = 8.4 Hz, ArH), 5.03 (*s*, 2H, NH_2_), 1.36 (*s*, 9H, 3CH_3_), 1.30 (*s*, 9H, 3CH_3_); ^13^C NMR (150 MHz, CDCl_3_), *δ*, ppm: 161.9, 151.2, 150.5, 139.7, 128.7, 127.0, 125.9, 125.5, 125.0, 121.0, 115.6, 73.3, 34.8, 31.4; ESI-HRMS, *m*/*z*: Calcd for C_25_H_29_N_2_O [M + H]^+^: 373.2274, Found: 373.2272.
(6) *2-Amino-4,5-bis(4-fluorophenyl)furan-3-carbonitrile* (**3f**): White solid (77.3 mg, yield 87%), m.p. 210.3–211.9 °C (210.0–212.0 °C [51]); ^1^H NMR (600 MHz, DMSO-*d*_6_), *δ*, ppm: 7.73 (*s*, 2H, NH_2_), 7.44–7.40 (*m*, 2H, ArH), 7.33–7.29 (*m*, 2H, ArH), 7.25–7.22 (*m*, 2H, ArH), 7.16–7.12 (*m*, 2H, ArH).
(7) *2-Amino-4,5-bis(4-chlorophenyl)furan-3-carbonitrile* (**3g**): White solid (84.0 mg, yield 85%), m.p. 222.7–224.6 °C (223.0–225.0 °C [51]); ^1^H NMR (600 MHz, DMSO-*d*_6_), *δ*, ppm: 7.81 (*s*, 2H, NH_2_), 7.54 (*d*, 2H, *J* = 8.4 Hz, ArH), 7.41 (*d*, 2H, *J* = 8.4 Hz, ArH), 7.37 (*d*, 2H, *J* = 8.4 Hz, ArH), 7.21 (*d*, 2H, *J* = 8.4 Hz, ArH).
(8) *2-Amino-4,5-bis(4-bromophenyl)furan-3-carbonitrile* (**3h**): White solid (100.9 mg, yield 81%), m.p. 225.1–226.7 °C (226.0–227.0 °C [51]); ^1^H NMR (600 MHz, DMSO-*d*_6_), *δ*, ppm: 7.82 (*s*, 2H, NH_2_), 7.67 (*d*, 2H, *J* = 8.4 Hz, ArH), 7.49 (*d*, 2H, *J* = 8.4 Hz, ArH), 7.34 (*d*, 2H, *J* = 8.4 Hz, ArH), 7.14 (*d*, 2H, *J* = 8.4 Hz, ArH).
(9) *2-Amino-4,5-bis(4-bromophenyl)furan-3-carbonitrile* (**3i**): White solid (113.2 mg, yield 95%), m.p. 254.7–256.5 °C; ^1^H NMR (600 MHz, DMSO-*d*_6_), *δ*, ppm: 8.00 (*s*, 2H, NH_2_), 7.87 (*d*, 2H, *J* = 7.8 Hz, ArH), 7.66 (*d*, 2H, *J* = 8.4 Hz, ArH), 7.65 (*d*, 2H, *J* = 8.4 Hz, ArH), 7.38 (*d*, 2H, *J* = 7.8 Hz, ArH); ^13^C NMR (150 MHz, DMSO-*d*_6_), *δ*, ppm: 164.5, 136.2, 135.6, 133.2, 130.3, 129.5 (*q*, *J* = 32.3 Hz), 127.4 (*q*, *J* = 31.5 Hz), 126.6 (*q*, *J* = 4.1 Hz), 126.4 (*q*, *J* = 267.8 Hz), 126.3 (*q*, *J* = 4.2 Hz), 124.6 (*q*, *J* = 269.3 Hz), 123.7, 121.9, 115.4, 70.1; ^19^F NMR (564 MHz, DMSO-*d*_6_), *δ*, ppm: −61.178, −61.811; ESI-HRMS, *m*/*z*: Calcd for C_19_H_9_F_6_N_2_O [M − H]^−^: 395.0625, Found: 395.0626.
(10) *2-Amino-4,5-di-m-tolylfuran-3-carbonitrile* (**3j**): White solid (66.6 mg, yield 77%), m.p. 157.7–159.4 °C; ^1^H NMR (600 MHz, CDCl_3_), *δ*, ppm: 7.31–7.26 (*m*, 2H, ArH), 7.24 (*s*, 1H, ArH), 7.23 (*s*, 1H, ArH), 7.18 (*d*, *J* = 7.8 Hz, 1H, ArH), 7.14–7.09 (*m*, 2H, ArH), 7.02 (*d*, *J* = 7.2 Hz, 1H, ArH), 5.04 (*s*, 2H, NH_2_), 2.36 (*s*, 3H, CH_3_), 2.27 (*s*, 3H, CH_3_); ^13^C NMR (150 MHz, CDCl_3_), *δ*, ppm: 161.9, 139.7, 138.6, 138.2, 131.1, 129.7, 129.2, 128.4, 126.2, 125.9, 122.5, 121.8, 115.3, 73.4, 21.6, 21.5; ESI-HRMS, *m*/*z*: Calcd for C_19_H_17_N_2_O [M + H]^+^: 289.1335, Found: 289.1331.
(11) *2-Amino-4,5-bis(3-fluorophenyl)furan-3-carbonitrile* (**3k**): White solid (74.6 mg, yield 84%), m.p. 201.3–202.9 °C (201.0–202.0 °C [51]); ^1^H NMR (600 MHz, DMSO-*d*_6_), *δ*, ppm: 7.87 (*s*, 2H, NH_2_), 7.56–7.51 (*m*, 1H, ArH), 7.34–7.27 (*m*, 2H, ArH), 7.24 (*d*, *J* = 7.2 Hz, 2H, ArH), 7.04–7.00 (*m*, 2H, ArH), 6.94 (*d*, *J* = 8.4 Hz, 1H, ArH).
(12) *2-Amino-4,5-bis(3-chlorophenyl)furan-3-carbonitrile* (**3l**): White solid (80.4, yield 82%), m.p. 154.7–156.4 °C (154.0–156.0 °C [51]); ^1^H NMR (600 MHz, CDCl_3_), *δ*, ppm: 7.41 (*s*, 1H, ArH), 7.40–7.35 (*m*, 3H, ArH), 7.32–7.30 (*m*, 1H, ArH), 7.20–7.17 (*m*, 1H, ArH), 7.16–7.12 (*m*, 2H, ArH), 5.13 (*s*, 2H, NH_2_).
(13) *2-Amino-4,5-bis(3-bromophenyl)furan-3-carbonitrile* (**3m**): White solid (94.8 mg, yield 76%), m.p. 185.4–187.0 °C; ^1^H NMR (600 MHz, DMSO-*d*_6_), *δ*, ppm: 7.89 (*s*, 2H, NH_2_), 7.65 (*d*, 2H, *J* = 8.4 Hz, ArH), 7.59 (*s*, 1H, ArH), 7.46–7.43 (*m*, 1H, ArH), 7.41 (*d*, *J* = 7.8 Hz, 1H, ArH), 7.38–7.35 (*m*, 2H, ArH), 7.24–7.20 (*m*, 1H, ArH), 7.14 (*d*, *J* = 8.4 Hz, 1H, ArH); ^13^C NMR (150 MHz, DMSO-*d*_6_), *δ*, ppm: 164.2, 135.7, 133.6, 132.0, 131.8, 131.3, 130.1, 128.5, 127.0, 123.3, 122.6, 115.6, 69.8; ESI-HRMS, *m*/*z*: Calcd for C_17_H_11_N_2_OBr_2_ [M + H]^+^: 416.9238, Found: 416.9237.
(14) *2-Amino-4,5-bis(3-(trifluoromethyl)phenyl)furan-3-carbonitrile* (**3n**): White solid (95.0, yield 80%), m.p. 253.7–255.4 °C; ^1^H NMR (600 MHz, CDCl_3_), *δ*, ppm: 7.70–7.67 (*m*, 3H, ArH), 7.65 (*d*, *J* = 7.8 Hz, 1H, ArH), 7.61 (*s*, 1H, ArH), 7.60–7.57 (*m*, 1H, ArH), 7.46 (*d*, *J* = 7.2 Hz, 1H, ArH), 7.42 (*d*, *J* = 7.8 Hz, 1H, ArH), 7.34 (*t*, *J* = 7.8 Hz, 1H, ArH), 5.27 (*s*, 2H, NH_2_); ^13^C NMR (150 MHz, CDCl_3_), *δ*, ppm: 162.3, 138.4, 132.3, 131.5 (*q*, *J* = 247.4 Hz), 131.4 (*q*, *J* = 243.0 Hz), 129.8, 129.1, 127.9, 125.7 (*q*, *J* = 43.4 Hz), 125.6 (*q*, *J* = 42.5 Hz), 124.6, 124.2 (*q*, *J* = 3.9 Hz), 122.8, 121.8 (*q*, *J* = 4.5 Hz), 114.4, 72.9; ^19^F NMR (564 MHz, CDCl_3_), *δ*, ppm: −62.910, −63.205; ESI-HRMS, *m*/*z*: Calcd for C_19_H_9_F_6_N_2_O [M − H]^−^: 395.0625, Found: 395.0622.
(15) *2-Amino-4,5-bis(4-bromophenyl)furan-3-carbonitrile* (**3o**): White solid (61.6 mg, yield 65%), m.p. 170.2–172.1 °C; ^1^H NMR (600 MHz, DMSO-*d*_6_), *δ*, ppm: 7.64 (*s*, 2H, NH_2_), 7.03 (*s*, 1H, ArH), 6.99 (*s*, 2H, ArH), 6.89 (*s*, 2H, ArH), 6.80 (*s*, 1H, ArH), 2.26 (*s*, 6H, CH_3_), 2.12 (*s*, 6H, CH_3_); ^13^C NMR (150 MHz, DMSO-*d*_6_), *δ*, ppm: 164.0, 138.3, 137.3, 131.6, 130.2, 128.9, 127.0, 123.7, 122.5, 116.2, 69.6, 21.4, 21.3; ESI-HRMS, *m*/*z*: Calcd for C_21_H_21_N_2_O [M + H]^+^: 317.1648, Found: 317.1644.
(16) *2-Amino-4,5-bis(3,5-difluorophenyl)furan-3-carbonitrile* (**3p**): White solid (70.9, yield 71%), m.p. 228.3–230.2 °C; ^1^H NMR (600 MHz, DMSO-*d*_6_), *δ*, ppm: 8.00 (*s*, 2H, NH_2_), 7.39 (*s*, 1H, ArH), 7.18 (*s*, 2H, ArH), 7.09 (*s*, 1H, ArH), 6.79 (*s*, 2H, ArH); ^13^C NMR (150 MHz, DMSO-*d*_6_), *δ*, ppm: 164.1, 163.2 (*d*, *J* = 246.3 Hz), 163.1 (*d*, *J* = 246.9 Hz), 163.0 (*d*, *J* = 244.4 Hz), 162.9 (*d*, *J* = 244.1 Hz), 135.2 (*d*, *J* = 3.5 Hz), 134.4, 132.3, 123.0 (*d*, *J* = 3.2 Hz), 115.1, 112.9 (*d*, *J* = 25.4 Hz), 107.3 (*d*, *J* = 27.5 Hz), 105.2 (*d*, *J* = 25.7 Hz), 105.0 (*d*, *J* = 25.4 Hz), 103.0 (*d*, *J* = 26.0 Hz), 102.8 (*d*, *J* = 26.0 Hz), 70.1; ^19^F NMR (564 MHz, DMSO-*d*_6_), *δ*, ppm: −108.408, −108.771; ESI-HRMS, *m*/*z*: Calcd for C_17_H_7_F_4_N_2_O [M − H]^−^: 331.0500, Found: 331.0499.
(17) *2-Amino-4,5-bis(3,5-dichlorophenyl)furan-3-carbonitrile* (**3q**): White solid (80.8, yield 68%), m.p. 243.6–245.5 °C; ^1^H NMR (600 MHz, DMSO-*d*_6_), *δ*, ppm: 8.03 (*s*, 2H, NH_2_), 7.74 (*s*, 1H, ArH), 7.50 (*s*, 2H, ArH), 7.41 (*s*, 1H, ArH), 7.11 (*s*, 2H, ArH); ^13^C NMR (150 MHz, DMSO-*d*_6_), *δ*, ppm: 164.3, 135.3, 135.0, 134.7, 134.3, 132.3, 129.1, 128.1, 126.6, 122.6, 115.1, 70.0; ESI-HRMS, *m*/*z*: Calcd for C_17_H_8_N_2_ONaCl_4_ [M+Na]^+^: 418.9288, Found: 418.9286.
(18) *2-Amino-4-(4-(tert-butyl)phenyl)-5-(4-ethylphenyl)furan-3-carbonitrile* (**3r**): White solid (72.2 mg, yield 70%), m.p. 188.0–190.0 °C; ^1^H NMR (600 MHz, CDCl_3_), *δ*, ppm: 7.43–7.40 (*m*, 1H, ArH), 7.39–7.35 (*m*, 2H, ArH), 7.33–7.30 (*m*, 2H, ArH), 7.28–7.26 (*m*, 1H, ArH), 7.25–7.22 (*m*, 1H, ArH), 7.10–7.07 (*m*, 1H, ArH), 5.00 (*s*, 2H, NH_2_), 2.72–2.59 (*m*, 2H, CH_2_), 1.36 (*s*, 3H, CH_3_), 1.31–1.27 (*m*, 9H, 3CH_3_); ^13^C NMR (150 MHz, CDCl_3_), *δ*, ppm: 162.0, 156.9, 151.2, 150.5, 144.4, 139.6, 129.8, 129.0, 128.0, 125.5, 125.0, 113.7, 73.2, 35.3, 31.3, 28.8, 15.4, ESI-HRMS, *m*/*z*: Calcd for C_23_H_25_N_2_O [M + H]^+^: 345.1961, Found: 345.1958.
(19) *2-Amino-4-(4-bromophenyl)-5-(4-chlorophenyl)furan-3-carbonitrile* (**3s**): White solid (80.6 mg, yield 72%), m.p. 235.2–236.9 °C; ^1^H NMR (600 MHz, DMSO-*d*_6_), *δ*, ppm: 7.83 (*s*, 2H, NH_2_), 7.68 (*d*, 1H, *J* = 8.4 Hz, ArH), 7.54 (*d*, 1H, *J* = 9.0 Hz, ArH), 7.50 (*d*, 1H, *J* = 7.2 Hz, ArH), 7.41 (*d*, 1H, *J* = 8.4 Hz, ArH), 7.37 (*d*, 1H, *J* = 7.2 Hz, ArH), 7.34 (*d*, 1H, *J* = 8.4 Hz, ArH), 7.23–7.20 (*m*, 1H, ArH), 7.16–7.13 (*m*, 1H, ArH); ^13^C NMR (150 MHz, DMSO-*d*_6_), *δ*, ppm: 164.2, 136.3, 133.8, 132.7, 131.4, 130.7, 129.7, 128.8, 126.7, 122.4, 120.5, 115.7, 69.6; ESI-HRMS, *m*/*z*: Calcd for C_17_H_9_BrClN_2_O [M − H]^−^: 370.9592, Found: 370.9593.
(20) *5′-Amino-[2,2′:3′,2″-terfuran]-4′-carbonitrile* (**3t**): Brown solid (48.2 mg, yield 67%), m.p. 186.2–187.9 °C (187.0–188.0 °C [48]); ^1^H NMR (600 MHz, CDCl_3_), *δ*, ppm: 7.86 (*s*, 2H, NH_2_), 7.81 (*d*, *J* = 2.4 Hz, 1H, ArH), 7.75 (*d*, *J* = 2.4 Hz, 1H, ArH), 6.85 (*d*, *J* = 2.4 Hz, 1H, ArH), 6.74 (*d*, *J* = 2.4 Hz, 1H, ArH), 6.64–6.63 (*m*, 1H, ArH), 6.62–6.60 (*m*, 1H, ArH).
(21) *4,5-Bi([1,1′-biphenyl]-4-yl)-2-aminofuran-3-carbonitrile* (**3u**): White solid (119.9 mg, yield 97%), m.p. 237.1–239.1 °C; ^1^H NMR (600 MHz, DMSO-*d*_6_), *δ*, ppm: 7.80 (*d*, *J* = 8.4 Hz, 2H, ArH), 7.79 (*s*, 2H, NH_2_), 7.75 (*d*, *J* = 8.4 Hz, 2H, ArH), 7.63 (*d*, *J* = 7.2 Hz, 2H, ArH), 7.61 (*d*, *J* = 8.4 Hz, 2H, ArH), 7.52 (*d*, *J* = 8.4 Hz, 2H, ArH), 7.51–7.48 (*m*, 2H, ArH), 7.44–7.41 (*m*, 3H, ArH), 7.38 (*d*, *J* = 8.4 Hz, 2H, ArH), 7.33 (*t*, *J* = 7.2 Hz, 1H, ArH); ^13^C NMR (150 MHz, DMSO-*d*_6_), *δ*, ppm: 164.2, 140.4, 139.8, 138.8, 137.1, 130.8, 130.0, 129.5, 129.0, 128.3, 127.7, 127.3, 127.1, 126.8, 125.2, 122.2, 116.1, 69.8; ESI-HRMS, *m*/*z*: Calcd for C_29_H_21_N_2_O [M + H]^+^: 413.1648, Found: 413.1643.
(22) *1-(2-Amino-4,5-diphenylfuran-3-yl)propan-1-one* (**3v**): White solid (43.3 mg, yield 47%), m.p. 161.2–163.0 °C (163.0–165.0 °C [53]); ^1^H NMR (600 MHz, DMSO-*d*_6_), *δ*, ppm: 7.40–7.34 (*m*, 3H, ArH), 7.29–7.28 (*m*, 2H, ArH), 7.27 (*s*, 2H, NH_2_), 7.20–7.16 (*m*, 2H, ArH), 7.11–7.07 (*m*, 3H, ArH), 3.92 (*q*, *J* = 7.2 Hz, 2H, OCH_2_), 0.91 (*t*, *J* = 7.2 Hz, 3H, CH_3_).
(23) *Ethyl 2-amino-4,5-di-p-tolylfuran-3-carboxylate* (**3w**): White solid (37.2 mg, yield 37%), m.p. 147.0–148.8 °C; ^1^H NMR (600 MHz, CDCl_3_), *δ*, ppm: 7.21 (*d*, *J* = 7.8 Hz, 2H, ArH), 7.16 (*d*, *J* = 7.8 Hz, 4H, ArH), 7.15 (*d*, *J* = 7.2 Hz, 2H, ArH), 6.98 (*d*, *J* = 7.2 Hz, 2H, ArH), 4.06 (*q*, *J* = 7.2 Hz, 2H, OCH_2_), 2.39 (*s*, 3H, CH_3_), 2.26 (*s*, 3H, CH_3_), 1.03 (*t*, *J* = 7.2 Hz, 3H, CH_3_); ^13^C NMR (150 MHz, CDCl_3_), *δ*, ppm: 163.5, 158.9, 140.7, 136.8, 130.1, 129.9, 129.4, 128.9, 128.7, 124.7, 128.3, 89.2, 59.2, 21.4, 14.0; ESI-HRMS, *m*/*z*: Calcd for C_21_H_22_NO_3_ [M + H]^+^: 336.1594, Found: 336.1589.
(24) *Ethyl 2-amino-4,5-bis(4-ethylphenyl)furan-3-carboxylate* (**3x**): White solid (37.0 mg, yield 34%), m.p. 157.0–159.7 °C; ^1^H NMR (600 MHz, CDCl_3_), *δ*, ppm: 7.24 (*d*, *J* = 8.4 Hz, 2H, ArH), 7.19 (*d*, *J* = 8.4 Hz, 4H, ArH), 7.02 (*d*, *J* = 8.4 Hz, 2H, ArH), 4.03 (*q*, *J* = 7.2 Hz, 2H, OCH_2_), 2.70 (*q*, *J* = 7.8 Hz, 2H, CH_2_), 2.60 (*q*, *J* = 7.2 Hz, 2H, CH_2_), 1.27 (*t*, *J* = 7.8 Hz, 3H, CH_3_), 1.18 (*t*, *J* = 7.8 Hz, 3H, CH_3_), 0.99 (*t*, *J* = 7.2 Hz, 3H, CH_3_); ^13^C NMR (150 MHz, CDCl_3_), *δ*, ppm: 163.6, 162.6, 146.8, 145.0, 134.7, 130.3, 128.9, 128.2, 127.7, 127.5, 125.8, 89.3, 61.7, 28.5, 15.0, 13.9; ESI-HRMS, *m*/*z*: Calcd for C_23_H_26_NO_3_ [M + H]^+^: 364.1907, Found: 364.1902.
(25) *Ethyl 2-amino-4,5-bis(4-chlorophenyl)furan-3-carboxylate* (**3y**): White solid (61.9 mg, yield 55%), m.p. 170.0–172.0 °C; ^1^H NMR (600 MHz, CDCl_3_), *δ*, ppm: 7.36 (*d*, *J* = 8.4 Hz, 2H, ArH), 7.26 (*s*, 2H, NH_2_), 7.25 (*d*, *J* = 8.4 Hz, 2H, ArH), 7.15 (*d*, *J* = 9.0 Hz, 2H, ArH), 7.12 (*d*, *J* = 9.0 Hz, 2H, ArH), 4.06 (*q*, *J* = 7.2 Hz, 2H, OCH_2_), 1.03 (*t*, *J* = 7.2 Hz, 3H, CH_3_); ^13^C NMR (150 MHz, CDCl_3_), *δ*, ppm: 165.2, 161.7, 138.6, 133.7, 132.0, 131.7, 130.0, 128.7, 128.6, 125.9, 92.2, 59.6, 14.1; ESI- HRMS, *m*/*z*: Calcd for C_19_H_16_Cl_2_NO_3_ [M + H]^+^: 376.0502, Found: 376.0496.
(26) *Ethyl 2-amino-4,5-bis(4-bromophenyl)furan-3-carboxylate* (**3z**): White solid (72.2 mg, yield 52%), m.p. 155.2–156.8 °C; ^1^H NMR (600 MHz, CDCl_3_), *δ*, ppm: 7.51 (*d*, *J* = 8.4 Hz, 2H, ArH), 7.30 (*d*, *J* = 8.4 Hz, 2H, ArH), 7.19 (*d*, *J* = 8.4 Hz, 2H, ArH), 7.06 (*d*, *J* = 8.4 Hz, 2H, ArH), 5.71 (*b*, 2H, NH_2_), 4.06 (*q*, *J* = 7.2 Hz, 2H, OCH_2_), 1.03 (*t*, *J* = 7.2 Hz, 3H, CH_3_); ^13^C NMR (150 MHz, CDCl_3_), *δ*, ppm: 165.2, 161.7, 138.6, 132.5, 132.0, 131.6, 131.5, 126.2, 121.9, 92.2, 59.6, 14.1; ESI-HRMS, *m*/*z*: Calcd for C_19_H_16_Br_2_NO_3_ [M + H]^+^: 463.9491, Found: 463.9461.
(27) *Ethyl 2-amino-4,5-bis(3-fluorophenyl)furan-3-carboxylate* (**3aa**): White solid (49.4 mg, yield 48%), m.p. 157.2–158.8 °C; ^1^H NMR (600 MHz, CDCl_3_), *δ*, ppm: 7.37–7.33 (*m*, 1H, ArH), 7.14–7.05 (*m*, 4H, ArH), 6.95–6.91 (*m*, 2H, ArH), 6.82–6.78 (*m*, 1H, ArH), 5.74 (*b*, 2H, NH_2_), 4.05 (*q*, *J* = 7.2 Hz, 2H, OCH_2_), 1.00 (*t*, *J* = 7.2 Hz, 3H, CH_3_); ^13^C NMR (150 MHz, CDCl_3_), *δ*, ppm: 165.1, 162.7 (*d*, *J* = 243.2 Hz), 162.6 (*d*, *J* = 244.4 Hz), 161.6, 138.2, 135.5 (*d*, *J* = 8.6 Hz), 132.1 (*d*, *J* = 8.6 Hz), 129.9 (*d*, *J* = 8.6 Hz), 129.7 (*d*, *J* = 8.3 Hz), 125.8 (*d*, *J* = 2.7 Hz), 121.6, 120.0 (*d*, *J* = 2.9 Hz), 117.2 (*d*, *J* = 21.5 Hz), 114.6 (*d*, *J* = 20.1 Hz), 113.3 (*d*, *J* = 21.3 Hz), 111.2 (*d*, *J* = 24.2 Hz), 92.3, 59.4, 13.8; ^19^F NMR (564 MHz, CDCl_3_), *δ*, ppm: −112.718, −113.766; ESI-HRMS, *m*/*z*: Calcd for C_19_H_16_F_2_NO_3_ [M + H]^+^: 344.1093, Found: 344.1089.
(28) *N-Acetyl-N-(3-cyano-4,5-diphenylfuran-2-yl)acetamide* (**4a**): White solid (66.1 mg, yield 64%), m.p. 157.6–159.5 °C (156.0–158.0 °C [48]); ^1^H NMR (600 MHz, DMSO-*d*_6_), *δ*, ppm: 7.55–7.49 (*m*, 5H, ArH), 7.46–7.43 (*m*, 2H, ArH), 7.40–7.39 (*m*, 3H, ArH), 2.43 (*s*, 6H, 2CH_3_).
(29) *2-Methyl-5,6-diphenyl-4H-furo[2,3-d]*[1,3]*oxazin-4-one* (**4b**): White solid (55.4 mg, yield 61%), m.p. 196.7–198.5 °C (196.0–198.0 °C [49]); ^1^H NMR (600 MHz, DMSO-*d*_6_), *δ*, ppm: 7.52–7.45 (*m*, 3H, ArH), 7.43–7.40 (*m*, 2H, ArH), 7.36–7.29 (*m*, 5H, ArH), 2.16 (*s*, 3H, CH3).
(30) *(E)-2-((4-Methoxybenzylidene)amino)-4,5-diphenylfuran-3-carbonitrile* (**4c**): Yellow solid (76.0 mg, yield 67%), m.p. 168.7–170.5 °C; ^1^H NMR (600 MHz, DMSO-*d*_6_), *δ*, ppm: 9.09 (*s*, 1H, CH), 8.04 (*d*, *J* = 9.0 Hz, 2H, ArH), 7.55–7.49 (*m*, 5H, ArH), 7.48–7.47 (*m*, 2H, ArH), 7.41–7.34 (*m*, 3H, ArH), 7.15 (*d*, *J* = 9.0 Hz, 2H, ArH), 3.88 (*s*, 3H, OCH_3_); ^13^C NMR (150 MHz, DMSO-*d*_6_), *δ*, ppm: 164.1, 161.8, 160.2, 145.2, 132.4, 132.3, 130.5, 129.7, 129.5, 129.3, 129.0, 128.3, 126.4, 115.4, 113.9, 92.7, 56.2; ESI-HRMS, *m*/*z*: Calcd for C_25_H_19_N_2_O_2_ [M + H]^+^: 379.1441, Found: 379.1442.
(31) *(E)-2-((4-Fluorobenzylidene)amino)-4,5-diphenylfuran-3-carbonitrile* (**4d**): Yellow solid (77.0 mg, yield 70%), m.p. 173.4–175.3 °C; ^1^H NMR (600 MHz, DMSO-*d*_6_), *δ*, ppm: 9.13 (*s*, 1H, CH), 8.19–8.09 (*m*, 2H, ArH), 7.53–7.43 (*m*, 10H, ArH), 7.42–7.33 (*m*, 2H, ArH); ^13^C NMR (150 MHz, DMSO-*d*_6_), *δ*, ppm: 165.5 (*d*, *J* = 251.4 Hz), 161.1, 159.4, 145.8, 132.7 (*d*, *J* = 9.5 Hz), 132.2, 130.3, 129.7, 129.5, 129.3, 128.8, 126.5, 124.0, 117.0 (*d*, *J* = 22.1 Hz), 113.5, 94.2; ^19^F NMR (564 MHz, DMSO-*d*_6_), *δ*, ppm: −105.189; ESI-HRMS, *m*/*z*: Calcd for C_24_H_16_FN_2_O [M + H]^+^: 367.1241, Found: 367.1242.
(32) *(E)-4,5-Diphenyl-2-((4-trifluoromethylbenzylidene)amino)furan-3-carbonitrile* (**4e**): Yellow solid (90.0 mg, yield 72%), m.p. 176.7–178.4 °C; ^1^H NMR (600 MHz, DMSO-*d*_6_), *δ*, ppm: 9.22 (*s*, 1H, CH), 8.25 (*d*, *J* = 8.4 Hz, 2H, ArH), 7.93 (*d*, *J* = 8.4 Hz, 2H, ArH), 7.55- 7.51 (*m*, 5H, ArH), 7.49–7.47 (*m*, 2H, ArH), 7.41–7.37 (*m*, 3H, ArH); ^13^C NMR (150 MHz, DMSO-*d*_6_), *δ*, ppm: 160.7, 158.8, 146.4, 139.0, 132.5 (*q*, *J* = 32.0 Hz), 130.6, 130.2, 129.7, 129.5, 129.3, 128.7, 126.6, 126.5 (*q*, *J* = 3.9 Hz), 125.3 (*q*, *J* = 271.2 Hz), 113.3, 95.8; ^19^F NMR (564 MHz, DMSO-*d*_6_), *δ*, ppm: −61.483; ESI-HRMS, *m*/*z*: Calcd for C_25_H_16_F_3_N_2_O [M + H]^+^: 417.1209, Found: 417.1205.

The detailed ^1^H, ^13^C and ^19^F NMR spectra for all compounds **3a**–**3aa** and **4a**–**4e** are provided in the Supplementary Materials.

## 4. Conclusions

In summary, we have successfully developed an efficient and straightforward method for the synthesis of a series of tetrasubstituted furan compounds. The reaction proceeds under mild conditions with a lower reaction temperature and less reaction time, without the need for expensive transition metal catalysts or strong oxidants, and affords the desired products in moderate to high yields.

Importantly, the developed green conditions for synthesizing tetrasubstituted furans can be successfully applied to the Gram-scale reaction with a high yield. Additionally, the synthesized target compounds can conduct different functional group derivatization. These results further validate the practical application value of this transformation and its target compounds.

## Figures and Tables

**Table 1:** Optimization of reaction conditions ^[a]^.

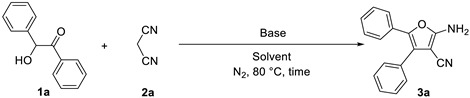				
Entry	Base	Solvent	Time (h)	Yield (%) ^[b]^
1 ^[c]^	CsF	EtOH	3	47
2	CsF	EtOH	3	90
3 ^[d]^	CsF	EtOH	3	72
4	Cs_2_CO_3_	EtOH	3	Trace
5	NaOH	EtOH	3	41
6	Ba(OH)_2_	EtOH	3	45
7	AcONa	EtOH	3	40
8	K_2_CO_3_	EtOH	3	37
9	Na_2_CO_3_	EtOH	3	52
10	NaHCO_3_	EtOH	3	43
11	*^t^*BuOK	EtOH	3	33
12	*^t^*BuONa	EtOH	3	37
13	DBU	EtOH	3	23
14	TEA	EtOH	3	53
15	Imidazole	EtOH	3	57
16	2-MI	EtOH	3	61
17	Pyridine	EtOH	3	43
18	CsF	Toluene (110) ^[e]^	3	65
19	CsF	H_2_O (100)	3	42
20	CsF	MeOH (70)	3	82
21	CsF	DMF (110)	3	87
22	CsF	DMSO (110)	3	89
23	CsF	THF (70)	3	67
24	CsF	MeCN (80)	3	79
25	CsF	1,4-Dioxane (100)	3	47
26	CsF	DCM (40)	3	43
27	CsF	DEM (90)	3	52
28	CsF	Xylene (110)	3	68
29	CsF	EtOH (70)	3	85
30	CsF	EtOH (60)	3	79
31	CsF	EtOH	4	94
32	CsF	EtOH	5	90
33 ^[f]^	CsF	EtOH	4	73
34 ^[g]^	CsF	EtOH	4	76
35 ^[h]^	CsF	EtOH	4	77
36 ^[i]^	CsF	EtOH	4	91

Reaction conditions: ^[a]^ Usually, **1a** (0.3 mmol), **2a** (0.6 mmol), and base (0.9 mmol) for the reaction under N_2_ atmosphere, with a molar ratio of **1a**:**2a**:Base at 1:2:3 with 5 mL solvent. ^[b]^ Isolated yield. ^[c]^ Air atmosphere. ^[d]^ Reacting in a Schlenk pressure tube. ^[e]^ The number displayed in parentheses is the temperature (Temp., °C) during the reaction. ^[f]^ **1a**:**2a**:Base = 1:1.5:3. ^[g]^ **1a**:**2a**:Base = 1:2.5:3. ^[h]^ **1a**:**2a**:Base = 1:2:2.5. ^[i]^ **1a**:**2a**:Base = 1:2:3.5.

**Table 2:** Substrate applicability of different *α*-hydroxy ketones ^[a]^.

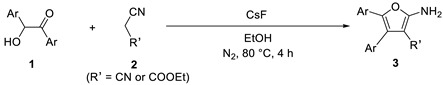
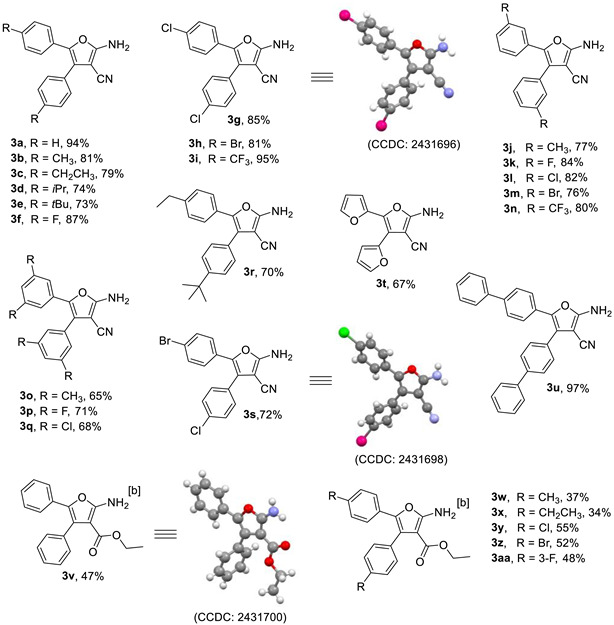

Reaction conditions: ^[a]^ **1** (0.3 mmol), **2a** (0.6 mmol), CsF (0.9 mmol), added in EtOH (5 mL) and stirred at 80 °C for 4 h under N_2_ atmosphere. ^[b]^ **1** (0.3 mmol), **2b** (0.6 mmol), piperidine (0.9 mmol), added in Toluene (3 mL) and stirred at 80 °C for 8 h under N_2_ atmosphere.

## Data Availability

All data supporting the findings of this study are available within the paper and within its Supplementary Materials published online.

## Supplementary Materials

The following supporting information can be downloaded at: https://www.mdpi.com/article/10.3390/molecules30081832/s1, and contains the ^1^H, ^13^C and ^19^F NMR spectra for all compounds **3a**–**3aa** and **4a**–**4e**. Table S1. Data of Single-crystal X-ray Analysis for **3g**. Table S2. Data of Single-crystal X-ray Analysis for **3s**. Table S3. Data of Single-crystal X-ray Analysis for **3v**. Figure S1. The molecular structure of **3g**. Figure S2. The molecular structure of **3s**. Figure S3. The molecular structure of **3v**.
