# Supplementary material for: Metal-Free Catalytic Synthesis of Tetrasubstituted Furans from α-Hydroxy Ketones and Cyano Compounds
Source: Molecules. 2025 Apr 19;30(8):1832. doi: 10.3390/molecules30081832 (PMC12029176; doi:10.3390/molecules30081832)
Supplement: Supplementary file 1 [file molecules-30-01832-s001.zip › molecules-3574330-supplementary.pdf]

Supporting Information

Metal-Free Catalytic Synthesis of Tetrasubstituted Furans from α-Hydroxy Ketones and Cyano Compounds

Yu Zeng, Shi-Hang Yang, Ji-Lin Guo, Yun Li, Ting Lin and Zhao-Yang Wang *

School of Chemistry, South China Normal University, Guangzhou Key Laboratory of Analytical Chemistry for Biomedicine, GDMPA Key Laboratory for Process Control and Quality Evaluation of Chiral Pharma-ceuticals, Key Laboratory of Theoretical Chemistry of Environment, Ministry of Education, Guangzhou 510006, China; 2023022534@m.scnu.edu.cn (Y.Z.); 2024022743@m.scnu.edu.cn (S.-H.Y.); 2024022665@m.scnu.edu.cn (J.-L.G.); 2024022879@m.scnu.edu.cn (Y.L.); 20232421068@m.scnu.edu.cn (T.L.)

* Correspondence: wangzy@scnu.edu.cn; Tel.: +86-020-3931-0258; Fax: +86-020-3931-0187

Table of Contents

Results of Single-crystal X-ray Analysis for **3g**, **3s**, **3v**……………………………...………......…[2-4]

Characterization Data for All Products **3a-3aa**, **4a-4e**………………………..…..……………[5-17]

NMR Spectra for All Products **3a-3aa**, **4a-4e**…………………………….…………………[18-68]

References……………………………………………………………………….…………….…[69]

Results of Single-crystal X-ray Analysis for 3g, 3s, 3v

Table S1 Data of Single-crystal X-ray Analysis for **3g**

**Compound**	**3g**
Empirical formula	$C_{17}H_{10}Cl_2N_2O$
Formula weight	329.17
Temperature (K)	297 K
Crystal system	triclinic
Space group	P-1
Unit cell dimensions (Å, °)	a = 7.6103(9), b = 10.1198(11), c = 19.235(2)
	α = 102.787(3), β = 90.881(3), γ = 90.950(3)
Volume (Å^3)	1444.2(3)
Z	4
Density (calculated) (g/cm^3)	1.767
Absorption coefficient (mm^{-1})	12.824
F(000)	752.0
Theta range for data collection	2.049 to 57.283
Reflections collected	5821
Independent reflections	5821 [R_{int} = 0.1007, R_{sigma} = 0.0573]
Max. and min. transmission	0.751 and 0.238
Refinement method	Least Squares minimisation
Data / restraints / parameters	5821/0/346
Goodness-of-fit on F^2	1.080
Final R indices [I>2sigma(I)]	R_1 = 0.0631, wR_2 = 0.1696
R indices (all data)	R_1 = 0.0691, wR_2 = 0.1728
Largest diff. peak and hole	1.91/-2.00 e.Å-3

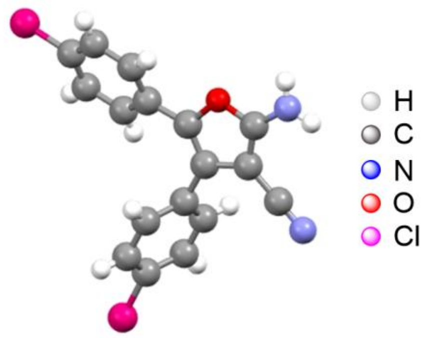

Figure S1 The molecular structure of **3g**

Table S2 Data of Single-crystal X-ray Analysis for **3s**

**Compound**	**3s**
Empirical formula	$C_{17}H_{10}BrClN_2O$
Formula weight	373.63
Temperature (K)	297 K
Crystal system	triclinic
Space group	P-1
Unit cell dimensions (Å,°)	a = 6.0350(9), b = 8.7650(14), c = 15.0045(18)
	α = 82.534(12), β = 87.877(11), γ = 84.015(13)
Volume ($Å^3$)	782.4(2)
Z	2
Density (calculated) (g/cm^3)	1.586
Absorption coefficient (mm^{-1})	5.170
F(000)	372.0
Theta range for data collection	5.942 to 142.388
Reflections collected	4765
Independent reflections	2944 [R_{int} = 0.0619, R_{sigma} = 0.0992]
Max. and min. transmission	0.751 and 0.238
Refinement method	Least Squares minimisation
Data / restraints / parameters	2944/0/200
Goodness-of-fit on F^2	1.007
Final R indices [I>2sigma(I)]	R_1 = 0.0837, wR_2 = 0.2072
R indices (all data)	R_1 = 0.1170, wR_2 = 0.2523
Largest diff. peak and hole	e Å-3 0.71/-0.72

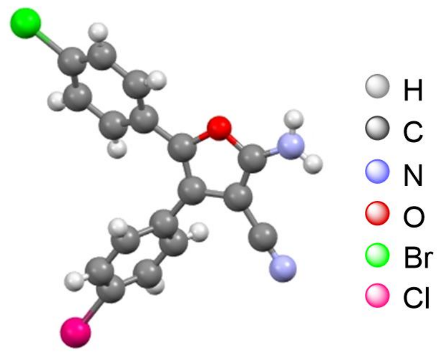

Figure S2 The molecular structure of **3s**

Table S3 Data of Single-crystal X-ray Analysis for **3v**

**Compound**	**3v**
Empirical formula	$C_{19}H_{17}NO_3$
Formula weight	307.33
Temperature (K)	297 K
Crystal system	orthorhombic
Space group	$P2_12_12_1$
Unit cell dimensions (Å,°)	a = 8.0626(4), 8.6426(5), c = 23.4607(18)
	α = 90, β = 90, γ = 90
Volume (Å^3)	1634.78(18)
Z	4
Density (calculated) (g/cm^3)	1.249
Absorption coefficient (mm^{-1})	0.686
F(000)	648.0
Radiation	Cu Kα (λ = 1.54184)
Theta range for data collection	7.536 to 142.04
Index ranges	$-9 \leq h \leq 7$, $-10 \leq k \leq 10$, $-28 \leq l \leq 25$
Reflections collected	5323
Independent reflections	3082 [R_{int} = 0.0332, R_{sigma} = 0.0528]
Refinement method	Least Squares minimisation
Data / restraints / parameters	3082/0/210
Goodness-of-fit on F^2	1.041
Final R indices [I>2sigma(I)]	R_1 = 0.0487, wR_2 = 0.1233
R indices (all data)	R_1 = 0.0578, wR_2 = 0.1335
Largest diff. peak and hole	e Å-3 0.16/-0.16

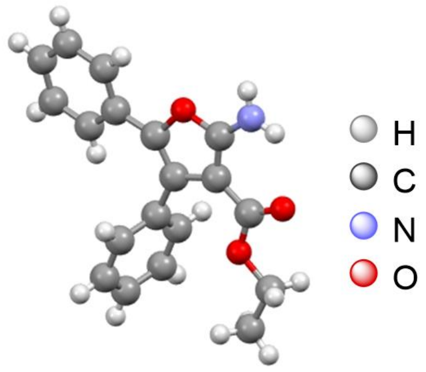

Figure S3 The molecular structure of **3v**

Characterization Data for All Products 3a-3aa, 4a-4e

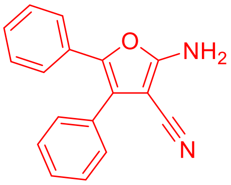

2-Amino-4,5-diphenylfuran-3-carbonitrile (**3a**): White solid (72.2 mg, yield 93%), m.p. 205.2-205.9 ºC (204.0-206.0 ºC[1]); ^{1}H NMR (600 MHz, DMSO-d_6), δ, ppm: 7.72 (*s*, 2H, NH_2), 7.48-7.44 (*m*, 2H, ArH), 7.43-7.40 (*m*, 1H, ArH), 7.39-7.37 (*m*, 2H, ArH), 7.27-7.22 (*m*, 4H, ArH), 7.19-7.16 (*m*, 1H, ArH).

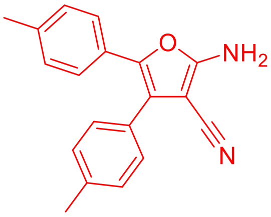

2-Amino-4,5-di-*p*-tolylfuran-3-carbonitrile (**3b**): White solid (71.7 mg, yield 81%), m.p. 214.8-216.6 ºC (216.0 ºC[2]); ^{1}H NMR (600 MHz, $CDCl_3$), δ, ppm: 7.33 (*d*, 2H, J = 7.8 Hz, ArH), 7.27 (*d*, 2H, J = 7.8 Hz, ArH), 7.21 (*d*, 2H, J = 7.8 Hz, ArH), 7.06 (*d*, 2H, J = 7.8 Hz, ArH), 4.99 (*s*, 2H, NH_2), 2.40 (*s*, 3H, CH_3), 2.32 (*s*, 3H, CH_3).

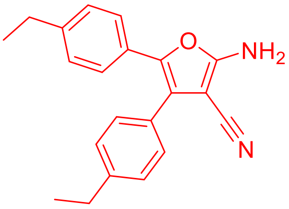

2-Amino-4,5-bis(4-ethylphenyl)furan-3-carbonitrile (**3c**): White solid (75.1 mg, yield 79%), m.p. 156.0-158.0 ºC; ^{1}H NMR (600 MHz, $CDCl_3$), δ, ppm: 7.36 (*d*, 2H, J = 8.4 Hz, ArH), 7.29 (*d*, 2H, J = 8.4 Hz, ArH), 7.23 (*d*, 2H, J = 8.4 Hz, ArH), 7.08 (*d*, 2H, J = 8.4 Hz, ArH), 5.00 (*s*, 2H, NH_2), 2.67-2.72 (*q*, J = 7.8 Hz, 2H, CH_2), 2.58-2.63 (*q*, J = 7.8 Hz, 2H, CH_2), 1.28 (*t*, J = 7.8 Hz, 3H, CH_3), 1.21 (*t*, J = 7.8 Hz, 3H, CH_3); ^{13}C NMR (150 MHz, $CDCl_3$), δ, ppm: 161.8, 144.4, 143.7, 139.8, 129.0, 128.5, 128.0, 127.2, 125.4, 121.0, 115.5, 73.3, 28.8, 15.4; ESI-HRMS, *m/z*: Calcd for

$C_{21}H_{21}N_2O$ [M+H]$^+$: 317.1648, Found: 317.1644.

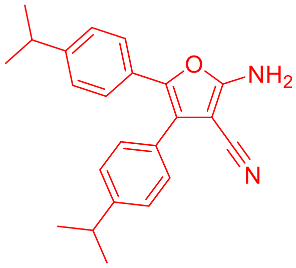

2-Amino-4,5-bis(4-isopropylphenyl)furan-3-carbonitrile (**3d**): White solid (76.4 mg, yield 74%), m.p. 157.1-158.8 °C; ^{1}H NMR (600 MHz, $CDCl_3$), δ, ppm: 7.39 (*d*, 2H, *J* = 7.8 Hz, ArH), 7.32 (*d*, 2H, *J* = 7.8 Hz, ArH), 7.27 (*d*, 2H, *J* = 7.8 Hz, ArH), 7.12 (*d*, 2H, *J* = 7.8 Hz, ArH), 4.85 (*b*, 2H, NH_2), 2.98-2.92 (*m*, 1H, CH), 2.90-2.84 (*m*, 1H, CH), 1.30 (*d*, *J* = 6.6 Hz, 6H, $2CH_3$), 1.23 (*d*, *J* = 7.2 Hz, 6H, $2CH_3$); ^{13}C NMR (150 MHz, $CDCl_3$), δ, ppm: 162.1, 154.5, 149.9, 135.2, 130.0, 128.9, 127.0, 126.6, 125.3, 113.7, 72.9, 34.0, 24.0; ESI-HRMS, *m/z*: Calcd for $C_{23}H_{24}N_2ONa$ [M+Na]$^+$: 367.1786, Found: 367.1781.

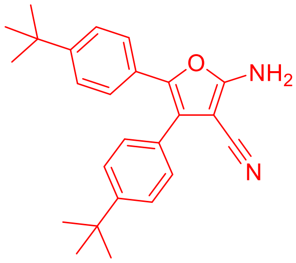

2-Amino-4,5-bis(4-(*tert*-butyl)phenyl)furan-3-carbonitrile (**3e**): White solid (81.2 mg, yield 73%), m.p. 178.4-180.4 °C; ^{1}H NMR (600 MHz, $CDCl_3$), δ, ppm: 7.42 (*d*, 2H, *J* = 8.4 Hz, ArH), 7.39 (*d*, 2H, *J* = 8.4 Hz, ArH), 7.34 (*d*, 2H, *J* = 8.4 Hz, ArH), 7.27 (*d*, 2H, *J* = 8.4 Hz, ArH), 5.03 (*s*, 2H, NH_2), 1.36 (*s*, 9H, $3CH_3$), 1.30 (*s*, 9H, $3CH_3$); ^{13}C NMR (150 MHz, $CDCl_3$), δ, ppm: 161.9, 151.2, 150.5, 139.7, 128.7, 127.0, 125.9, 125.5, 125.0, 121.0, 115.6, 73.3, 34.8, 31.4; ESI-HRMS, *m/z*: Calcd for $C_{25}H_{29}N_2O$ [M+H]$^+$: 373.2274, Found: 373.2272.

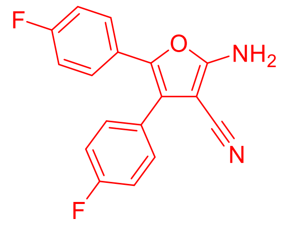

2-Amino-4,5-bis(4-fluorophenyl)furan-3-carbonitrile (**3f**): White solid (77.3 mg, yield 87%), m.p. 210.3-211.9 ºC (210.0-212.0 ºC[1]); ^{1}H NMR (600 MHz, DMSO-d_6), δ, ppm: 7.73 (*s*, 2H, NH_2), 7.44-7.40 (*m*, 2H, ArH), 7.33-7.29 (*m*, 2H, ArH), 7.25-7.22 (*m*, 2H, ArH), 7.16-7.12 (*m*, 2H, ArH).

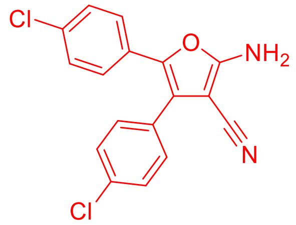

2-Amino-4,5-bis(4-chlorophenyl)furan-3-carbonitrile (**3g**): White solid (84.0 mg, yield 85%), m.p. 222.7-224.6 ºC (223.0-225.0 ºC[1]); ^{1}H NMR (600 MHz, DMSO-d_6), δ, ppm: 7.81 (*s*, 2H, NH_2), 7.54 (*d*, 2H, J = 8.4 Hz, ArH), 7.41 (*d*, 2H, J = 8.4 Hz, ArH), 7.37 (*d*, 2H, J = 8.4 Hz, ArH), 7.21 (*d*, 2H, J = 8.4 Hz, ArH).

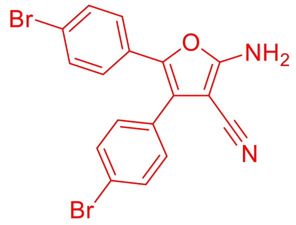

2-Amino-4,5-bis(4-bromophenyl)furan-3-carbonitrile(**3h**): White solid (100.9 mg, yield 81%), m.p. 225.1-226.7 ºC (226.0-227.0 ºC[1]); ^{1}H NMR (600 MHz, DMSO-d_6), δ, ppm: 7.82 (*s*, 2H, NH_2), 7.67 (*d*, 2H, J = 8.4 Hz, ArH), 7.49 (*d*, 2H, J = 8.4 Hz, ArH), 7.34 (*d*, 2H, J = 8.4 Hz, ArH), 7.14 (*d*, 2H, J = 8.4 Hz, ArH).

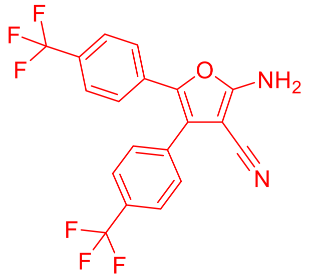

2-Amino-4,5-bis(4-bromophenyl)furan-3-carbonitrile (**3i**): White solid (113.2 mg, yield 95%), m.p. 254.7-256.5 ºC; ^{1}H NMR (600 MHz, DMSO-*d*$_6$), *δ*, ppm: 8.00 (*s*, 2H, NH_2), 7.87 (*d*, 2H, *J* = 7.8 Hz, ArH), 7.66 (*d*, 2H, *J* = 8.4 Hz, ArH), 7.65 (*d*, 2H, *J* = 8.4 Hz, ArH), 7.38 (*d*, 2H, *J* = 7.8 Hz, ArH); ^{13}C NMR (150 MHz, DMSO-*d*$_6$), *δ*, ppm: 164.5, 136.2, 135.6, 133.2, 130.3, 129.5 (*q*, *J* = 32.3 Hz), 127.4 (*q*, *J* = 31.5 Hz), 126.6 (*q*, *J* = 4.1 Hz), 126.4 (*q*, *J* = 267.8 Hz), 126.3 (*q*, *J* = 4.2 Hz), 124.6 (*q*, *J* = 269.3 Hz), 123.7, 121.9, 115.4, 70.1; ^{19}F NMR (564 MHz, DMSO-*d*$_6$), *δ*, ppm: -61.178, -61.811; ESI-HRMS, *m/z*: Calcd for $C_{19}H_9F_6N_2O$ $[M-H]^-$: 395.0625, Found: 395.0626.

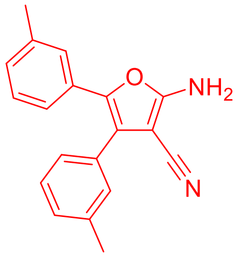

2-Amino-4,5-di-*m*-tolylfuran-3-carbonitrile (**3j**): White solid (66.6 mg, yield 77%), m.p. 157.7-159.4 ºC; ^{1}H NMR (600 MHz, $CDCl_3$), *δ*, ppm: 7.31-7.26 (*m*, 2H, ArH), 7.24 (*s*, 1H, ArH), 7.23 (*s*, 1H, ArH), 7.18 (*d*, *J* = 7.8 Hz, 1H, ArH), 7.14-7.09 (*m*, 2H, ArH), 7.02 (*d*, *J* = 7.2 Hz, 1H, ArH), 5.04 (*s*, 2H, NH_2), 2.36 (*s*, 3H, CH_3), 2.27 (*s*, 3H, CH_3); ^{13}C NMR (150 MHz, $CDCl_3$), *δ*, ppm: 161.9, 139.7, 138.6, 138.2, 131.1, 129.7, 129.2, 128.4, 126.2, 125.9, 122.5, 121.8, 115.3, 73.4, 21.6, 21.5； ESI-HRMS, *m/z*: Calcd for $C_{19}H_{17}N_2O$ $[M+H]^+$: 289.1335, Found: 289.1331.

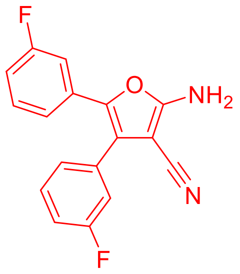

2-Amino-4,5-bis(3-fluorophenyl)furan-3-carbonitrile (**3k**): White solid (74.6 mg, yield 84%),

m.p. 201.3-202.9 ºC (201.0-202.0 ºC[1]); ^{1}H NMR (600 MHz, DMSO-d_6), δ, ppm: 7.87 (*s*, 2H, NH_2), 7.56-7.51 (*m*, 1H, ArH), 7.34-7.27 (*m*, 2H, ArH), 7.24 (*d*, J = 7.2 Hz, 2H, ArH), 7.04-7.00 (*m*, 2H, ArH), 6.94 (*d*, J = 8.4 Hz, 1H, ArH).

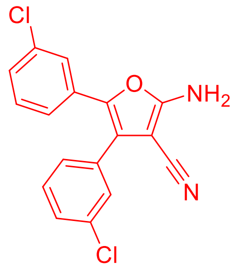

2-Amino-4,5-bis(3-chlorophenyl)furan-3-carbonitrile (**3l**): White solid (80.4, yield 82%), m.p. 154.7-156.4 ºC (154.0-156.0 ºC[1]); ^{1}H NMR (600 MHz, $CDCl_3$), δ, ppm: 7.41 (*s*, 1H, ArH), 7.40-7.35 (*m*, 3H, ArH), 7.32-7.30 (*m*, 1H, ArH), 7.20-7.17 (*m*, 1H, ArH), 7.16-7.12 (*m*, 2H, ArH), 5.13 (*s*, 2H, NH_2).

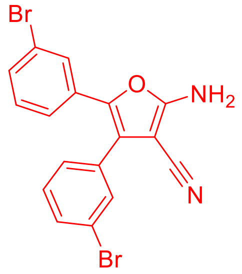

2-Amino-4,5-bis(3-bromophenyl)furan-3-carbonitrile (**3m**): White solid (94.8 mg, yield 76%), m.p. 185.4-187.0 ºC; ^{1}H NMR (600 MHz, DMSO-d_6), δ, ppm: 7.89 (*s*, 2H, NH_2), 7.65 (*d*, 2H, J = 8.4 Hz, ArH), 7.59 (*s*, 1H, ArH), 7.46-7.43 (*m*, 1H, ArH), 7.41 (*d*, J = 7.8 Hz, 1H, ArH), 7.38-7.35 (*m*, 2H, ArH), 7.24-7.20 (*m*, 1H, ArH), 7.14 (*d*, J = 8.4 Hz, 1H, ArH); ^{13}C NMR (150 MHz, DMSO-d_6), δ, ppm: 164.2, 135.7, 133.6, 132.0, 131.8, 131.3, 130.1, 128.5, 127.0, 123.3, 122.6, 115.6, 69.8; ESI-HRMS, *m/z*: Calcd for $C_{17}H_{11}N_2OBr_2$ $[M+H]^+$: 416.9238, Found: 416.9237.

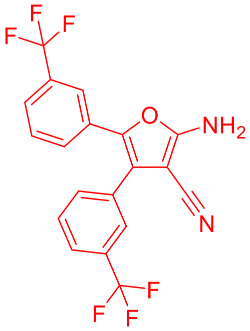

2-Amino-4,5-bis(3-(trifluoromethyl)phenyl)furan-3-carbonitrile (**3n**): White solid (95.0, yield 80%), m.p. 253.7-255.4 ºC; ^{1}H NMR (600 MHz, $CDCl_3$), *δ*, ppm: 7.70-7.67 (*m*, 3H, ArH), 7.65 (*d*, *J* = 7.8 Hz, 1H, ArH), 7.61 (*s*, 1H, ArH), 7.60-7.57 (*m*, 1H, ArH), 7.46 (*d*, *J* = 7.2 Hz, 1H, ArH), 7.42 (*d*, *J* = 7.8 Hz, 1H, ArH), 7.34 (*t*, *J* = 7.8 Hz, 1H, ArH), 5.27 (*s*, 2H, NH_2); ^{13}C NMR (150 MHz, $CDCl_3$), *δ*, ppm: 162.3, 138.4, 132.3, 131.5 (*q*, *J* = 247.4 Hz), 131.4 (*q*, *J* = 243.0 Hz), 129.8, 129.1, 127.9, 125.7 (*q*, *J* = 43.4 Hz), 125.6 (*q*, *J* = 42.5 Hz), 124.6, 124.2 (*q*, *J* = 3.9 Hz), 122.8, 121.8 (*q*, *J* = 4.5 Hz), 114.4, 72.9; ^{19}F NMR (564 MHz, $CDCl_3$), *δ*, ppm: -62.910, -63.205; ESI-HRMS, *m/z*: Calcd for $C_{19}H_9F_6N_2O$ $[M-H]^-$: 395.0625, Found: 395.0622.

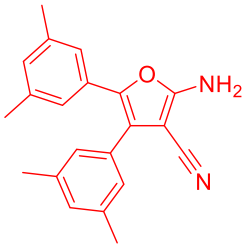

2-Amino-4,5-bis(4-bromophenyl)furan-3-carbonitrile (**3o**): White solid (61.6 mg, yield 65%), m.p. 170.2-172.1 ºC; ^{1}H NMR (600 MHz, DMSO-d_6), *δ*, ppm: 7.64 (*s*, 2H, NH_2), 7.03 (*s*, 1H, ArH), 6.99 (*s*, 2H, ArH), 6.89 (*s*, 2H, ArH), 6.80 (*s*, 1H, ArH), 2.26 (*s*, 6H, CH_3), 2.12 (*s*, 6H, CH_3); ^{13}C NMR (150 MHz, DMSO-d_6), *δ*, ppm: 164.0, 138.3, 137.3, 131.6, 130.2, 128.9, 127.0, 123.7, 122.5, 116.2, 69.6, 21.4, 21.3; ESI-HRMS, *m/z*: Calcd for $C_{21}H_{21}N_2O$ $[M+H]^+$: 317.1648, Found: 317.1644.

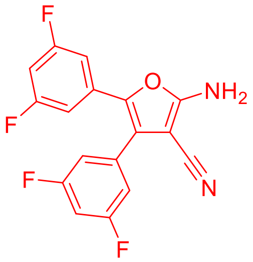

2-Amino-4,5-bis(3,5-difluorophenyl)furan-3-carbonitrile (**3p**): White solid (70.9, yield 71%), m.p. 228.3-230.2 ºC; ^{1}H NMR (600 MHz, DMSO-d_6), δ, ppm: 8.00 (*s*, 2H, NH_2), 7.39 (*s*, 1H, ArH), 7.18 (*s*, 2H, ArH), 7.09 (*s*, 1H, ArH), 6.79 (*s*, 2H, ArH); ^{13}C NMR (150 MHz, DMSO-d_6), δ, ppm: 164.1, 163.2 (*d*, *J* = 246.3 Hz), 163.1 (*d*, *J* = 246.9 Hz), 163.0 (*d*, *J* = 244.4 Hz), 162.9 (*d*, *J* = 244.1 Hz), 135.2 (*d*, *J* = 3.5 Hz), 134.4, 132.3, 123.0 (*d*, *J* = 3.2 Hz), 115.1, 112.9 (*d*, *J* = 25.4 Hz), 107.3 (*d*, *J* = 27.5 Hz), 105.2 (*d*, *J* = 25.7 Hz), 105.0 (*d*, *J* = 25.4 Hz), 103.0 (*d*, *J* = 26.0 Hz), 102.8 (*d*, *J* = 26.0 Hz), 70.1; ^{19}F NMR (564 MHz, DMSO-d_6), δ, ppm: -108.408, -108.771; ESI-HRMS, *m/z*: Calcd for $C_{17}H_7F_4N_2O$ $[M-H]^-$: 331.0500, Found: 331.0499.

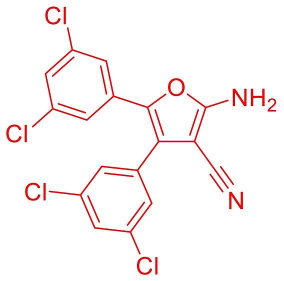

2-Amino-4,5-bis(3,5-dichlorophenyl)furan-3-carbonitrile (**3q**): White solid (80.8, yield 68%), m.p. 243.6-245.5 ºC; ^{1}H NMR (600 MHz, DMSO-d_6), δ, ppm: 8.03 (*s*, 2H, NH_2), 7.74 (*s*, 1H, ArH), 7.50 (*s*, 2H, ArH), 7.41 (*s*, 1H, ArH), 7.11 (*s*, 2H, ArH); ^{13}C NMR (150 MHz, DMSO-d_6), δ, ppm: 164.3, 135.3, 135.0, 134.7, 134.3, 132.3, 129.1, 128.1, 126.6, 122.6, 115.1, 70.0; ESI-HRMS, *m/z*: Calcd for $C_{17}H_8N_2ONaCl_4$ $[M+Na]^+$: 418.9288, Found: 418.9286.

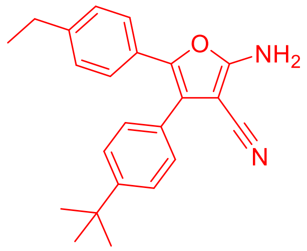

2-Amino-4-(4-(*tert*-butyl)phenyl)-5-(4-ethylphenyl)furan-3-carbonitrile (**3r**): White solid (72.2 mg, yield 70%), m.p. 188.0-190.0 ºC; ^{1}H NMR (600 MHz, $CDCl_3$), δ, ppm: 7.43-7.40 (*m*, 1H, ArH), 7.39-7.35 (*m*, 2H, ArH), 7.33-7.30 (*m*, 2H, ArH), 7.28-7.26 (*m*, 1H, ArH), 7.25-7.22 (*m*, 1H, ArH), 7.10-7.07 (*m*, 1H, ArH), 5.00 (*s*, 2H, NH_2), 2.72-2.59 (*m*, 2H, CH_2), 1.36 (*s*, 3H, CH_3), 1.31-1.27 (*m*, 9H, $3CH_3$); ^{13}C NMR (150 MHz, $CDCl_3$), δ, ppm: 162.0, 156.9, 151.2, 150.5, 144.4, 139.6, 129.8,

129.0, 128.0, 125.5, 125.0, 113.7, 73.2, 35.3, 31.3, 28.8, 15.4, ESI-HRMS, *m/z*: Calcd for $C_{23}H_{25}N_2O$ $[M+H]^+$: 345.1961, Found: 345.1958.

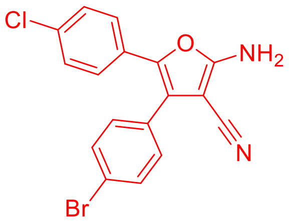

2-Amino-4-(4-bromophenyl)-5-(4-chlorophenyl)furan-3-carbonitrile (**3s**): White solid (80.6 mg, yield 72%), m.p. 235.2-236.9 ºC; ^{1}H NMR (600 MHz, DMSO-d_6), δ, ppm: 7.83 (*s*, 2H, NH_2), 7.68 (*d*, 1H, *J* = 8.4 Hz, ArH), 7.54 (*d*, 1H, *J* = 9.0 Hz, ArH), 7.50 (*d*, 1H, *J* = 7.2 Hz, ArH), 7.41 (*d*, 1H, *J* = 8.4 Hz, ArH), 7.37 (*d*, 1H, *J* = 7.2 Hz, ArH), 7.34 (*d*, 1H, *J* = 8.4 Hz, ArH), 7.23-7.20 (*m*, 1H, ArH), 7.16-7.13 (*m*, 1H, ArH); ^{13}C NMR (150 MHz, DMSO-d_6), δ, ppm: 164.2, 136.3, 133.8, 132.7, 131.4, 130.7, 129.7, 128.8, 126.7, 122.4, 120.5, 115.7, 69.6; ESI-HRMS, *m/z*: Calcd for $C_{17}H_9BrClN_2O$ $[M-H]^-$: 370.9592, Found: 370.9593.

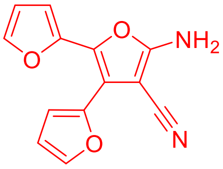

5'-Amino-[2,2':3',2''-terfuran]-4'-carbonitrile (**3t**): Brown solid (48.2 mg, yield 67%), m.p. 186.2-187.9 ºC (187.0-188.0 ºC[3]); ^{1}H NMR (600 MHz, $CDCl_3$), δ, ppm: 7.86 (*s*, 2H, NH_2), 7.81 (*d*, *J* = 2.4 Hz, 1H, ArH), 7.75 (*d*, *J* = 2.4 Hz, 1H, ArH), 6.85 (*d*, *J* = 2.4 Hz, 1H, ArH), 6.74 (*d*, *J* = 2.4 Hz, 1H, ArH), 6.64-6.63 (*m*, 1H, ArH), 6.62-6.60 (*m*, 1H, ArH).

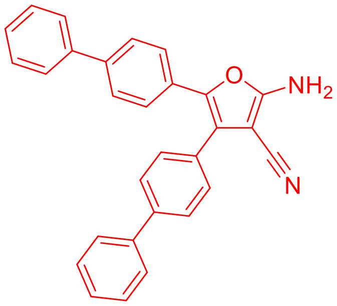

4,5-Bi([1,1'-biphenyl]-4-yl)-2-aminofuran-3-carbonitrile (**3u**): White solid (119.9 mg, yield

97%), m.p. 237.1-239.1 ºC; ^{1}H NMR (600 MHz, DMSO-d_6), δ, ppm: 7.80 (*d*, J = 8.4 Hz, 2H, ArH), 7.79 (*s*, 2H, NH_2), 7.75 (*d*, J = 8.4 Hz, 2H, ArH), 7.63 (*d*, J = 7.2 Hz, 2H, ArH), 7.61 (*d*, J = 8.4 Hz, 2H, ArH), 7.52 (*d*, J = 8.4 Hz, 2H, ArH), 7.51-7.48 (*m*, 2H, ArH), 7.44-7.41 (*m*, 3H, ArH), 7.38 (*d*, J = 8.4 Hz, 2H, ArH), 7.33 (*t*, J = 7.2 Hz, 1H, ArH); ^{13}C NMR (150 MHz, DMSO-d_6), δ, ppm: 164.2, 140.4, 139.8, 138.8, 137.1, 130.8, 130.0, 129.5, 129.0, 128.3, 127.7, 127.3, 127.1, 126.8, 125.2, 122.2, 116.1, 69.8; ESI-HRMS, *m/z*: Calcd for $C_{29}H_{21}N_2O$ $[M+H]^+$: 413.1648, Found: 413.1643.

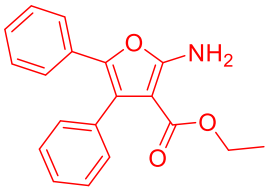

1-(2-Amino-4,5-diphenylfuran-3-yl)propan-1-one (**3v**): White solid (43.3 mg, yield 47%), m.p. 161.2-163.0 ºC (163.0-165.0 ºC[4]); ^{1}H NMR (600 MHz, DMSO-d_6), δ, ppm: 7.40-7.34 (*m*, 3H, ArH), 7.29-7.28 (*m*, 2H, ArH), 7.27 (*s*, 2H, NH_2), 7.20-7.16 (*m*, 2H, ArH), 7.11-7.07 (*m*, 3H, ArH), 3.92 (*q*, J = 7.2 Hz, 2H, OCH_2), 0.91 (*t*, J = 7.2 Hz, 3H, CH_3).

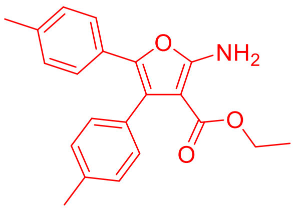

Ethyl 2-amino-4,5-di-*p*-tolylfuran-3-carboxylate (**3w**): White solid (37.2 mg, yield 37%), m.p. 147.0-148.8 ºC; ^{1}H NMR (600 MHz, $CDCl_3$), δ, ppm: 7.21 (*d*, J = 7.8 Hz, 2H, ArH), 7.16 (*d*, J = 7.8 Hz, 4H, ArH), 7.15 (*d*, J = 7.2 Hz, 2H, ArH), 6.98 (*d*, J = 7.2 Hz, 2H, ArH), 4.06 (*q*, J = 7.2 Hz, 2H, OCH_2), 2.39 (*s*, 3H, CH_3), 2.26 (*s*, 3H, CH_3), 1.03 (*t*, J = 7.2 Hz, 3H, CH_3); ^{13}C NMR (150 MHz, $CDCl_3$), δ, ppm: 163.5, 158.9, 140.7, 136.8, 130.1, 129.9, 129.4, 128.9, 128.7, 124.7, 128.3, 89.2, 59.2, 21.4, 14.0; ESI-HRMS, *m/z*: Calcd for $C_{21}H_{22}NO_3$ $[M+H]^+$: 336.1594, Found: 336.1589.

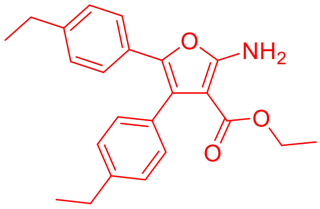

Ethyl 2-amino-4,5-bis(4-ethylphenyl)furan-3-carboxylate (**3x**): White solid (37.0 mg, yield 34%), m.p. 157.0-159.7 ºC; ^{1}H NMR (600 MHz, $CDCl_3$), *δ*, ppm: 7.24 (*d*, *J* = 8.4 Hz, 2H, ArH), 7.19 (*d*, *J* = 8.4 Hz, 4H, ArH), 7.02 (*d*, *J* = 8.4 Hz, 2H, ArH), 4.03 (*q*, *J* = 7.2 Hz, 2H, OCH_2), 2.70 (*q*, *J* = 7.8 Hz, 2H, CH_2), 2.60 (*q*, *J* = 7.2 Hz, 2H, CH_2), 1.27 (*t*, *J* = 7.8 Hz, 3H, CH_3), 1.18 (*t*, *J* = 7.8 Hz, 3H, CH_3), 0.99 (*t*, *J* = 7.2 Hz, 3H, CH_3); ^{13}C NMR (150 MHz, $CDCl_3$), *δ*, ppm: 163.6, 162.6, 146.8, 145.0, 134.7, 130.3, 128.9, 128.2, 127.7, 127.5, 125.8, 89.3, 61.7, 28.5, 15.0, 13.9; ESI-HRMS, *m/z*: Calcd for $C_{23}H_{26}NO_3$ $[M+H]^+$: 364.1907, Found: 364.1902.

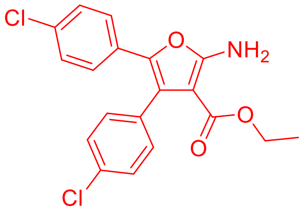

Ethyl 2-amino-4,5-bis(4-chlorophenyl)furan-3-carboxylate (**3y**): White solid (61.9 mg, yield 55%), m.p. 170.0-172.0 ºC; ^{1}H NMR (600 MHz, $CDCl_3$), *δ*, ppm: 7.36 (*d*, *J* = 8.4 Hz, 2H, ArH), 7.26 (*s*, 2H, NH_2), 7.25 (*d*, *J* = 8.4 Hz, 2H, ArH), 7.15 (*d*, *J* = 9.0 Hz, 2H, ArH), 7.12 (*d*, *J* = 9.0 Hz, 2H, ArH), 4.06 (*q*, *J* = 7.2 Hz, 2H, OCH_2), 1.03 (*t*, *J* = 7.2 Hz, 3H, CH_3); ^{13}C NMR (150 MHz, $CDCl_3$), *δ*, ppm: 165.2, 161.7, 138.6, 133.7, 132.0, 131.7, 130.0, 128.7, 128.6, 125.9, 92.2, 59.6, 14.1; ESI-HRMS, *m/z*: Calcd for $C_{19}H_{16}Cl_2NO_3$ $[M+H]^+$: 376.0502, Found: 376.0496.

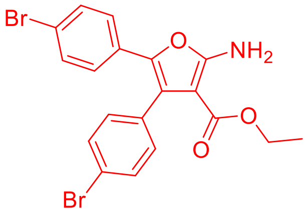

Ethyl 2-amino-4,5-bis(4-bromophenyl)furan-3-carboxylate (**3z**): White solid (72.2 mg, yield 52%), m.p. 155.2-156.8 ºC; ^{1}H NMR (600 MHz, $CDCl_3$), *δ*, ppm: 7.51 (*d*, *J* = 8.4 Hz, 2H, ArH), 7.30 (*d*, *J* = 8.4 Hz, 2H, ArH), 7.19 (*d*, *J* = 8.4 Hz, 2H, ArH), 7.06 (*d*, *J* = 8.4 Hz, 2H, ArH), 5.71 (*b*, 2H,

NH_2), 4.06 (*q*, *J* = 7.2 Hz, 2H, OCH_2), 1.03 (*t*, *J* = 7.2 Hz, 3H, CH_3); ^{13}C NMR (150 MHz, $CDCl_3$), *δ*, ppm: 165.2, 161.7, 138.6, 132.5, 132.0, 131.6, 131.5, 126.2, 121.9, 92.2, 59.6, 14.1; ESI-HRMS, *m/z*: Calcd for $C_{19}H_{16}Br_2NO_3$ $[M+H]^+$: 463.9491, Found: 463.9461.

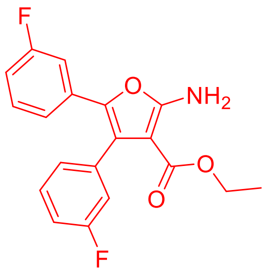

Ethyl 2-amino-4,5-bis(3-fluorophenyl)furan-3-carboxylate (**3aa**): White solid (49.4 mg, yield 48%), m.p. 157.2-158.8 ºC; ^{1}H NMR (600 MHz, $CDCl_3$), *δ*, ppm: 7.37-7.33 (*m*, 1H, ArH), 7.14-7.05 (*m*, 4H, ArH), 6.95-6.91 (*m*, 2H, ArH), 6.82-6.78 (*m*, 1H, ArH), 5.74 (*b*, 2H, NH_2), 4.05 (*q*, *J* = 7.2 Hz, 2H, OCH_2), 1.00 (*t*, *J* = 7.2 Hz, 3H, CH_3); ^{13}C NMR (150 MHz, $CDCl_3$), *δ*, ppm: 165.1, 162.7 (*d*, *J* = 243.2 Hz), 162.6 (*d*, *J* = 244.4 Hz), 161.6, 138.2, 135.5 (*d*, *J* = 8.6 Hz), 132.1 (*d*, *J* = 8.6 Hz), 129.9 (*d*, *J* = 8.6 Hz), 129.7 (*d*, *J* = 8.3 Hz), 125.8 (*d*, *J* = 2.7 Hz), 121.6, 120.0 (*d*, *J* = 2.9 Hz), 117.2 (*d*, *J* = 21.5 Hz), 114.6 (*d*, *J* = 20.1 Hz), 113.3 (*d*, *J* = 21.3 Hz), 111.2 (*d*, *J* = 24.2 Hz), 92.3, 59.4, 13.8; ^{19}F NMR (564 MHz, $CDCl_3$), *δ*, ppm: -112.718, -113.766; ESI-HRMS, *m/z*: Calcd for $C_{19}H_{16}F_2NO_3$ $[M+H]^+$: 344.1093, Found: 344.1089.

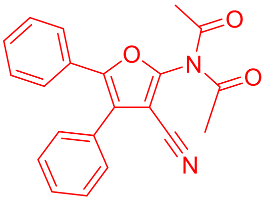

N-Acetyl-*N*-(3-cyano-4,5-diphenylfuran-2-yl)acetamide (**4a**): White solid (66.1 mg, yield 64%), m.p. 157.6-159.5 ºC (156.0-158.0 ºC[3]); ^{1}H NMR (600 MHz, DMSO-d_6), *δ*, ppm: 7.55-7.49 (*m*, 5H, ArH), 7.46-7.43 (*m*, 2H, ArH), 7.40-7.39 (*m*, 3H, ArH), 2.43 (*s*, 6H, $2CH_3$).

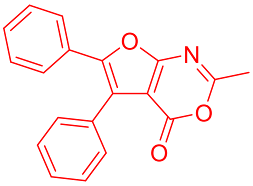

2-Methyl-5,6-diphenyl-4H-furo[2,3-d][1,3]oxazin-4-one (**4b**): White solid (55.4 mg, yield 61%), m.p. 196.7-198.5 ºC (196.0-198.0 ºC[5]); ^{1}H NMR (600 MHz, DMSO-d_6), *δ*, ppm: 7.52- 7.45 (*m*, 3H, ArH), 7.43-7.40 (*m*, 2H, ArH), 7.36-7.29 (*m*, 5H, ArH), 2.16 (*s*, 3H, CH_3).

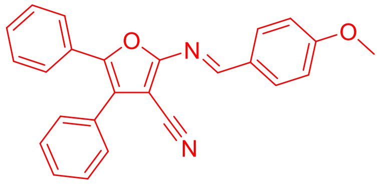

(*E*)-2-((4-Methoxybenzylidene)amino)-4,5-diphenylfuran-3-carbonitrile (**4c**): Yellow solid (76.0 mg, yield 67%), m.p. 168.7-170.5 ℃; ^{1}H NMR (600 MHz, DMSO-d_6), *δ*, ppm: 9.09 (*s*, 1H, CH), 8.04 (*d*, *J* = 9.0 Hz, 2H, ArH), 7.55-7.49 (*m*, 5H, ArH), 7.48-7.47 (*m*, 2H, ArH), 7.41-7.34 (*m*, 3H, ArH), 7.15 (*d*, *J* = 9.0 Hz, 2H, ArH), 3.88 (*s*, 3H, OCH_3); ^{13}C NMR (150 MHz, DMSO-d_6), *δ*, ppm: 164.1, 161.8, 160.2, 145.2, 132.4, 132.3, 130.5, 129.7, 129.5, 129.3, 129.0, 128.3, 126.4, 115.4, 113.9, 92.7, 56.2; ESI-HRMS, *m/z*: Calcd for $C_{25}H_{19}N_2O_2$ $[M+H]^+$: 379.1441, Found: 379.1442.

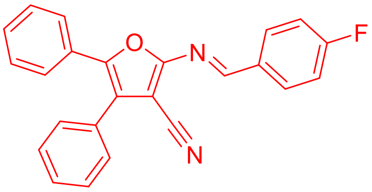

(*E*)-2-((4-Fluorobenzylidene)amino)-4,5-diphenylfuran-3-carbonitrile (**4d**): Yellow solid (77.0 mg, yield 70%), m.p. 173.4-175.3 ℃; ^{1}H NMR (600 MHz, DMSO-d_6), *δ*, ppm: 9.13 (*s*, 1H, CH), 8.19-8.09 (*m*, 2H, ArH), 7.53-7.43 (*m*, 10H, ArH), 7.42-7.33 (*m*, 2H, ArH); ^{13}C NMR (150 MHz, DMSO-d_6), *δ*, ppm: 165.5 (*d*, *J* = 251.4 Hz), 161.1, 159.4, 145.8, 132.7 (*d*, *J* = 9.5 Hz), 132.2, 130.3, 129.7, 129.5, 129.3, 128.8, 126.5, 124.0, 117.0 (*d*, *J* = 22.1 Hz), 113.5, 94.2; ^{19}F NMR (564 MHz, DMSO-d_6), *δ*, ppm: -105.189; ESI-HRMS, *m/z*: Calcd for $C_{24}H_{16}FN_2O$ $[M+H]^+$: 367.1241, Found: 367.1242.

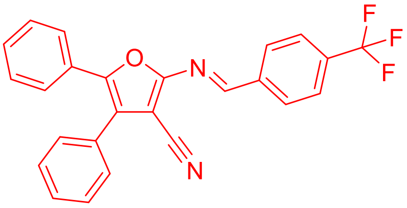

(*E*)-4,5-Diphenyl-2-((4-trifluoromethylbenzylidene)amino)furan-3-carbonitrile (**4e**): Yellow solid (90.0 mg, yield 72%), m.p. 176.7-178.4 ºC; ^{1}H NMR (600 MHz, DMSO-d_6), δ, ppm: 9.22 (*s*, 1H, CH), 8.25 (*d*, J = 8.4 Hz, 2H, ArH), 7.93 (*d*, J = 8.4 Hz, 2H, ArH), 7.55- 7.51 (*m*, 5H, ArH), 7.49-7.47 (*m*, 2H, ArH), 7.41-7.37 (*m*, 3H, ArH); ^{13}C NMR (150 MHz, DMSO-d_6), δ, ppm: 160.7, 158.8, 146.4, 139.0, 132.5 (*q*, J = 32.0 Hz), 130.6, 130.2, 129.7, 129.5, 129.3, 128.7, 126.6, 126.5 (*q*, J = 3.9 Hz), 125.3 (*q*, J = 271.2 Hz), 113.3, 95.8; ^{19}F NMR (564 MHz, DMSO-d_6), δ, ppm: -61.483; ESI-HRMS, *m/z*: Calcd for $C_{25}H_{16}F_3N_2O$ $[M+H]^+$: 417.1209, Found: 417.1205.

NMR Spectra for All Products 3a-3aa, 4a-4e

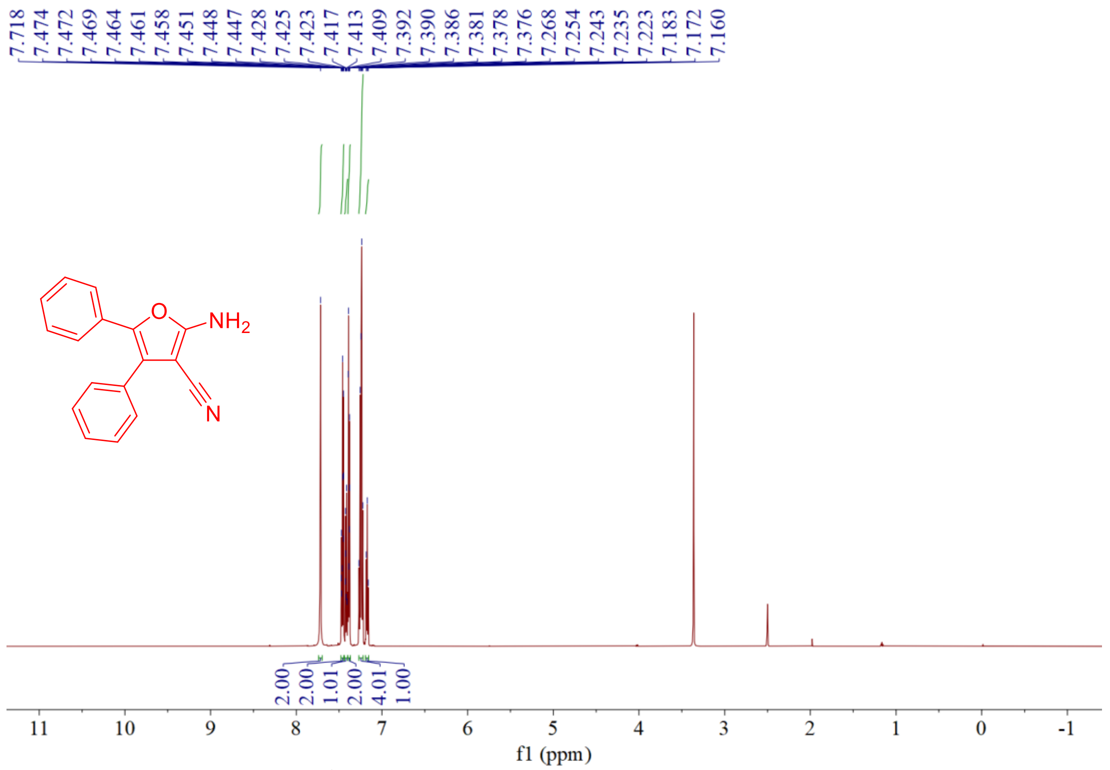

^{1}H NMR spectrum of compound **3a**

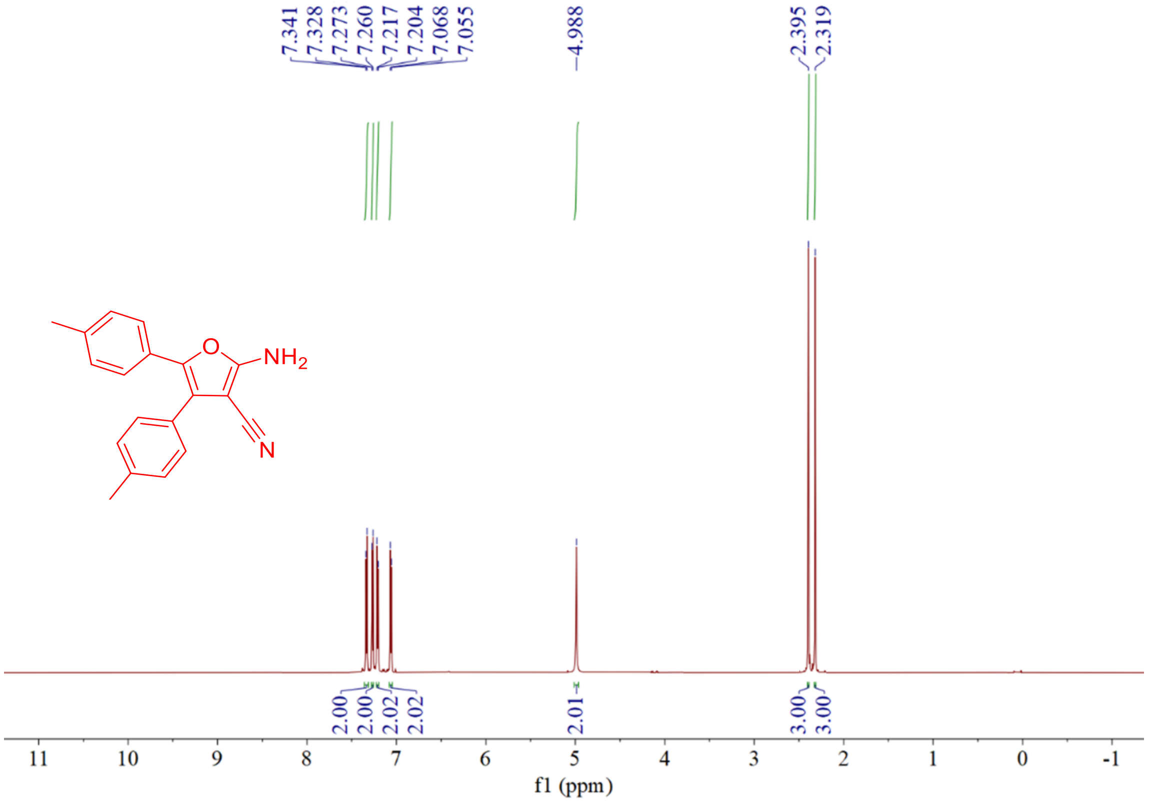

^{1}H NMR spectrum of compound **3b**

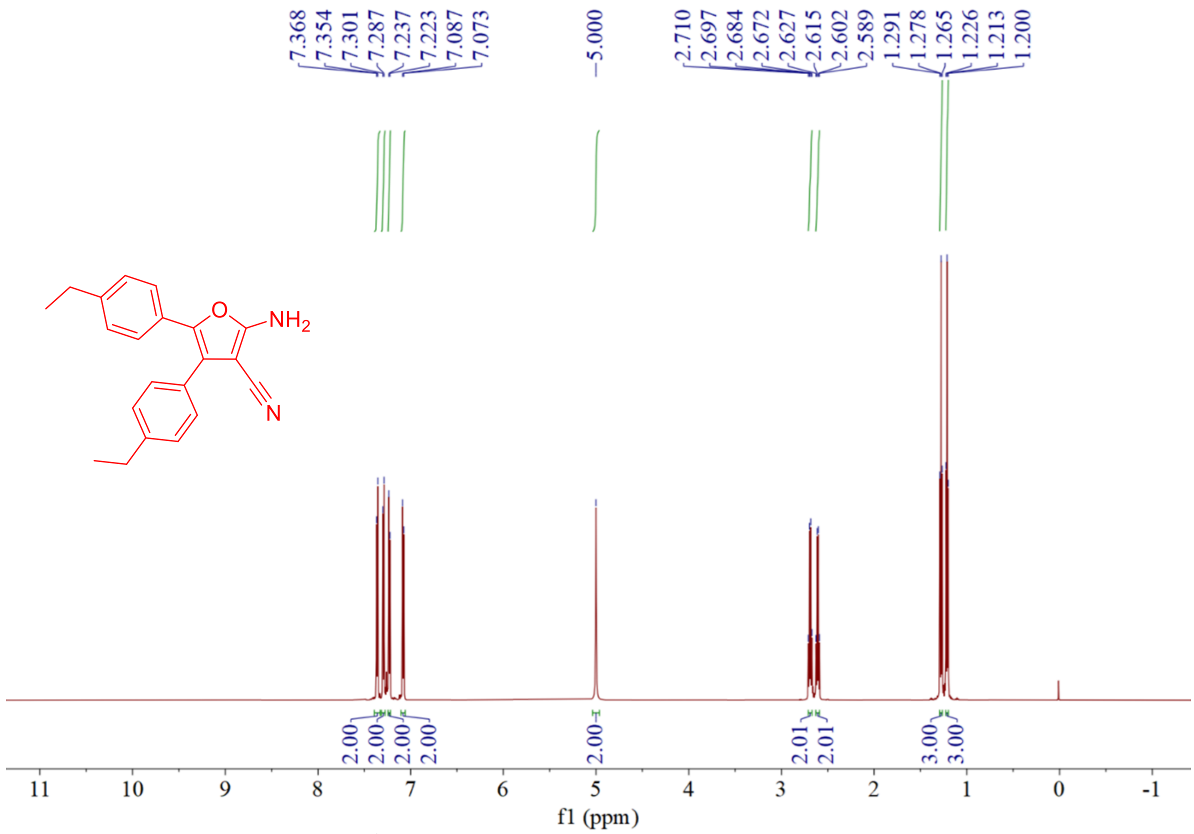

^{1}H NMR spectrum of compound **3c**

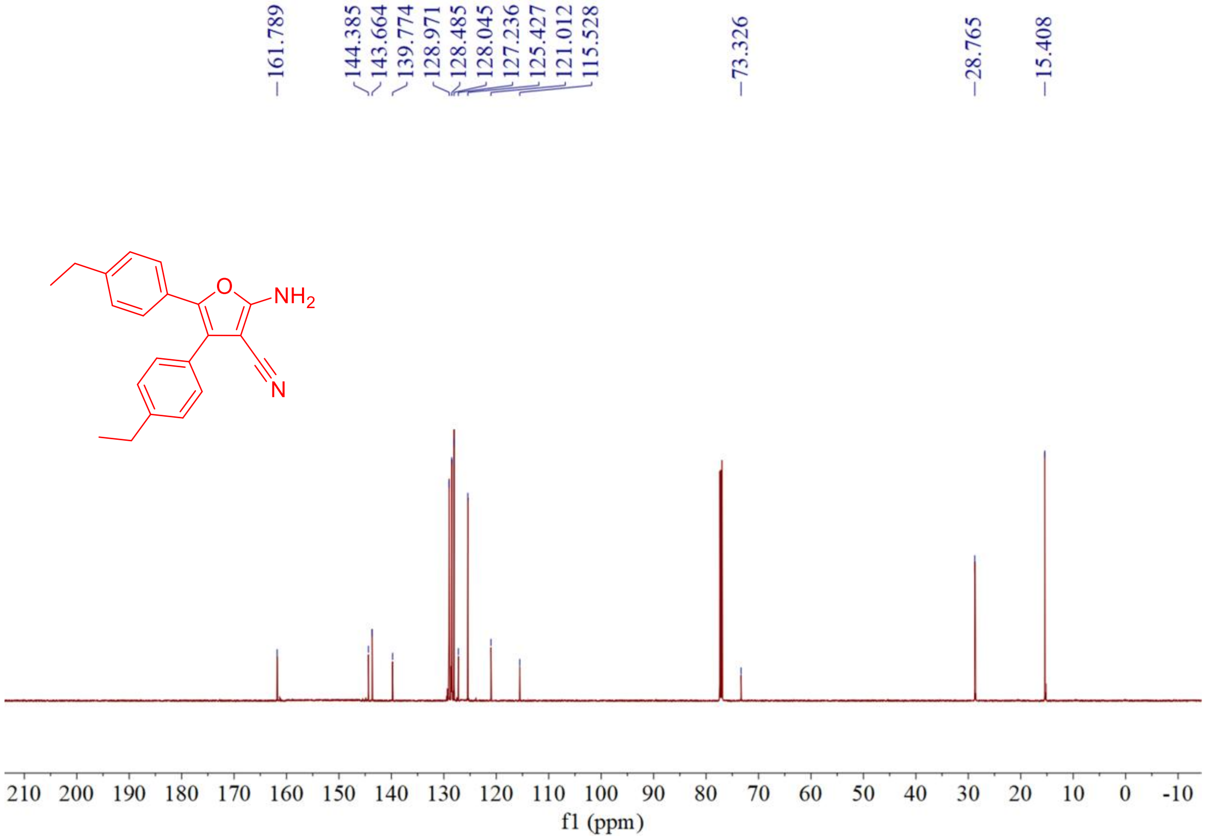

^{13}C NMR spectrum of compound **3c**

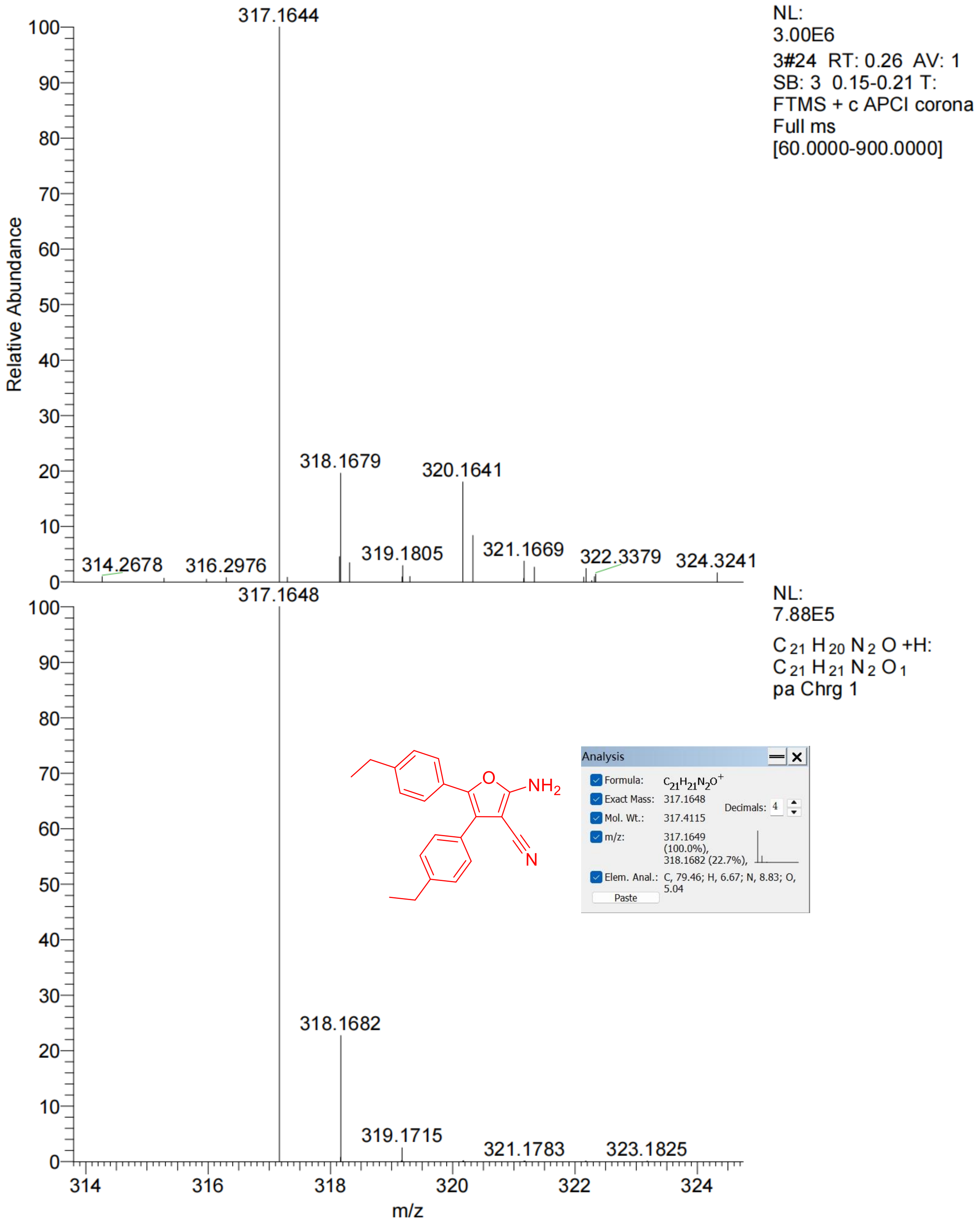

HR-MS spectrum of compound **3c**

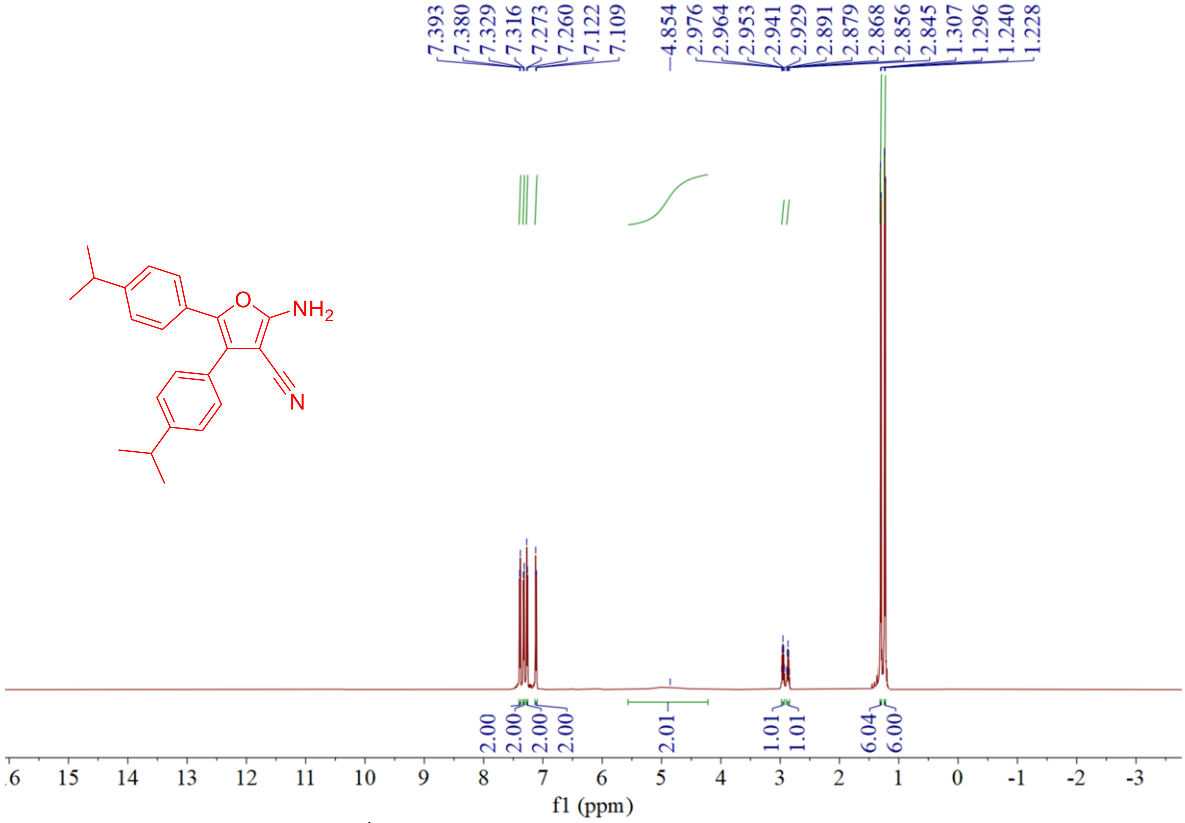

^{1}H NMR spectrum of compound **3d**

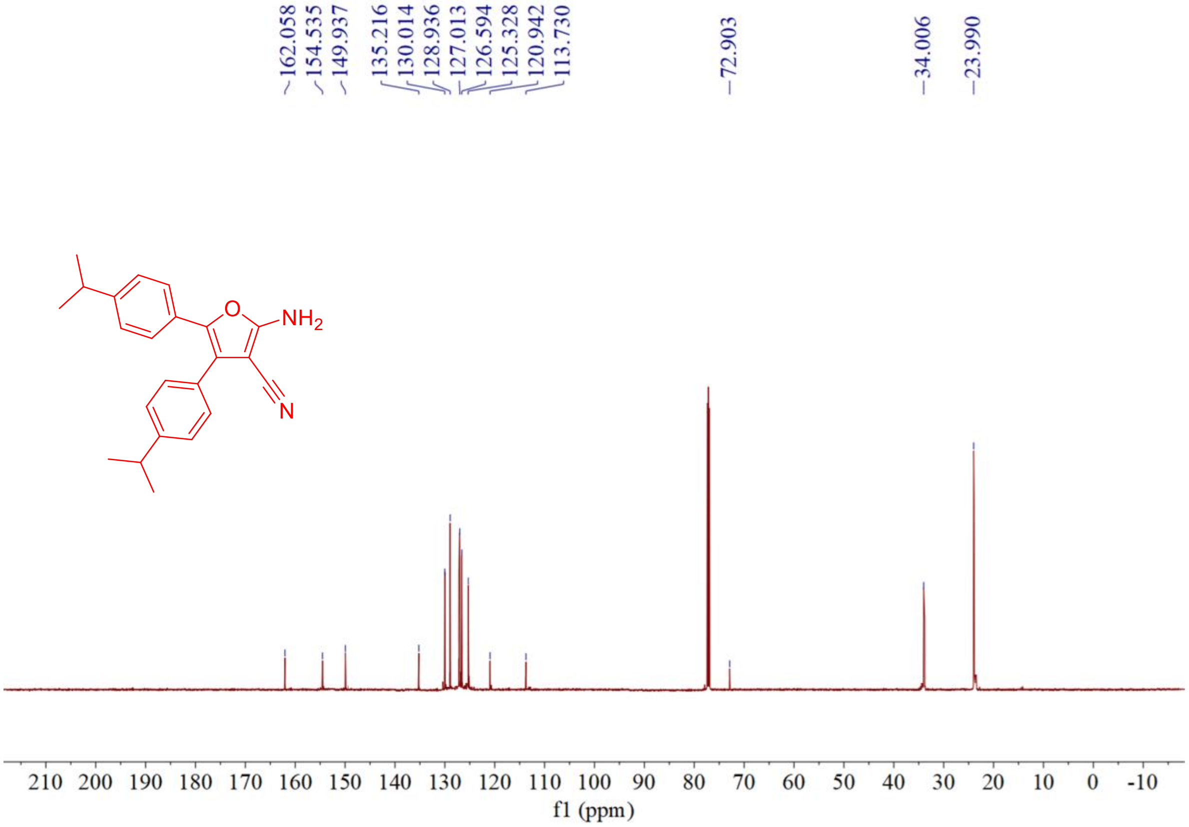

^{13}C NMR spectrum of compound **3d**

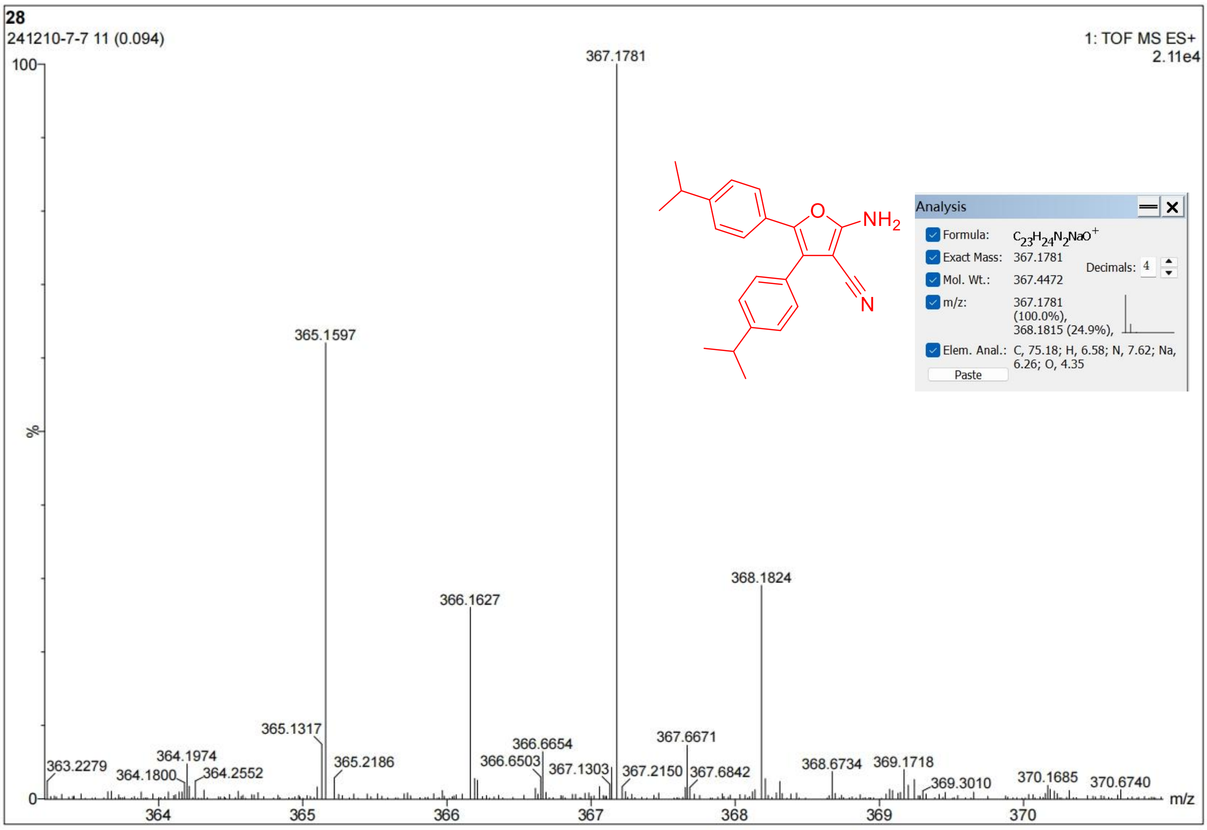

HR-MS spectrum of compound **3d**

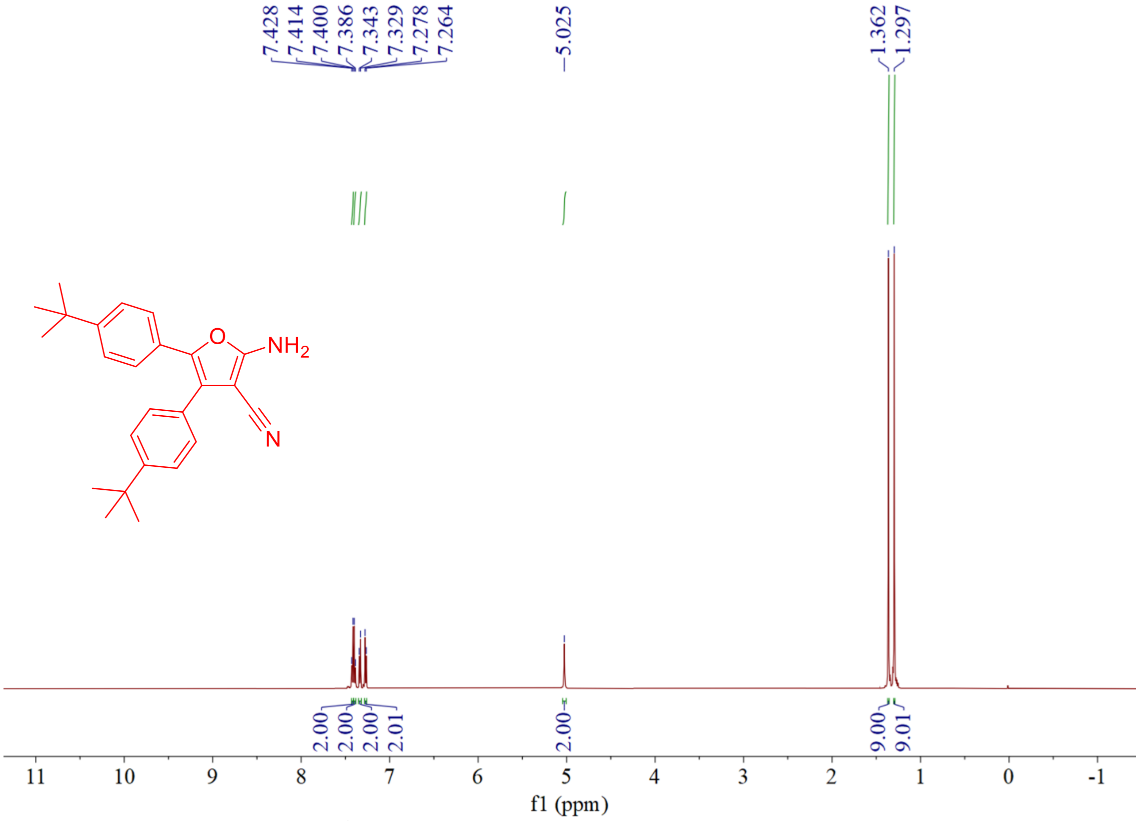

^{1}H NMR spectrum of compound **3e**

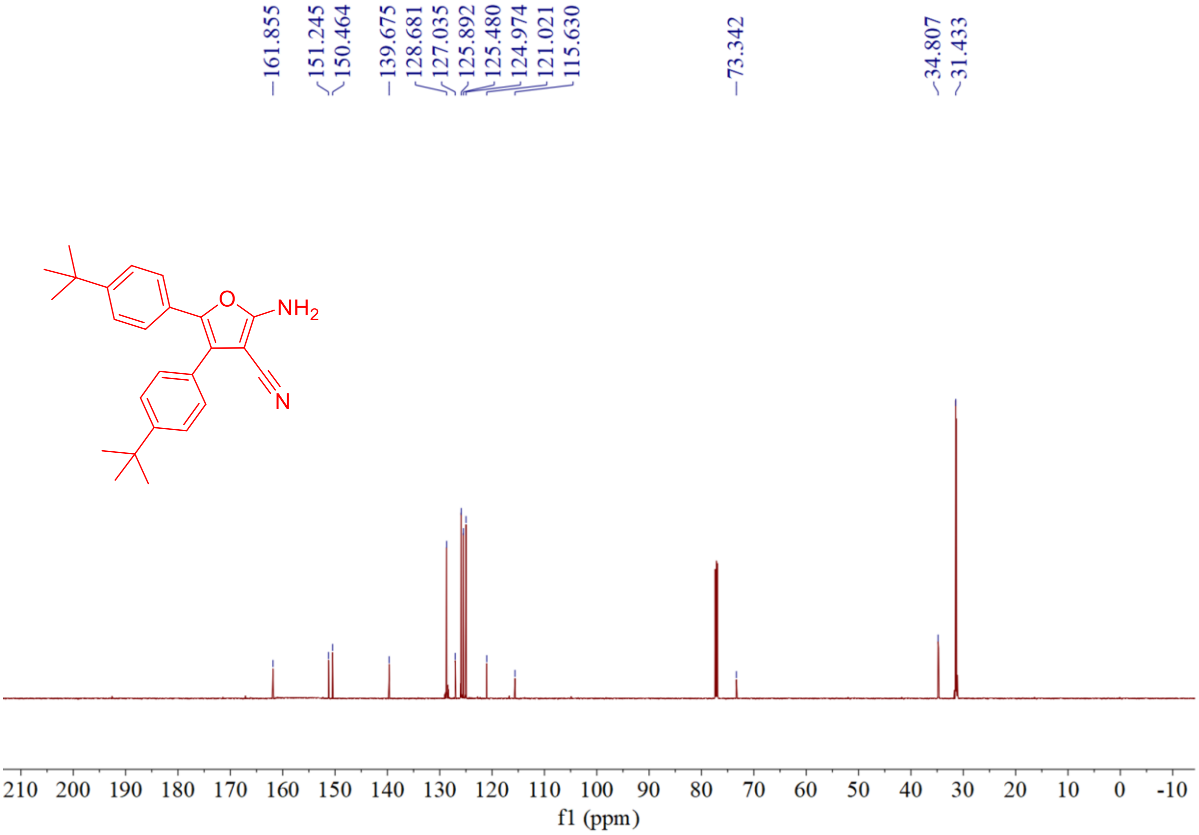

^{13}C NMR spectrum of compound **3e**

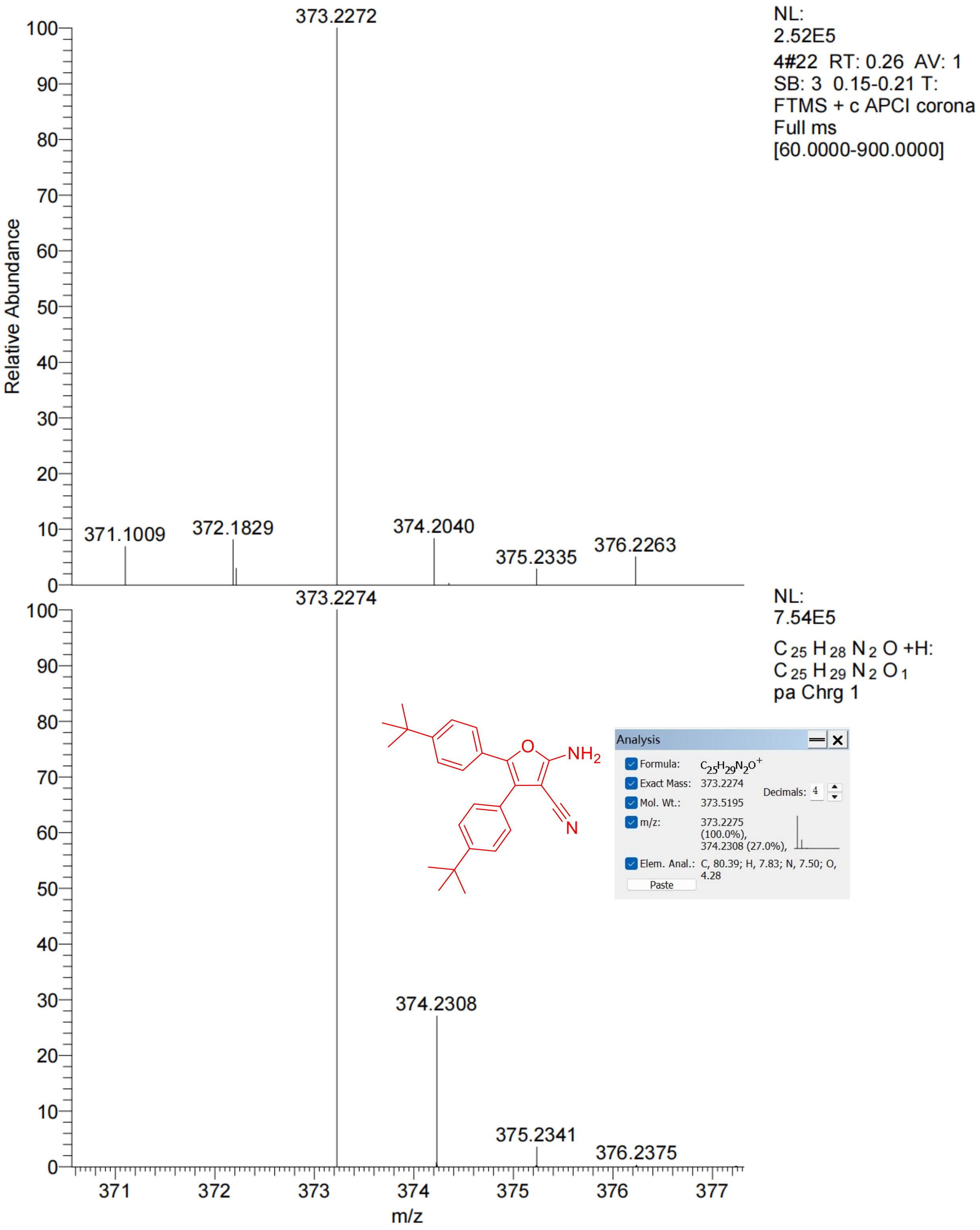

HR-MS spectrum of compound **3e**

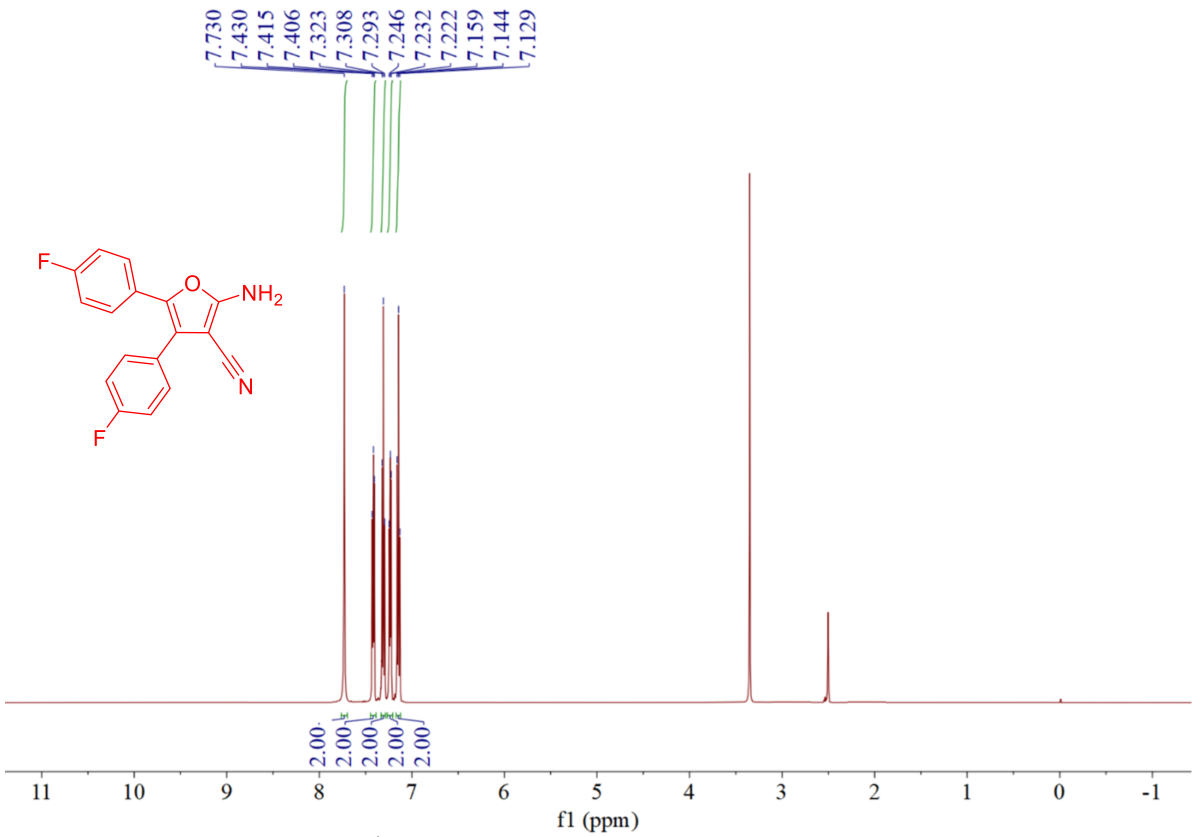

^{1}H NMR spectrum of compound **3f**

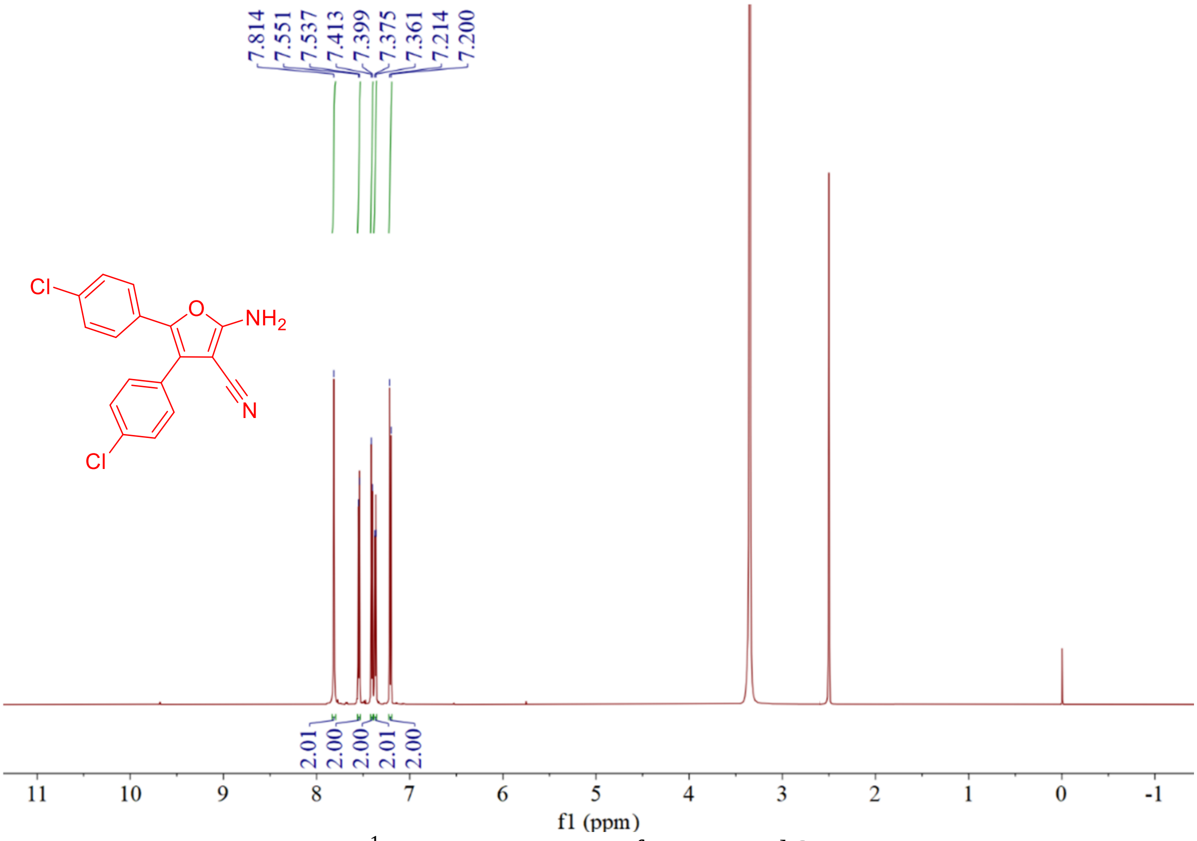

^{1}H NMR spectrum of compound **3g**

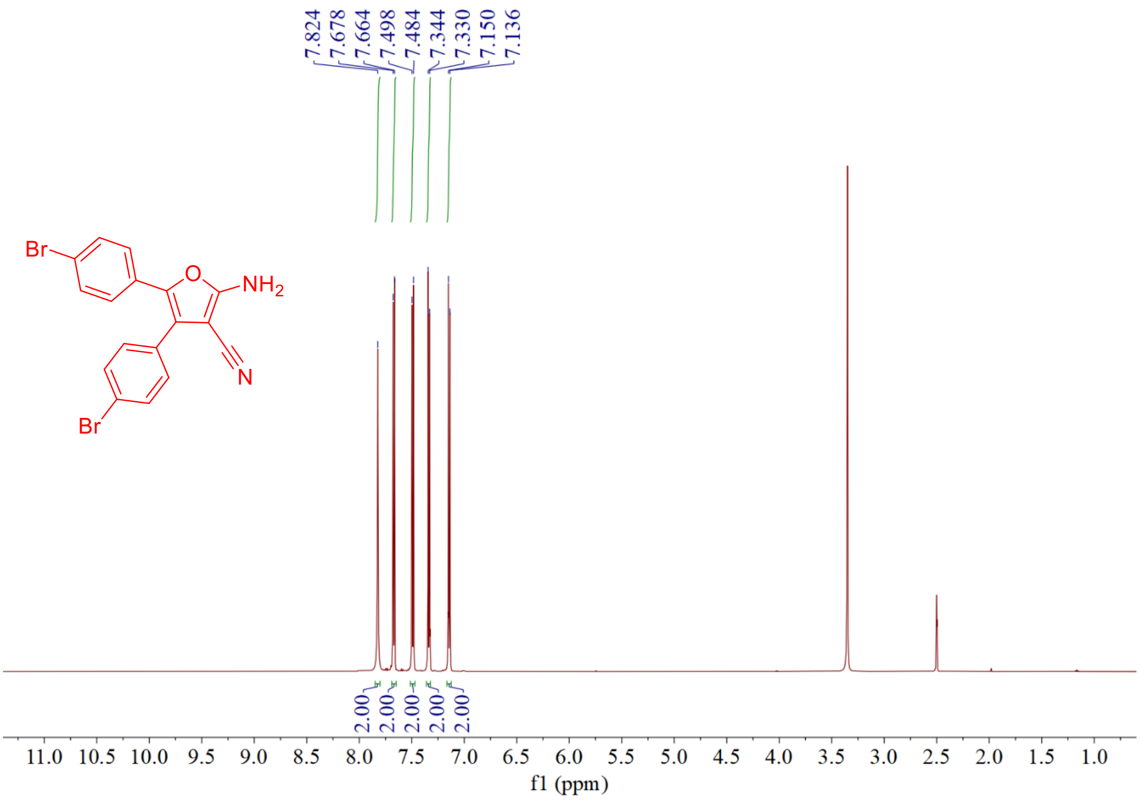

^{1}H NMR spectrum of compound **3h**

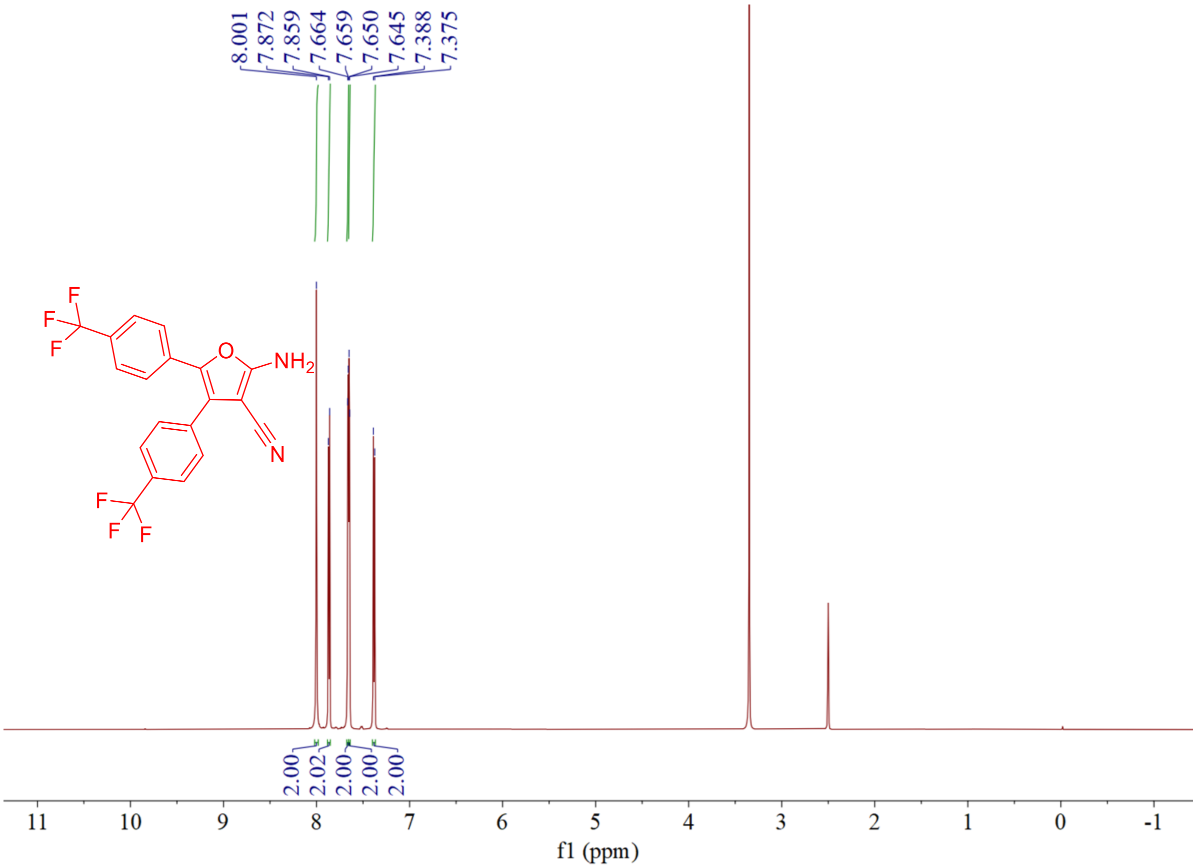

^{1}H NMR spectrum of compound **3i**

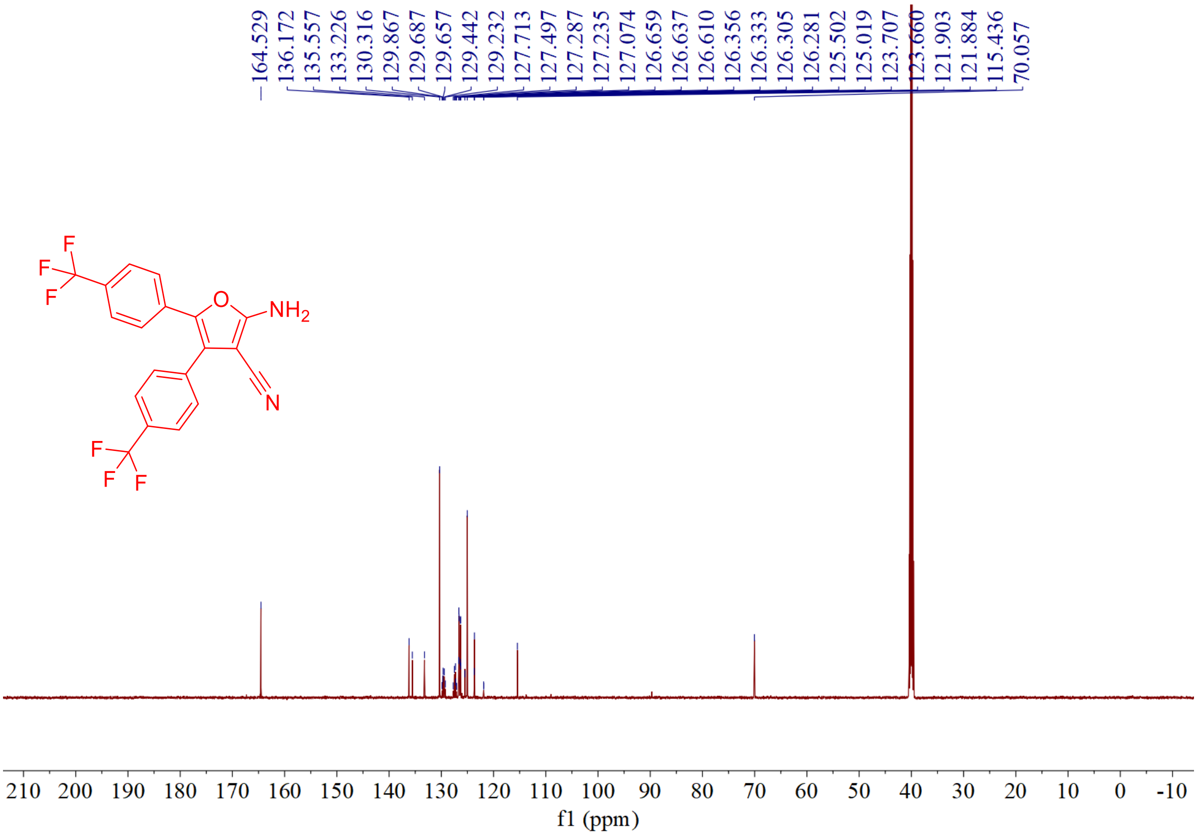

^{13}C NMR spectrum of compound **3i**

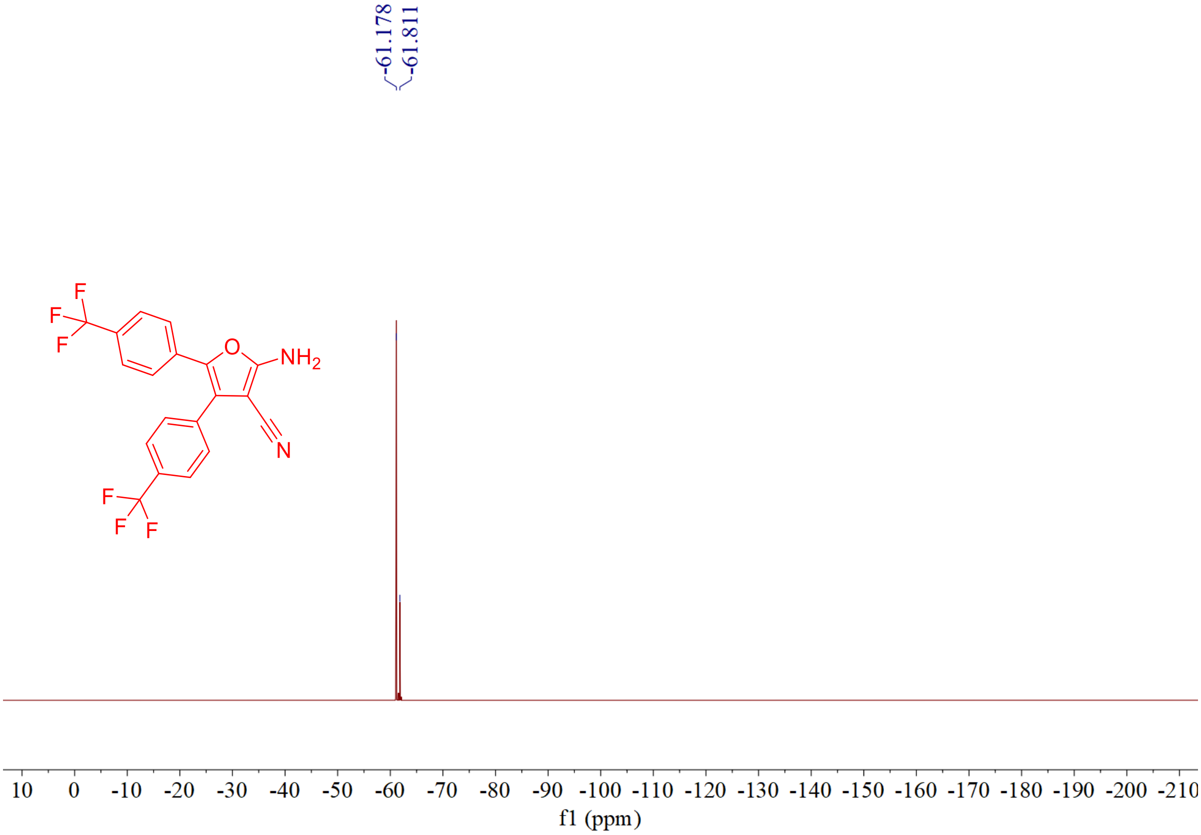

^{19}F NMR spectrum of compound **3i**

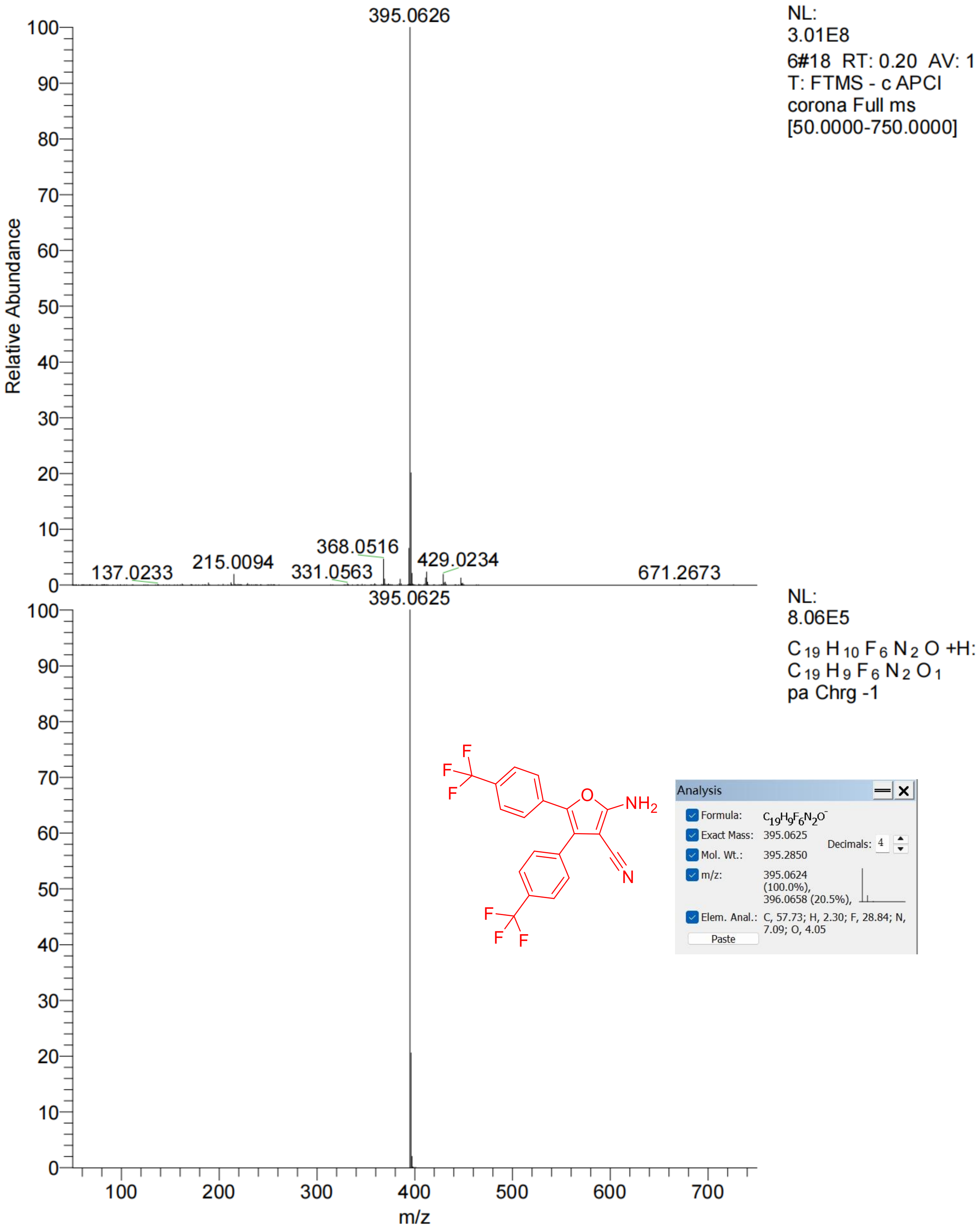

HR-MS spectrum of compound **3i**

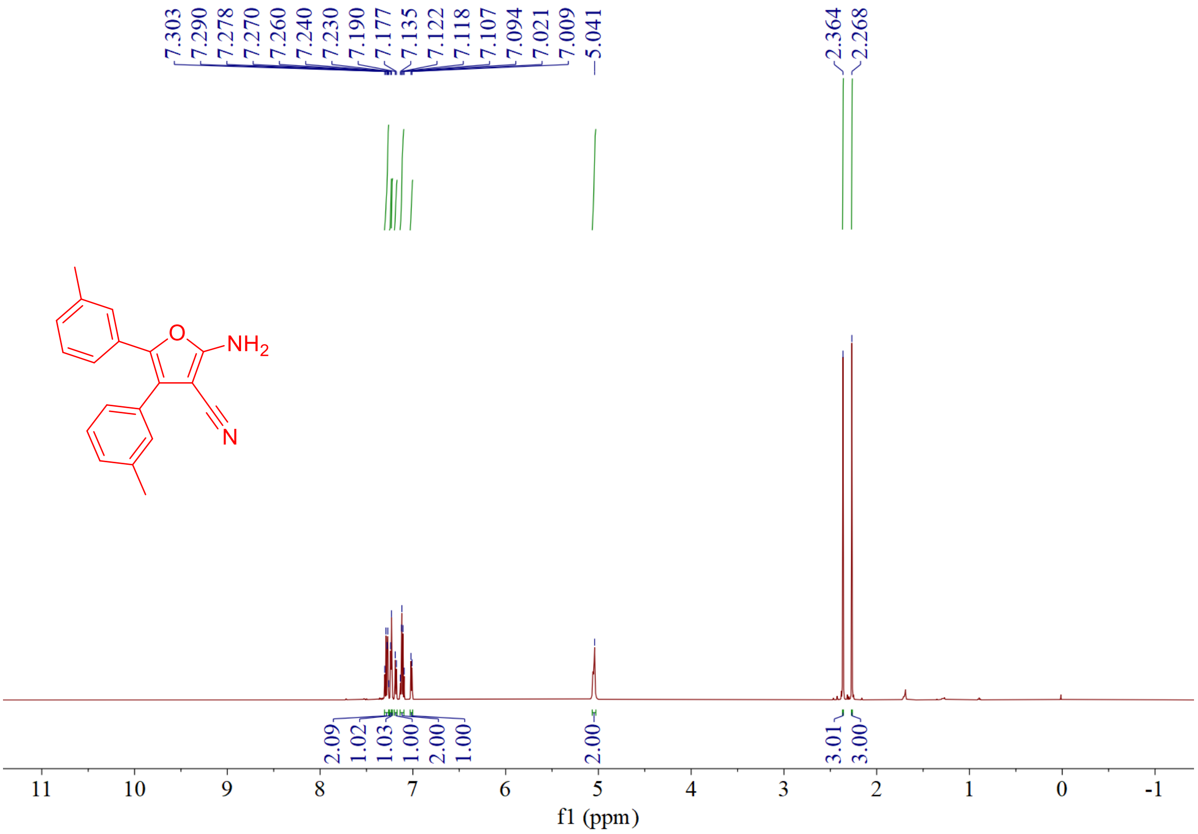

^{1}H NMR spectrum of compound **3j**

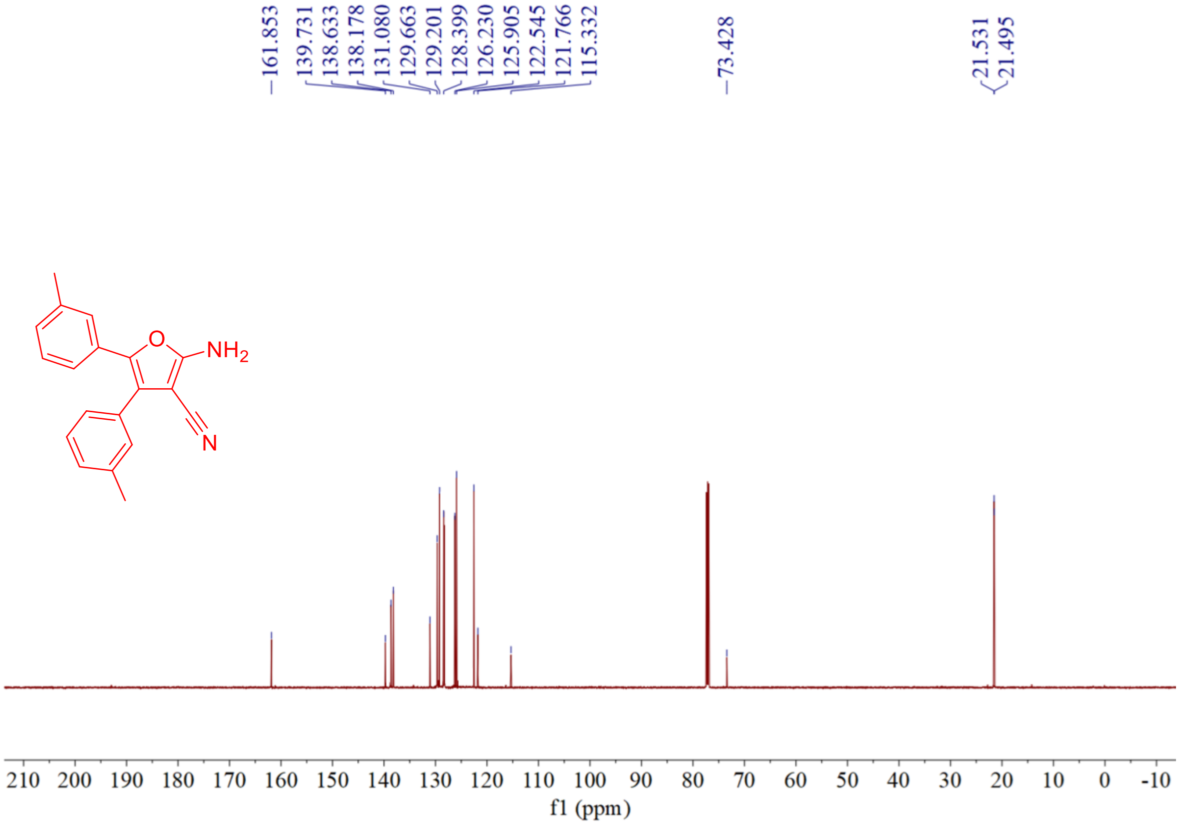

^{13}C NMR spectrum of compound **3j**

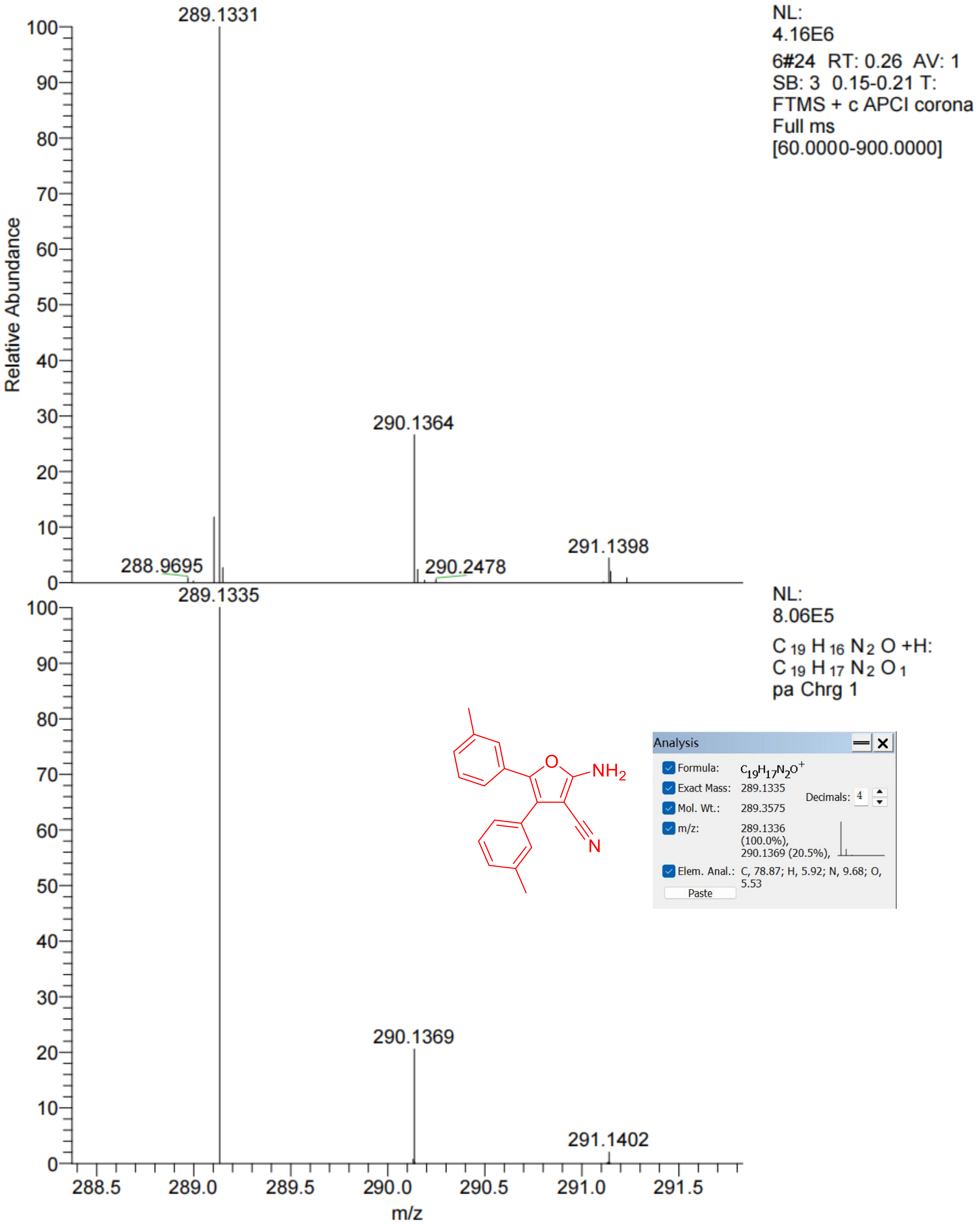

HR-MS spectrum of compound **3j**

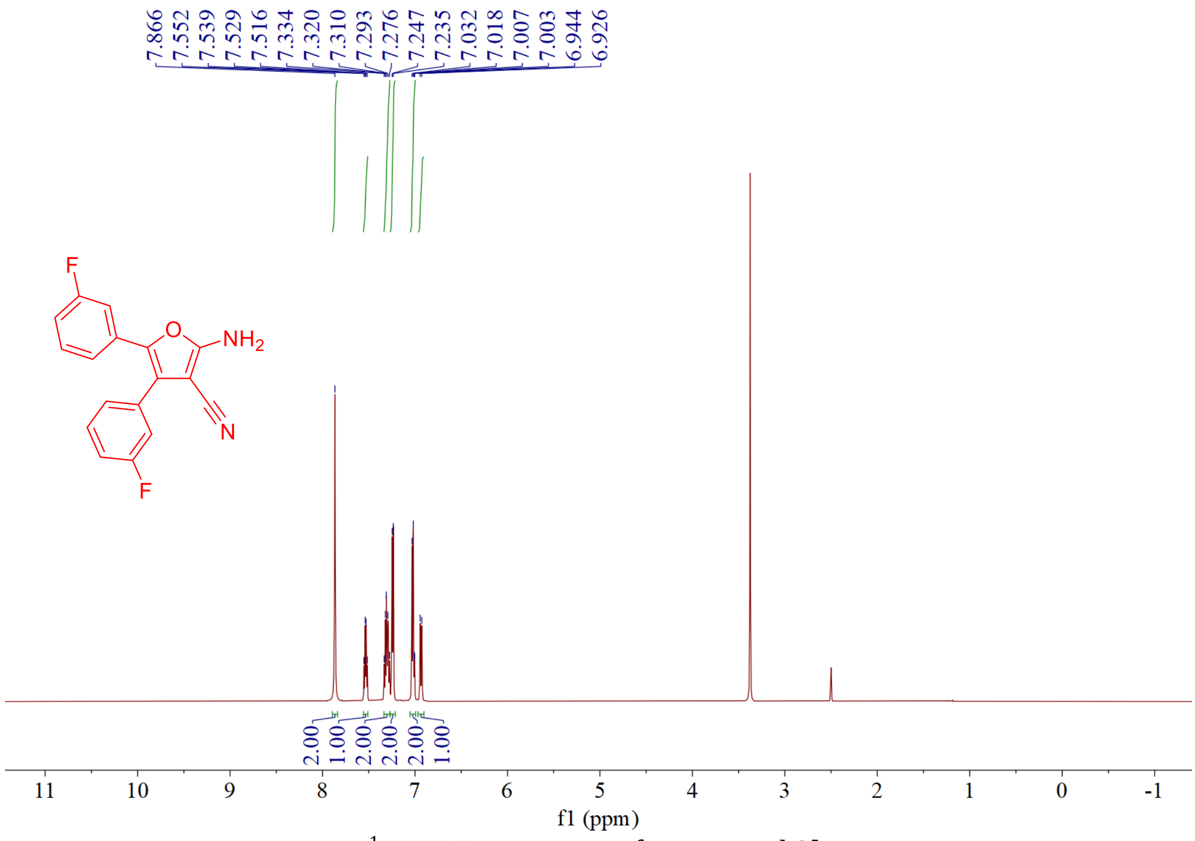

^{1}H NMR spectrum of compound **3k**

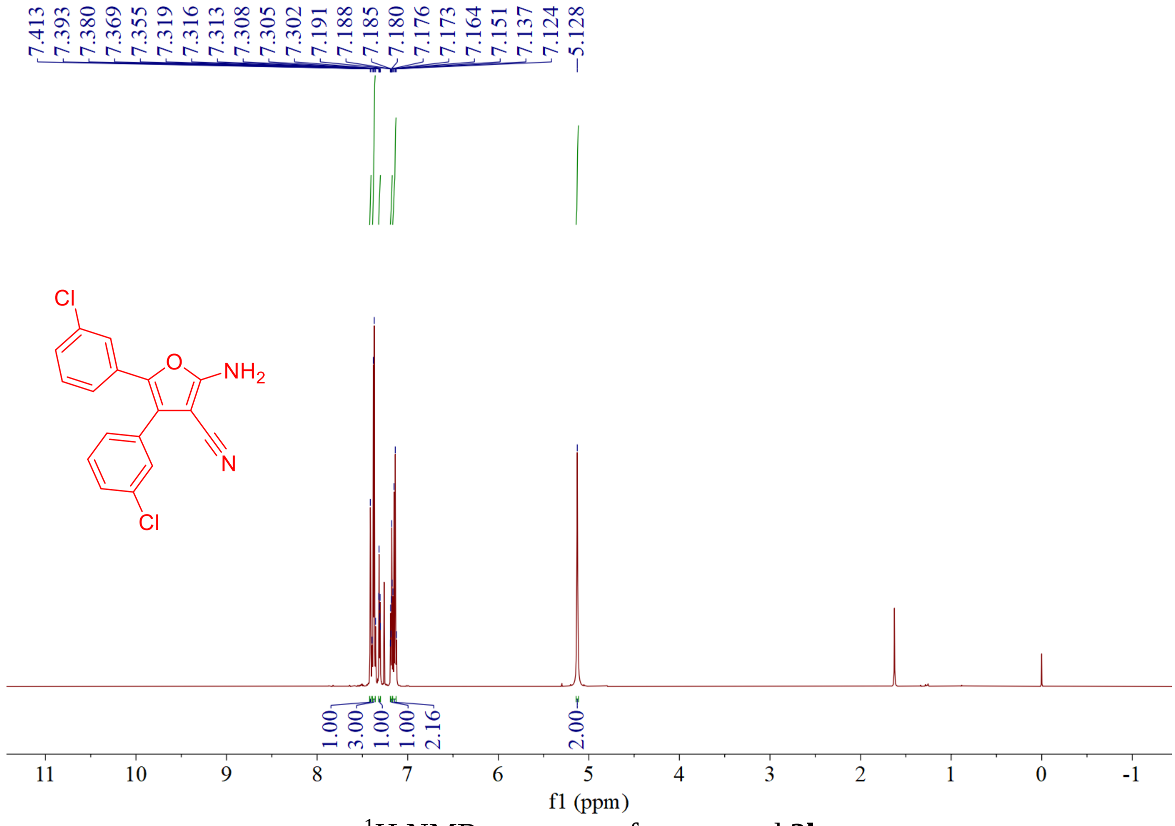

^{1}H NMR spectrum of compound **3l**

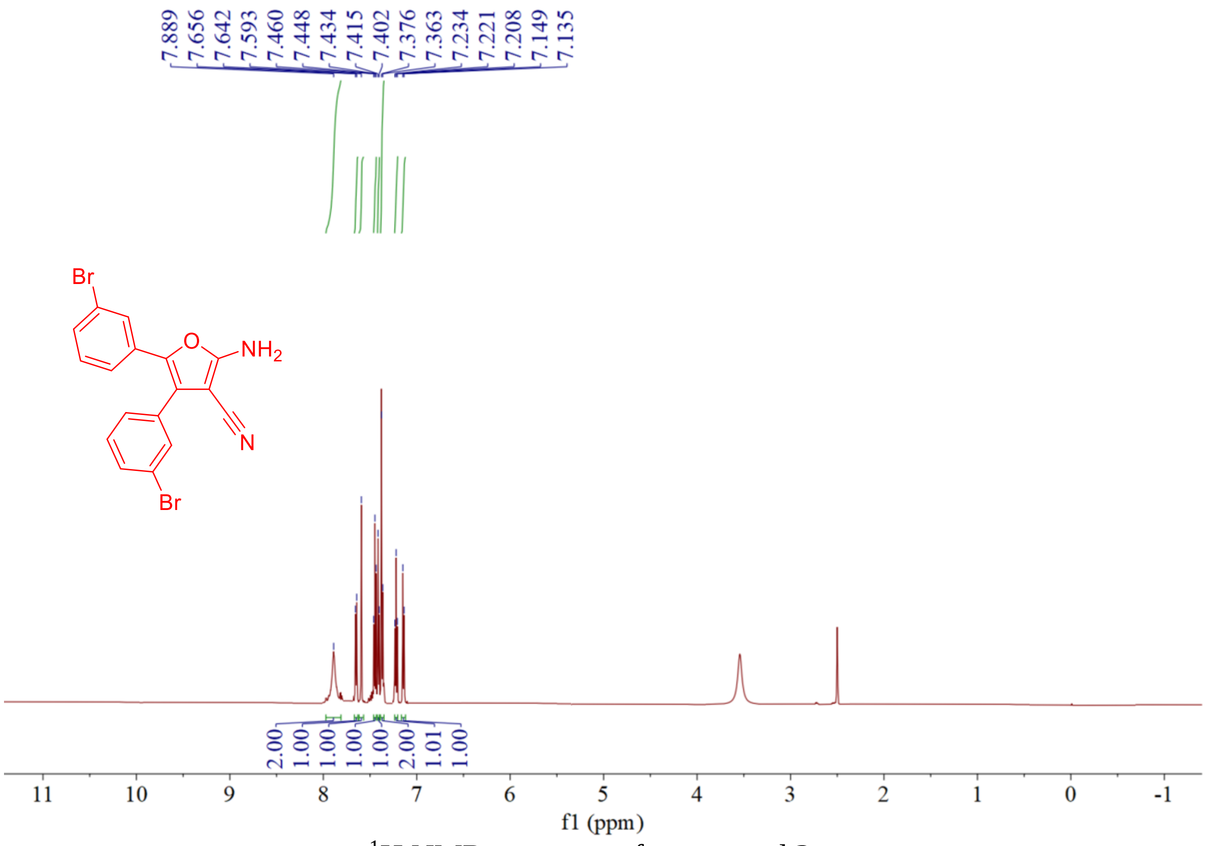

^{1}H NMR spectrum of compound **3m**

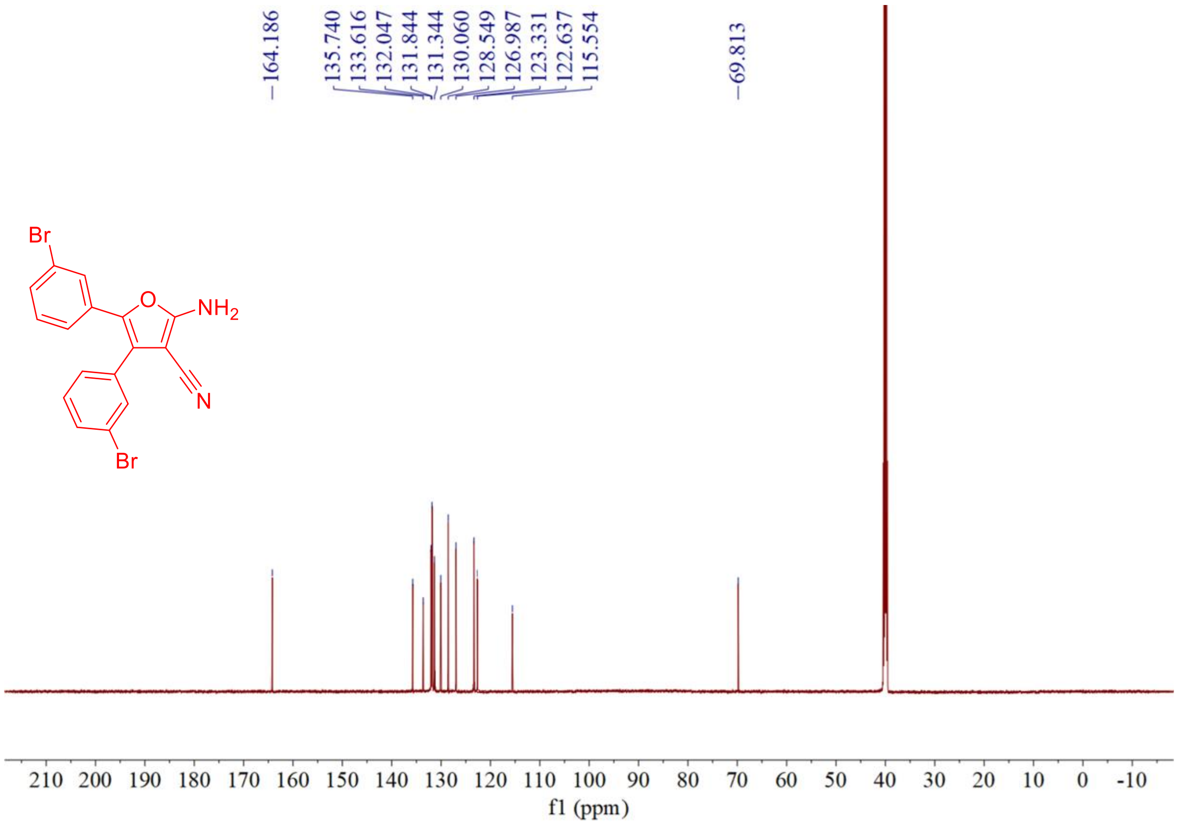

^{13}C NMR spectrum of compound **3m**

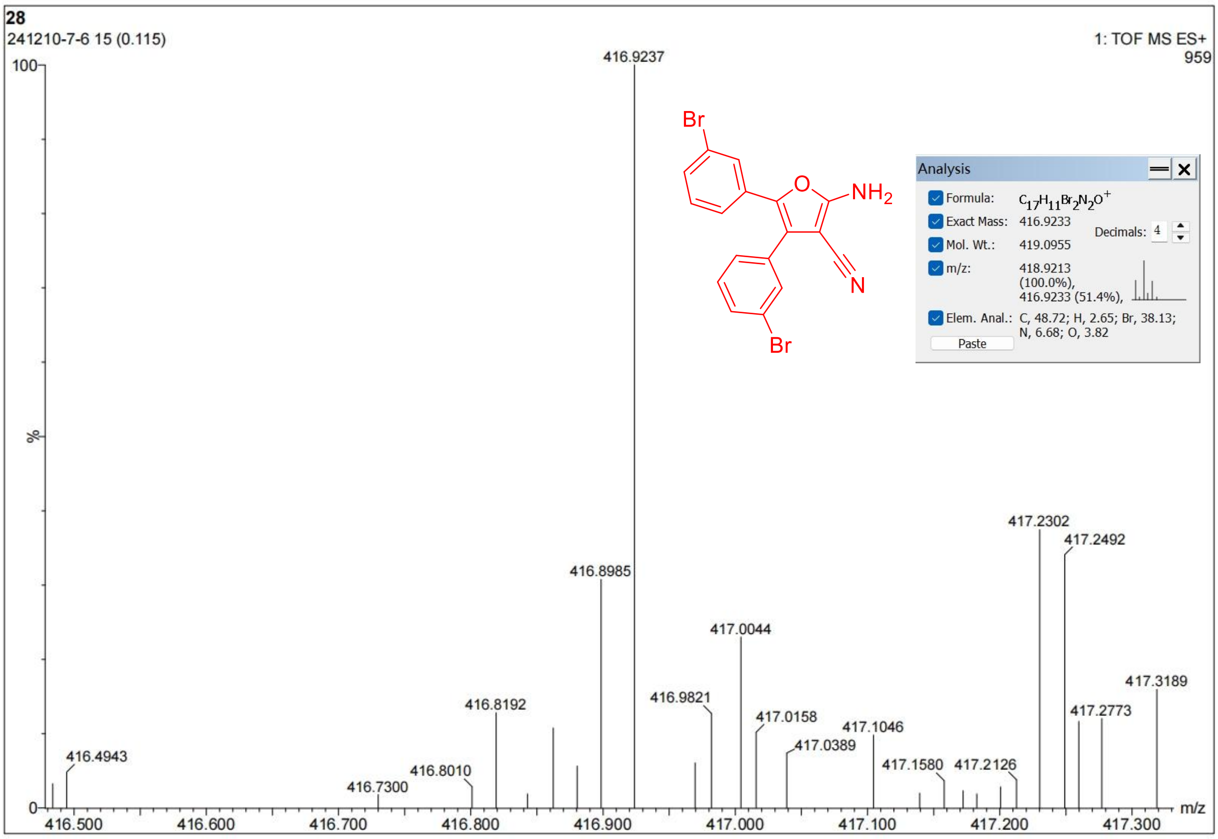

HR-MS spectrum of compound **3m**

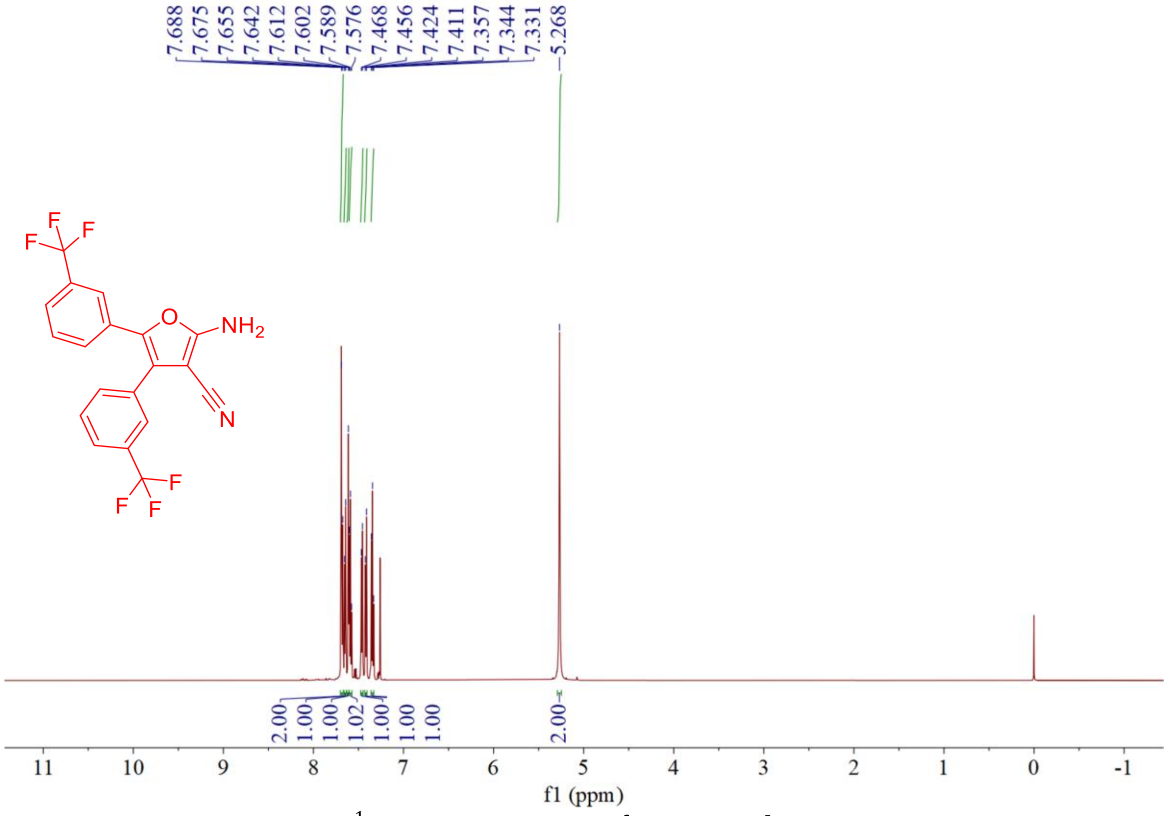

^{1}H NMR spectrum of compound **3n**

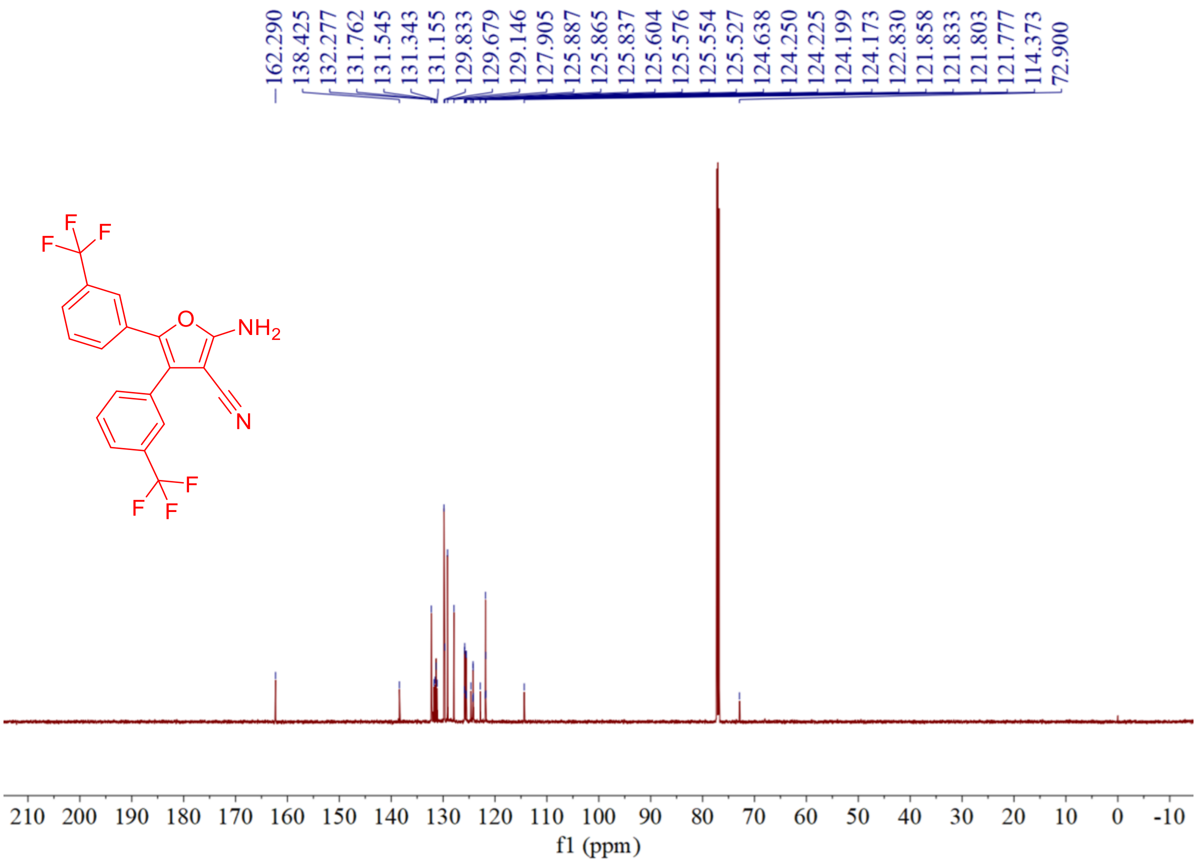

^{13}C NMR spectrum of compound **3n**

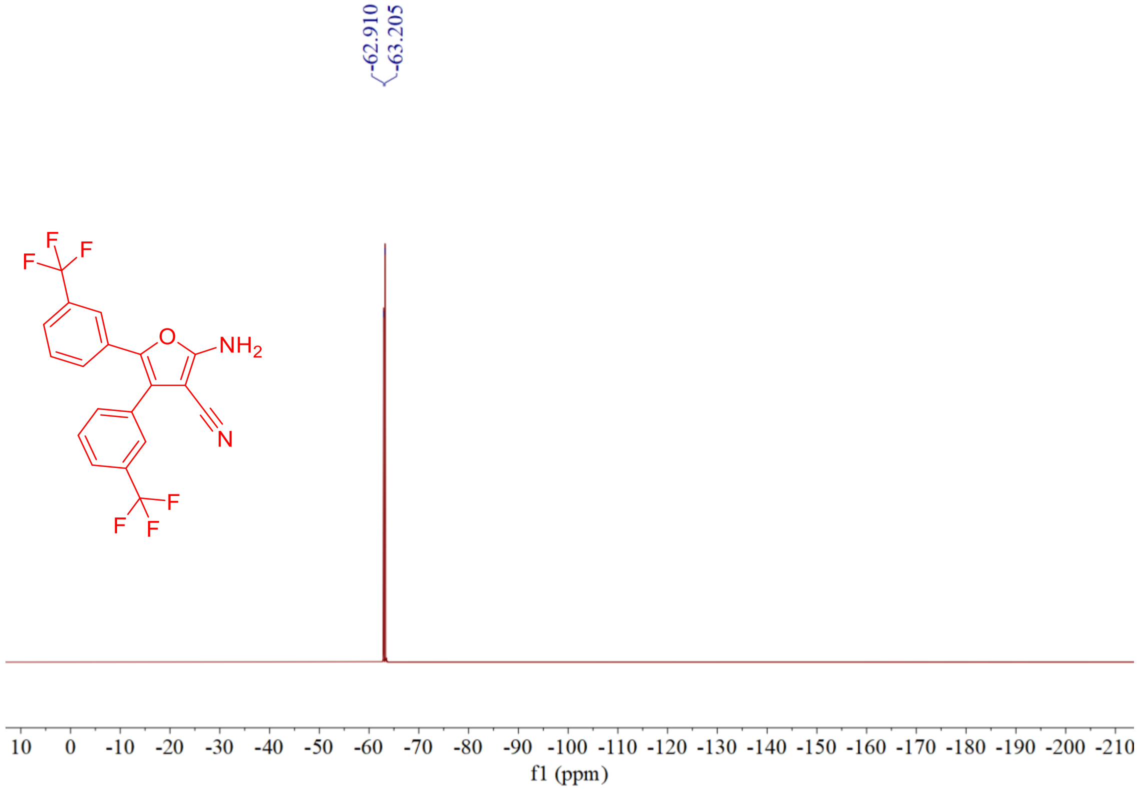

^{19}F NMR spectrum of compound **3n**

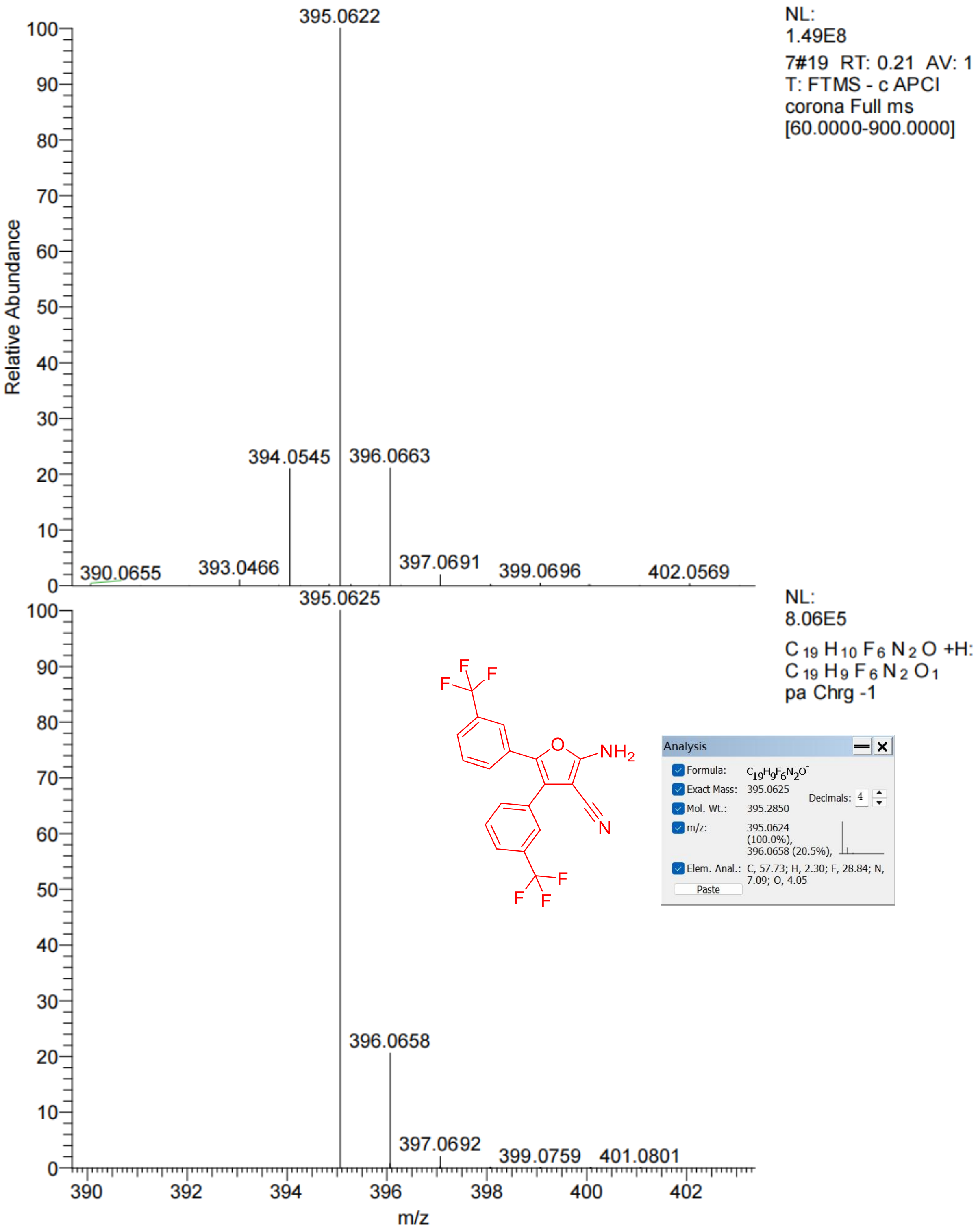

HR-MS spectrum of compound **3n**

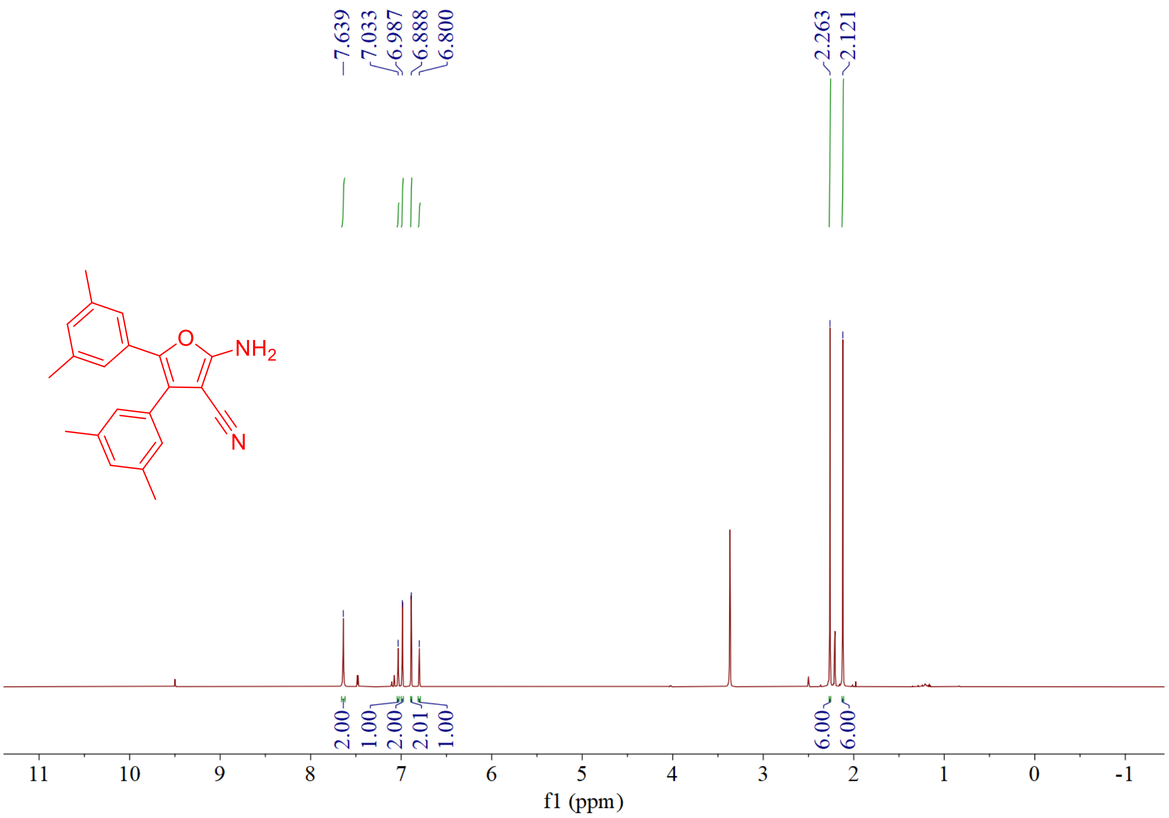

^{1}H NMR spectrum of compound **3o**

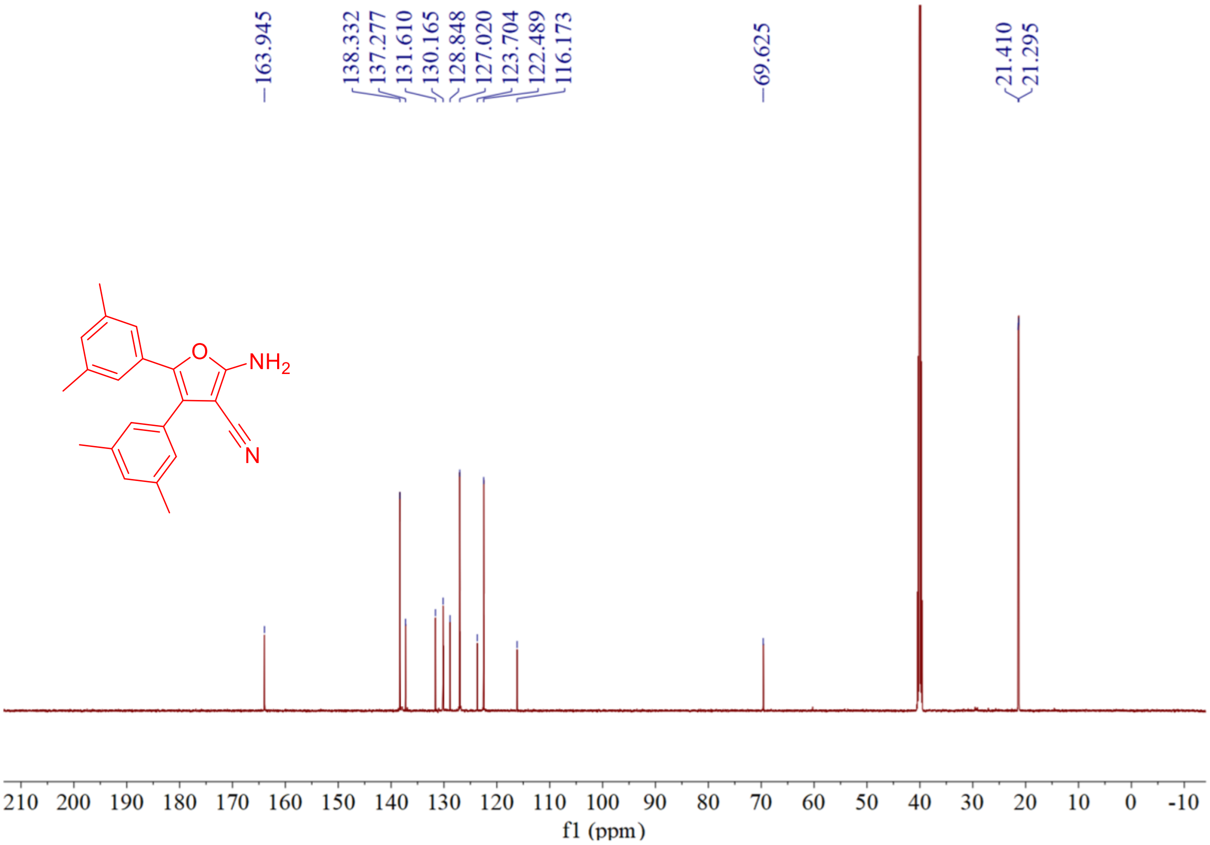

^{13}C NMR spectrum of compound **3o**

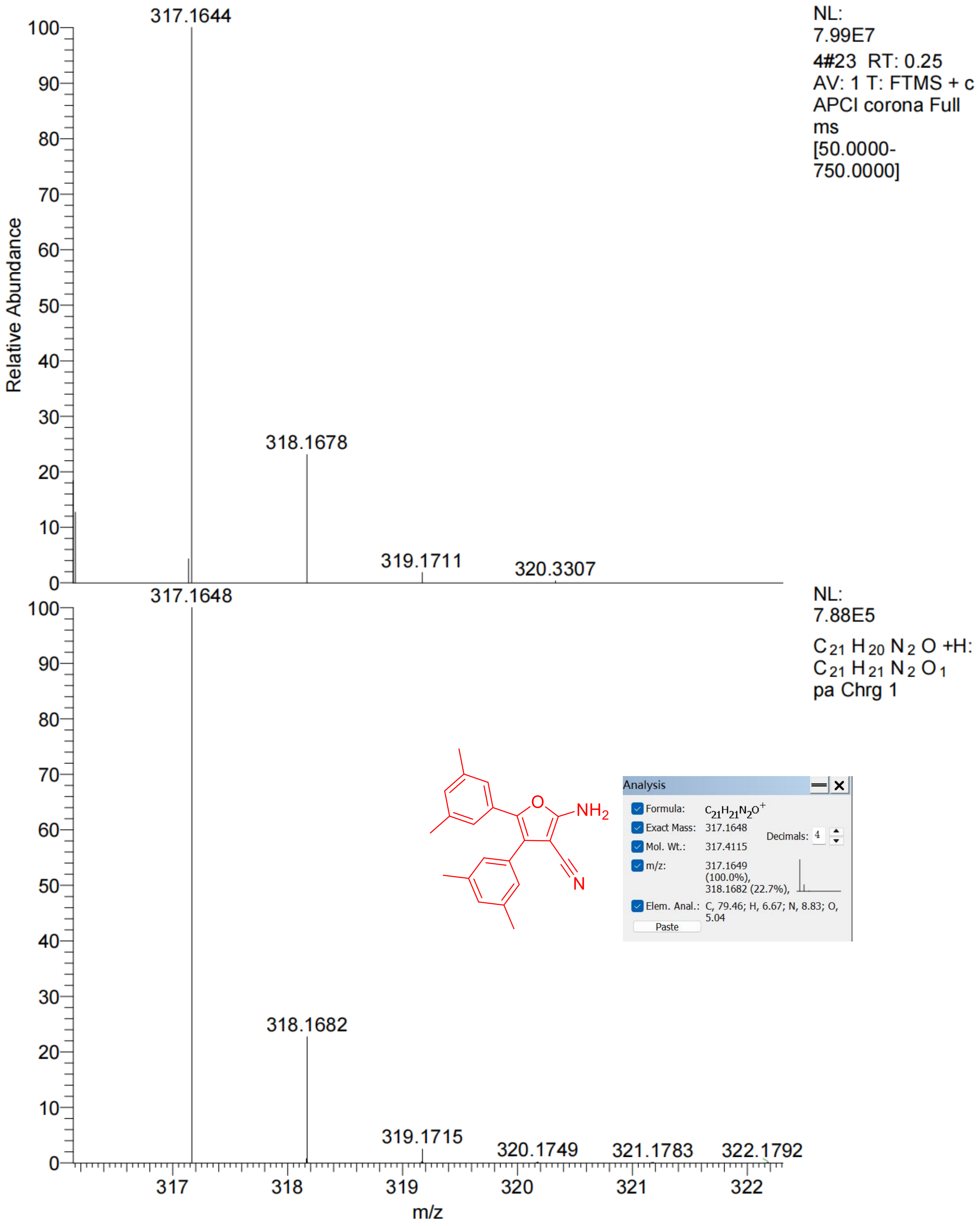

HR-MS spectrum of compound **3o**

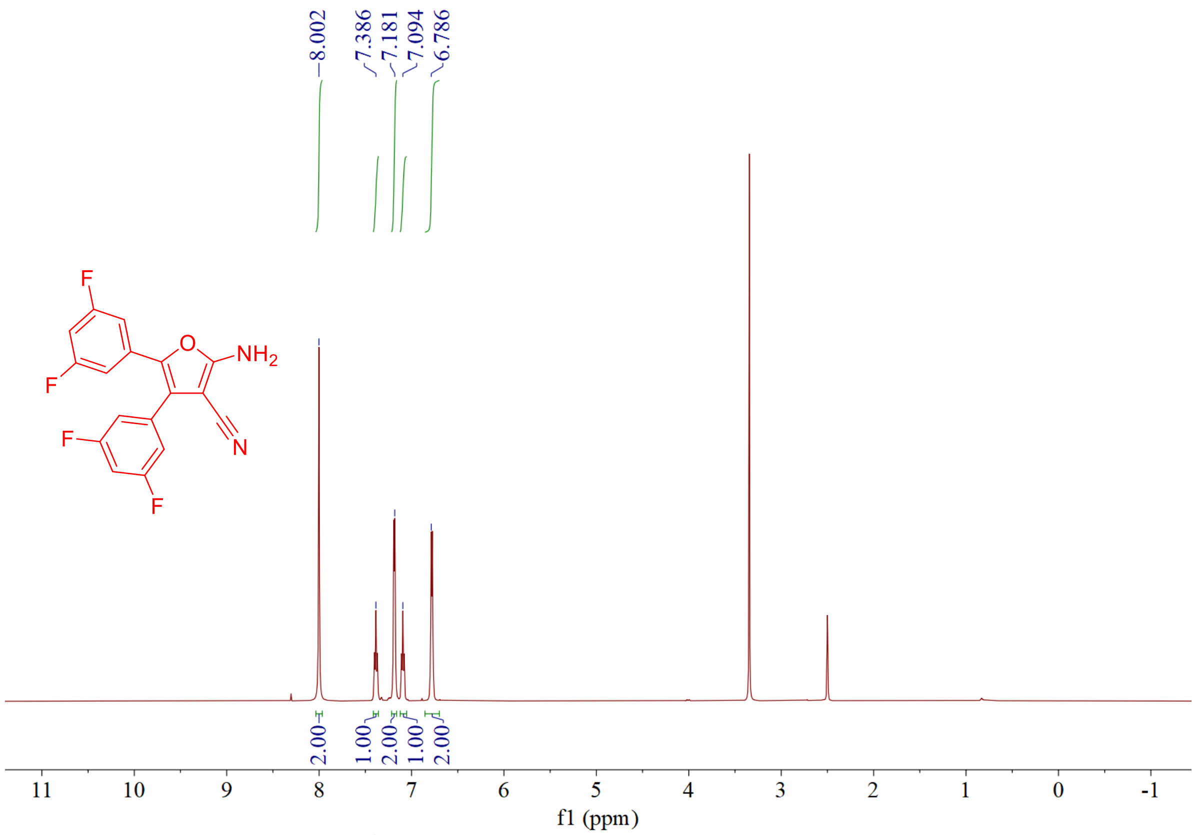

^{1}H NMR spectrum of compound **3p**

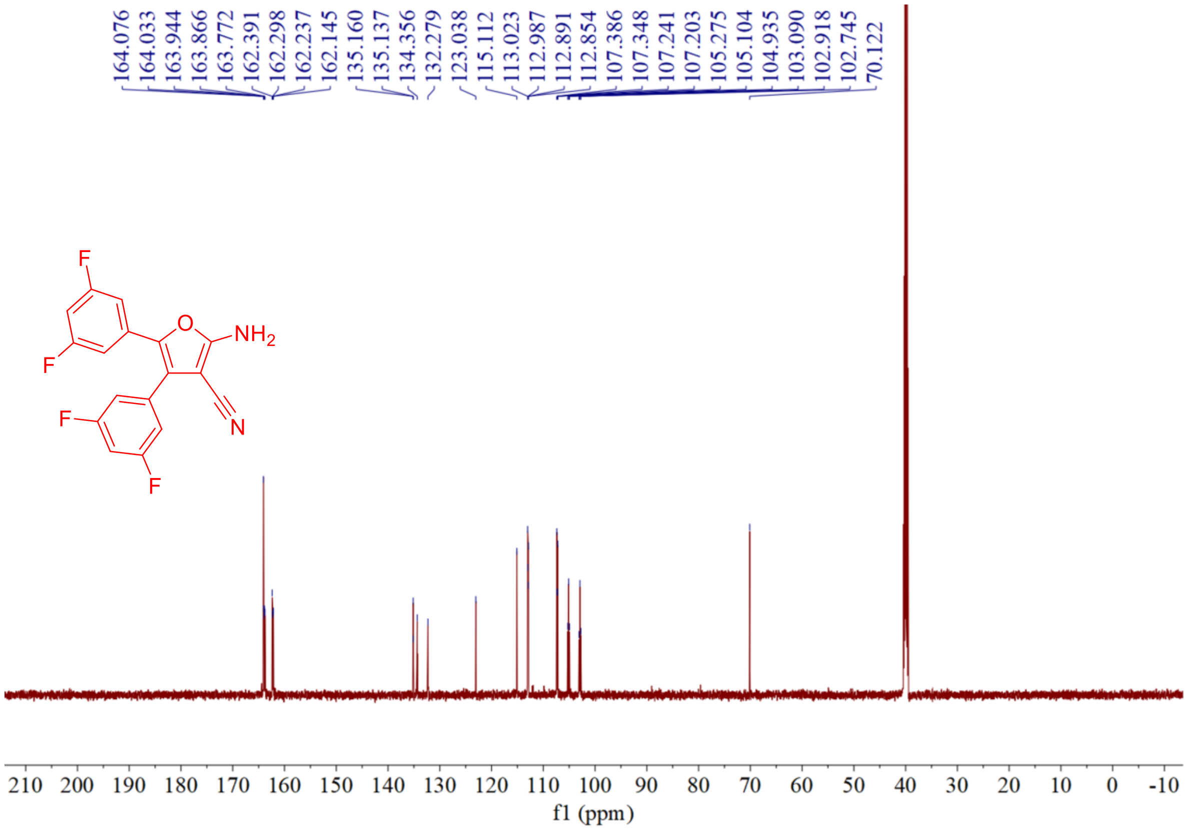

^{13}C NMR spectrum of compound **3p**

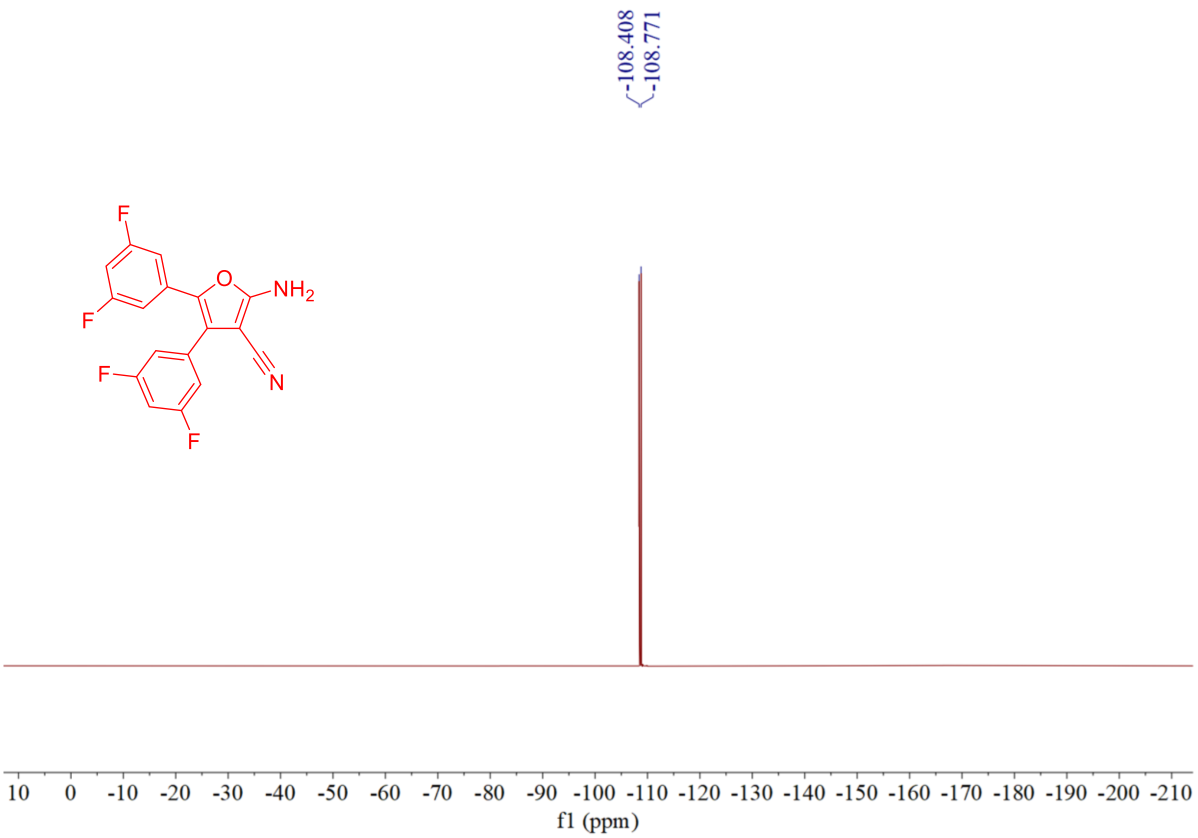

^{19}F NMR spectrum of compound **3p**

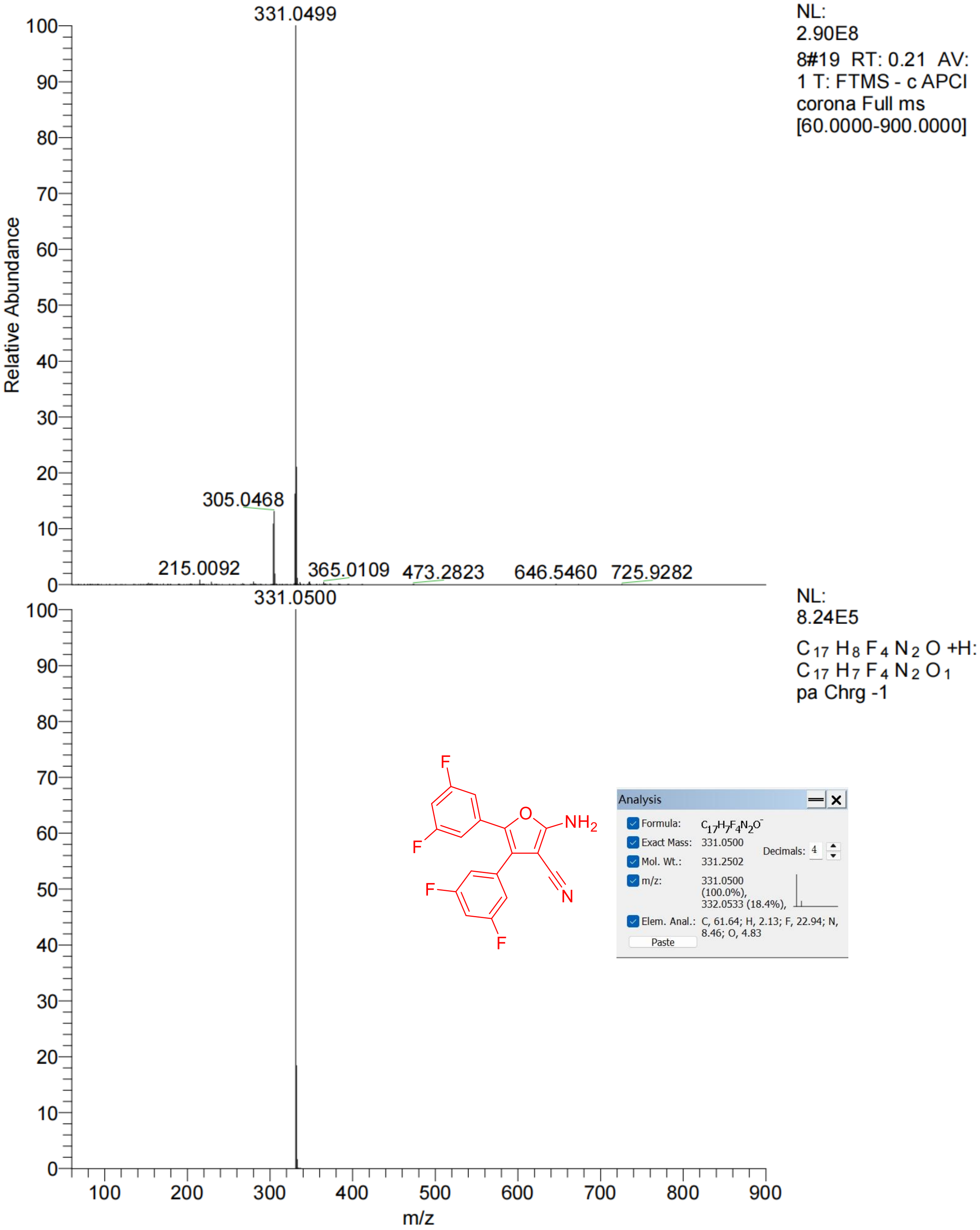

HR-MS spectrum of compound **3p**

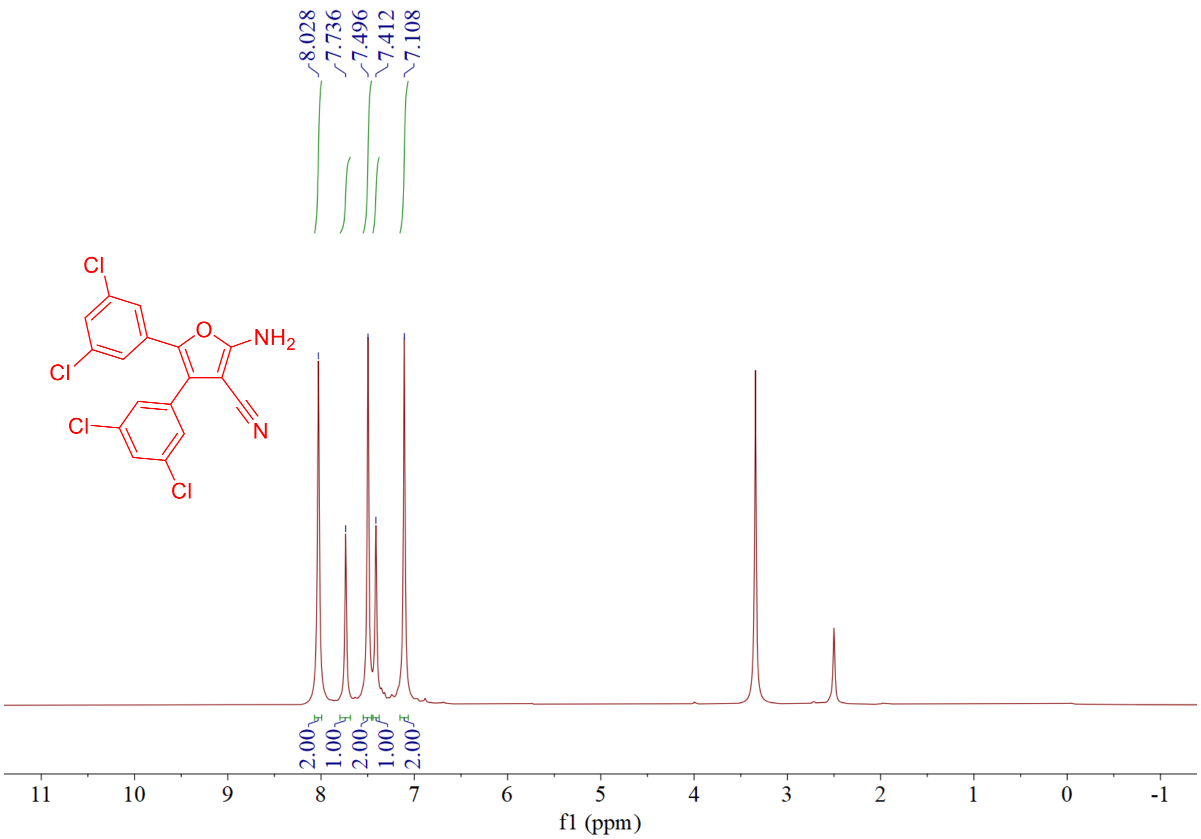

^{1}H NMR spectrum of compound **3q**

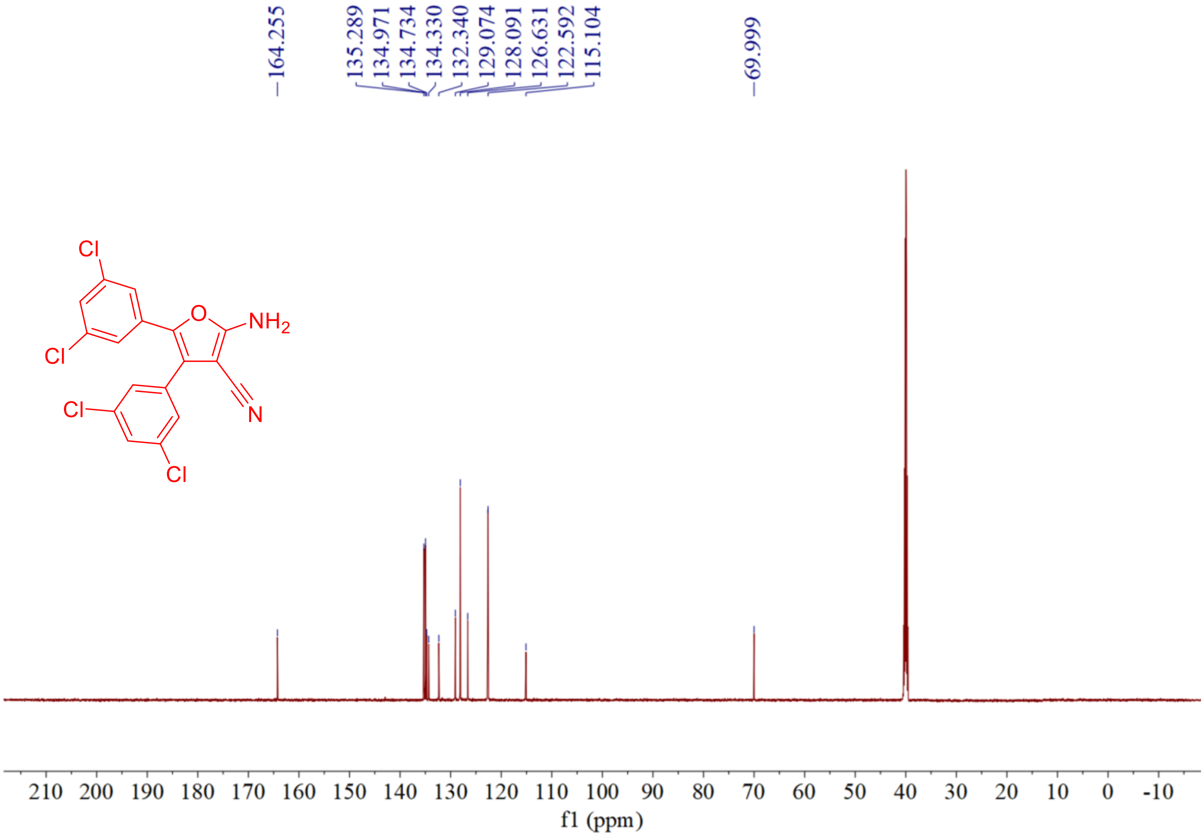

^{13}C NMR spectrum of compound **3q**

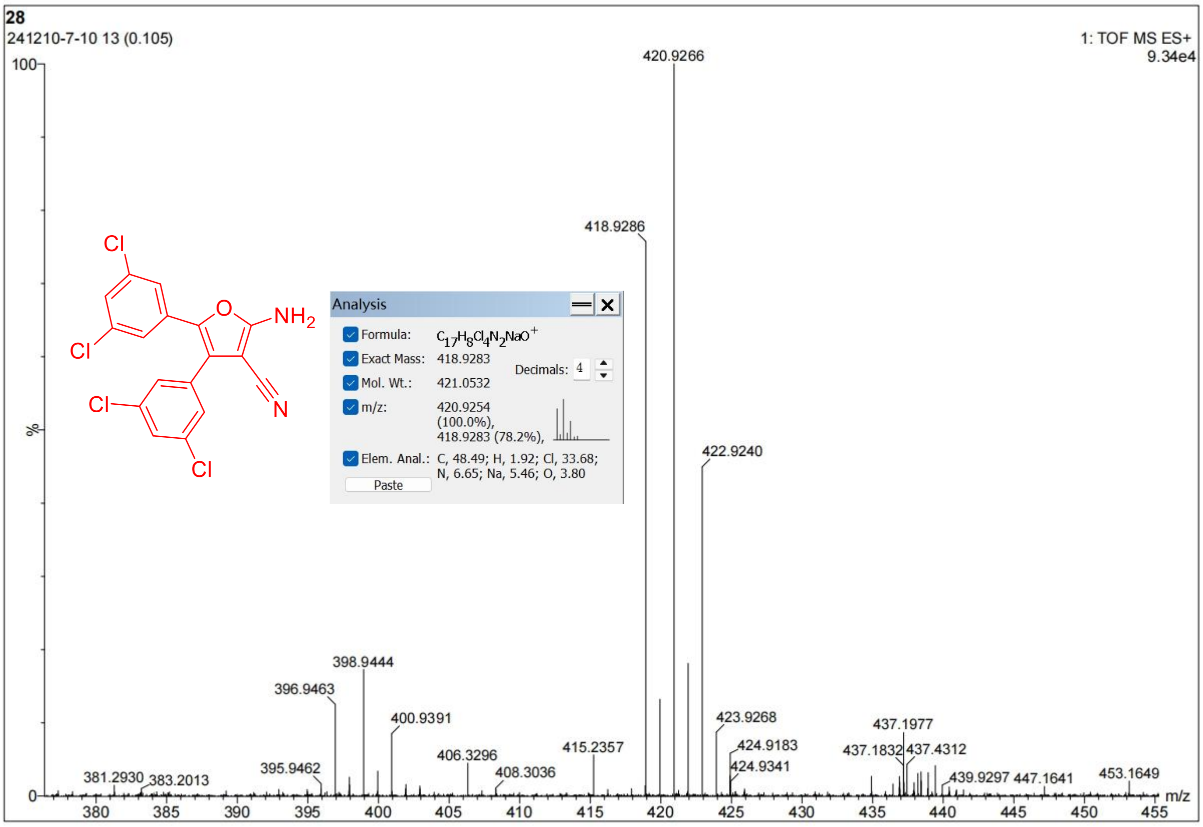

HR-MS spectrum of compound **3q**

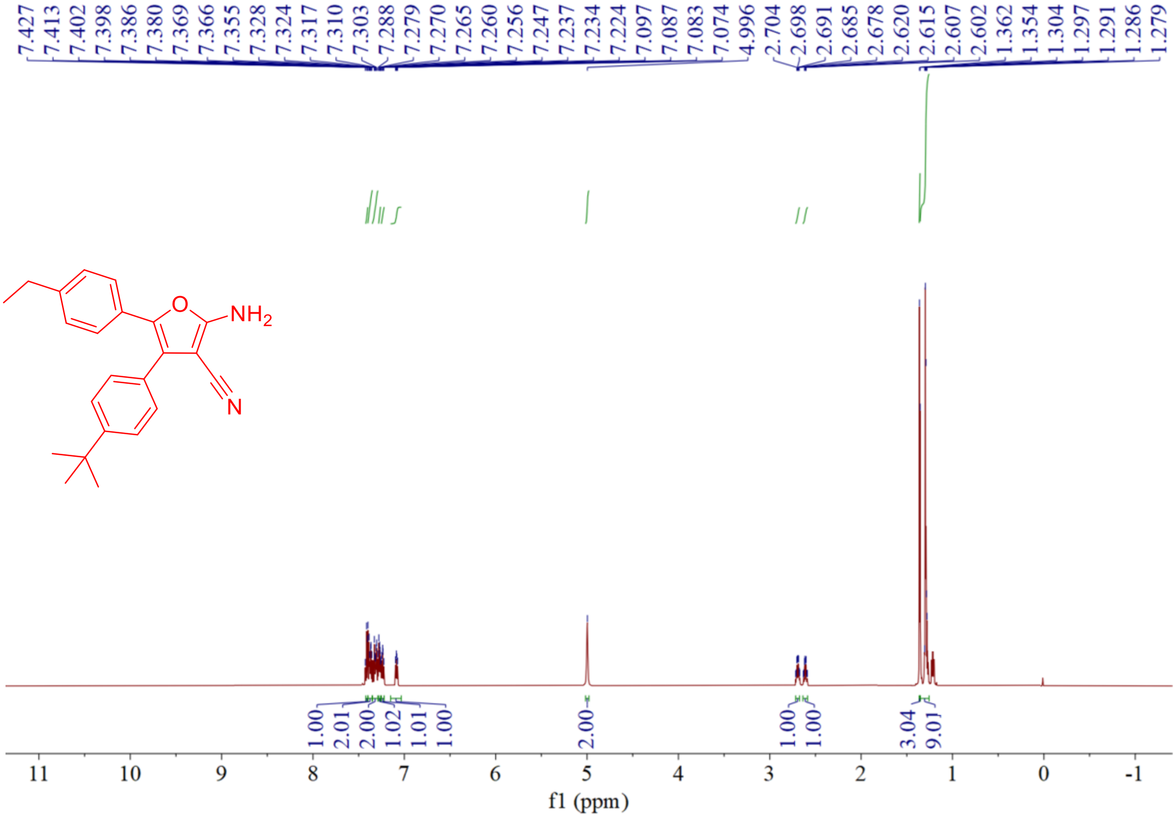

^{1}H NMR spectrum of compound **3r**

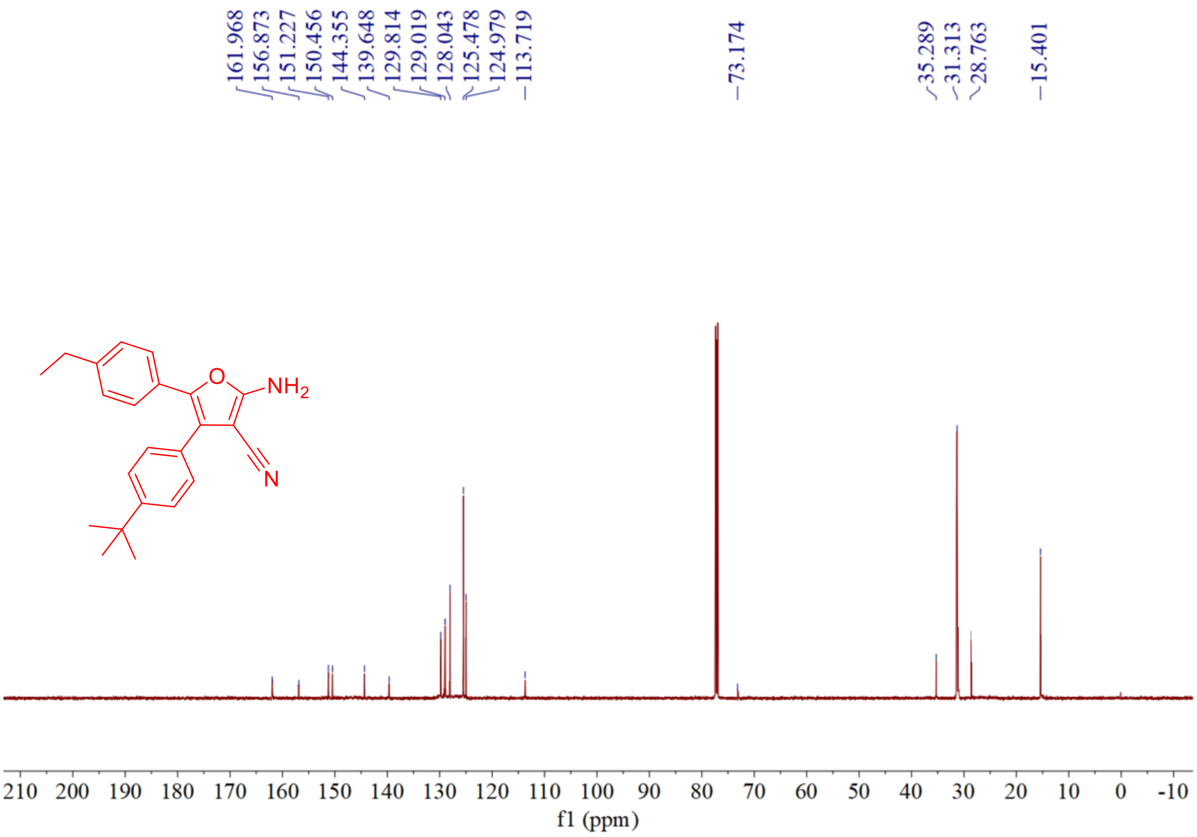

^{13}C NMR spectrum of compound **3r**

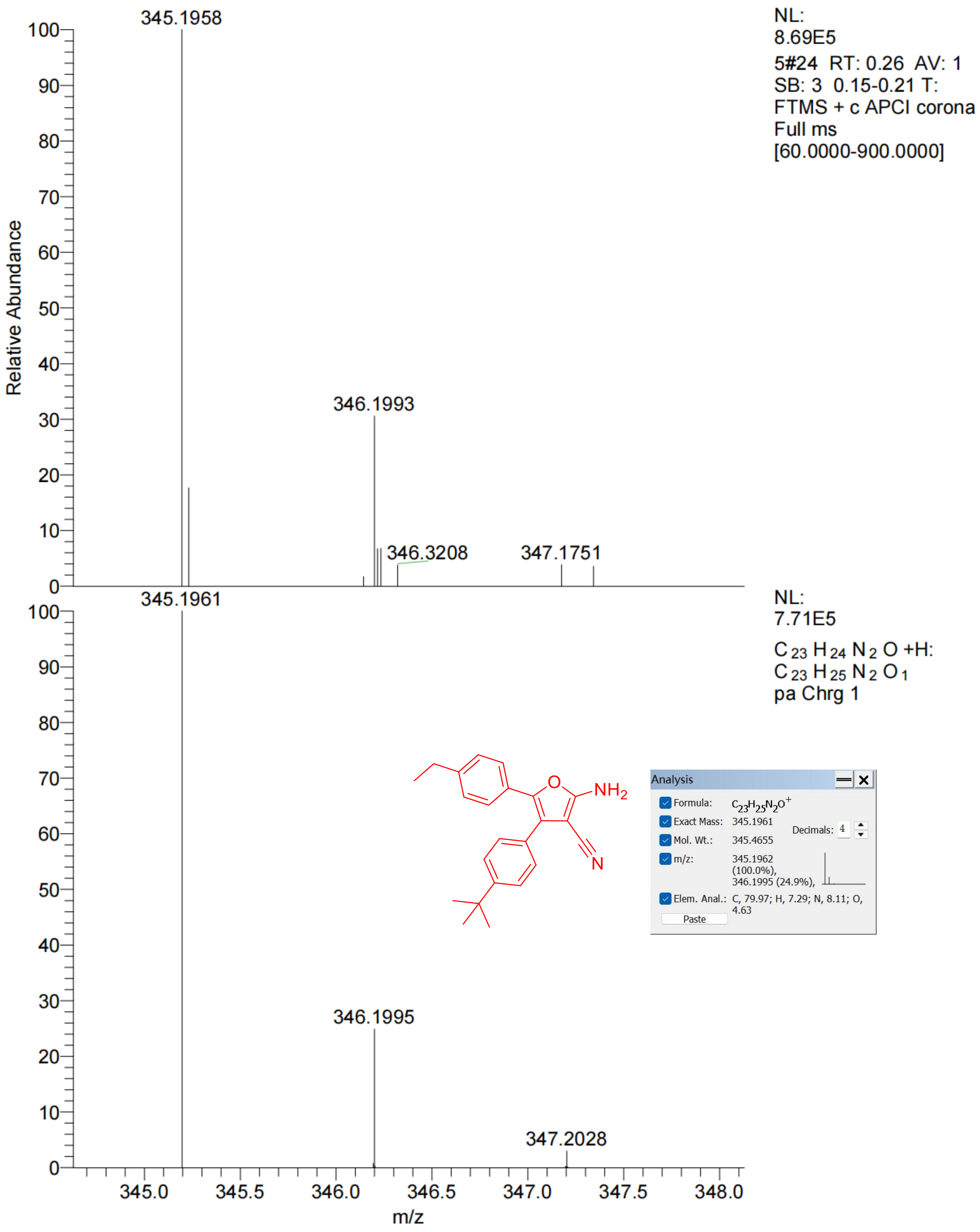

HR-MS spectrum of compound **3r**

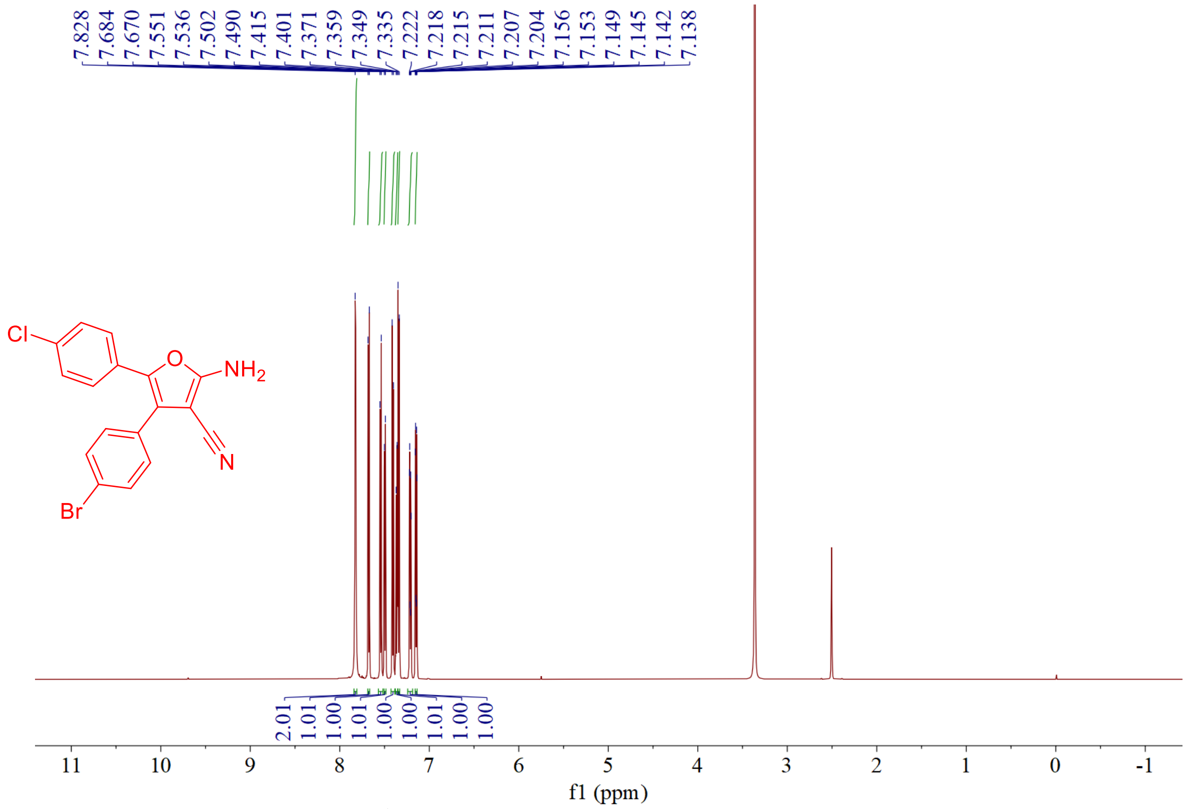

^{1}H NMR spectrum of compound **3s**

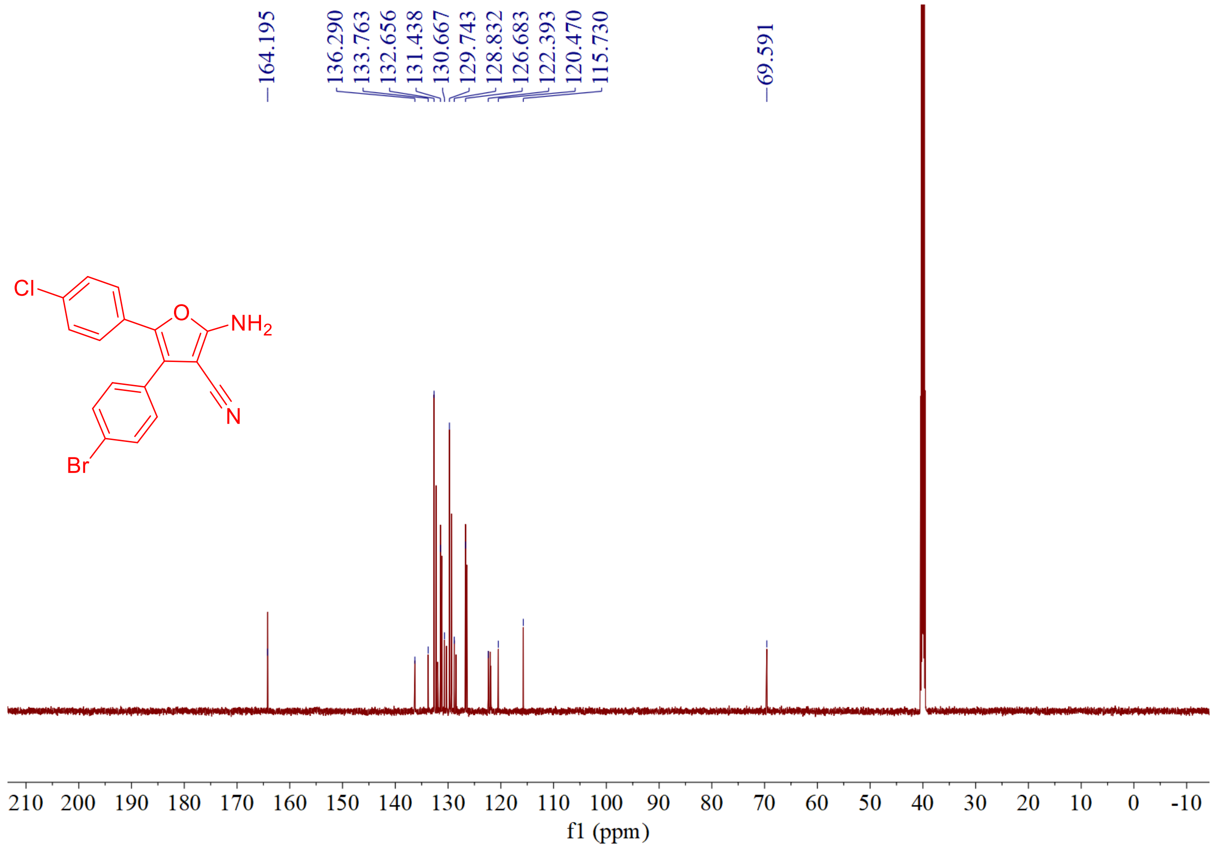

^{13}C NMR spectrum of compound **3s**

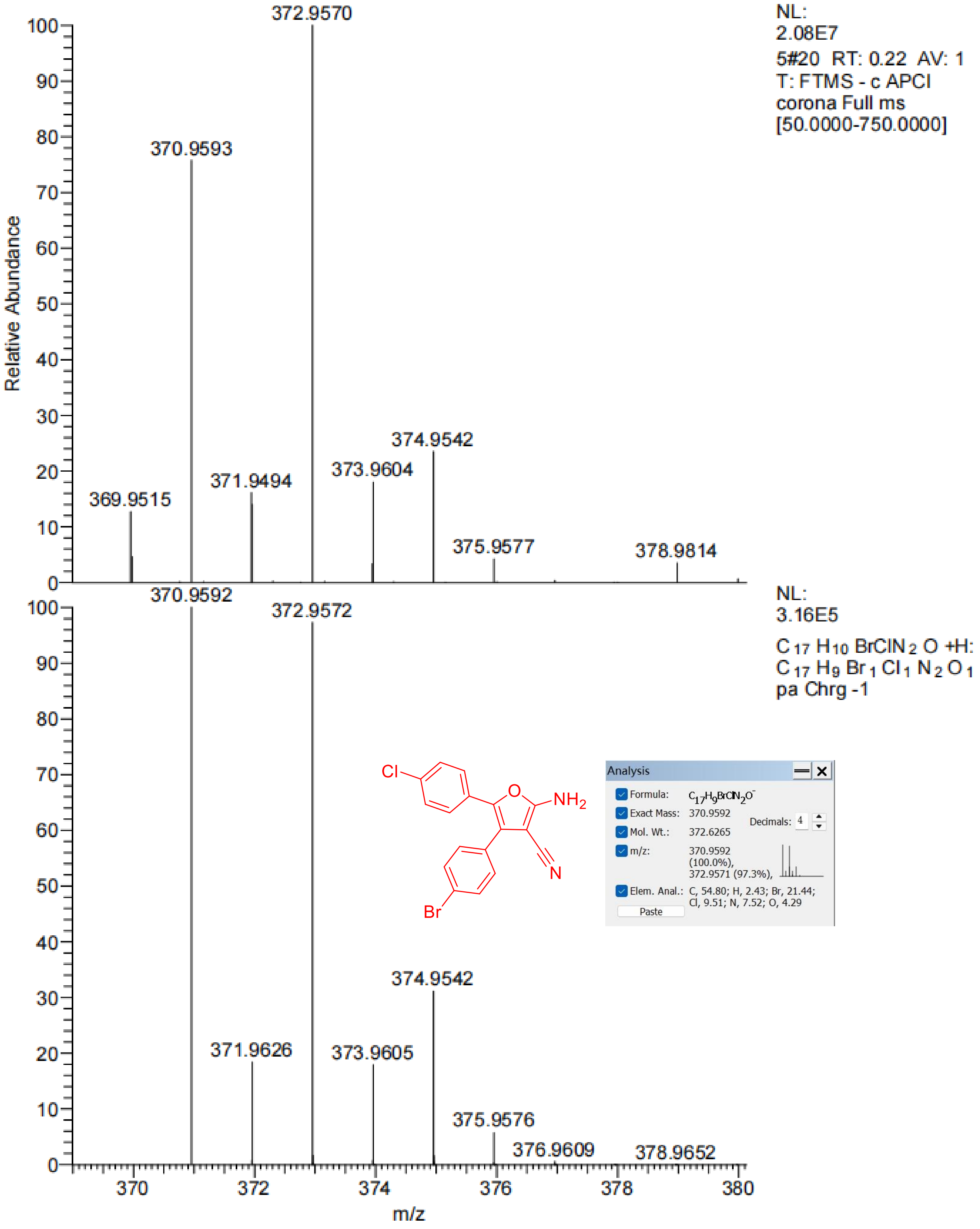

HR-MS spectrum of compound **3s**

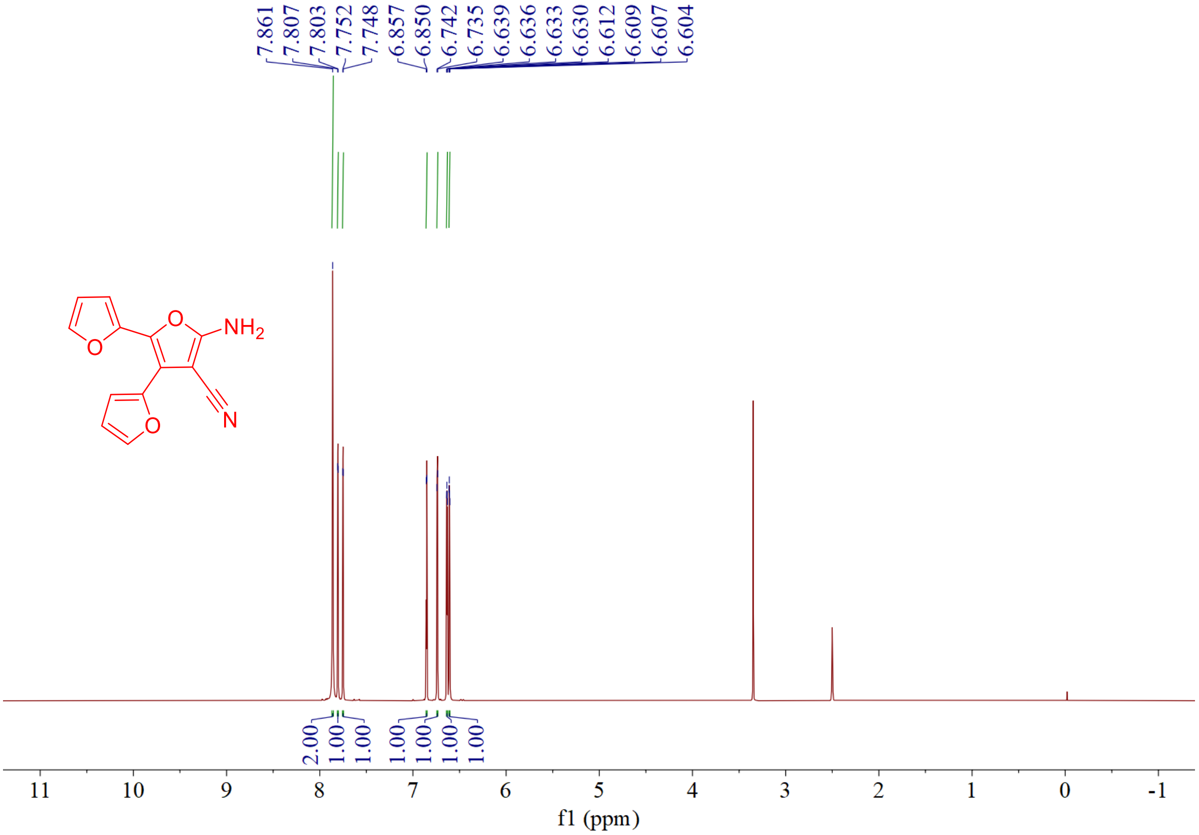

^{1}H NMR spectrum of compound **3t**

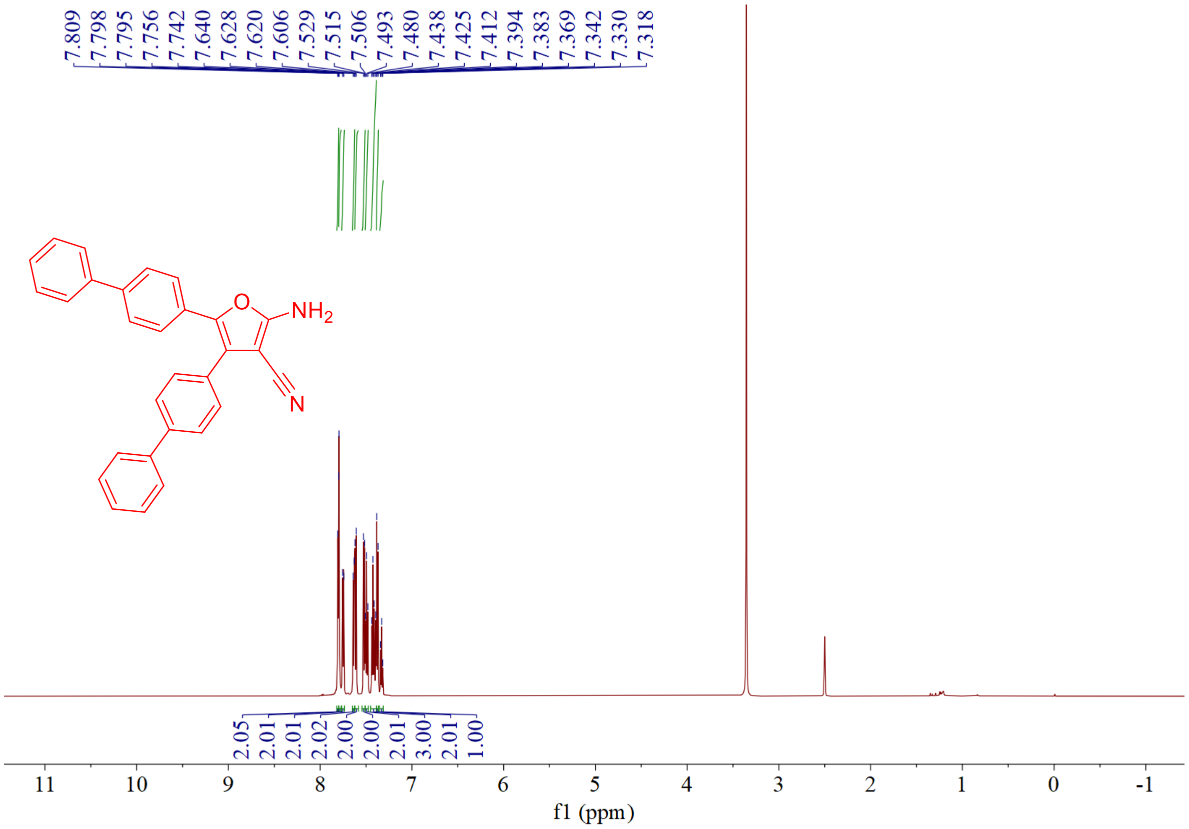

^{1}H NMR spectrum of compound **3u**

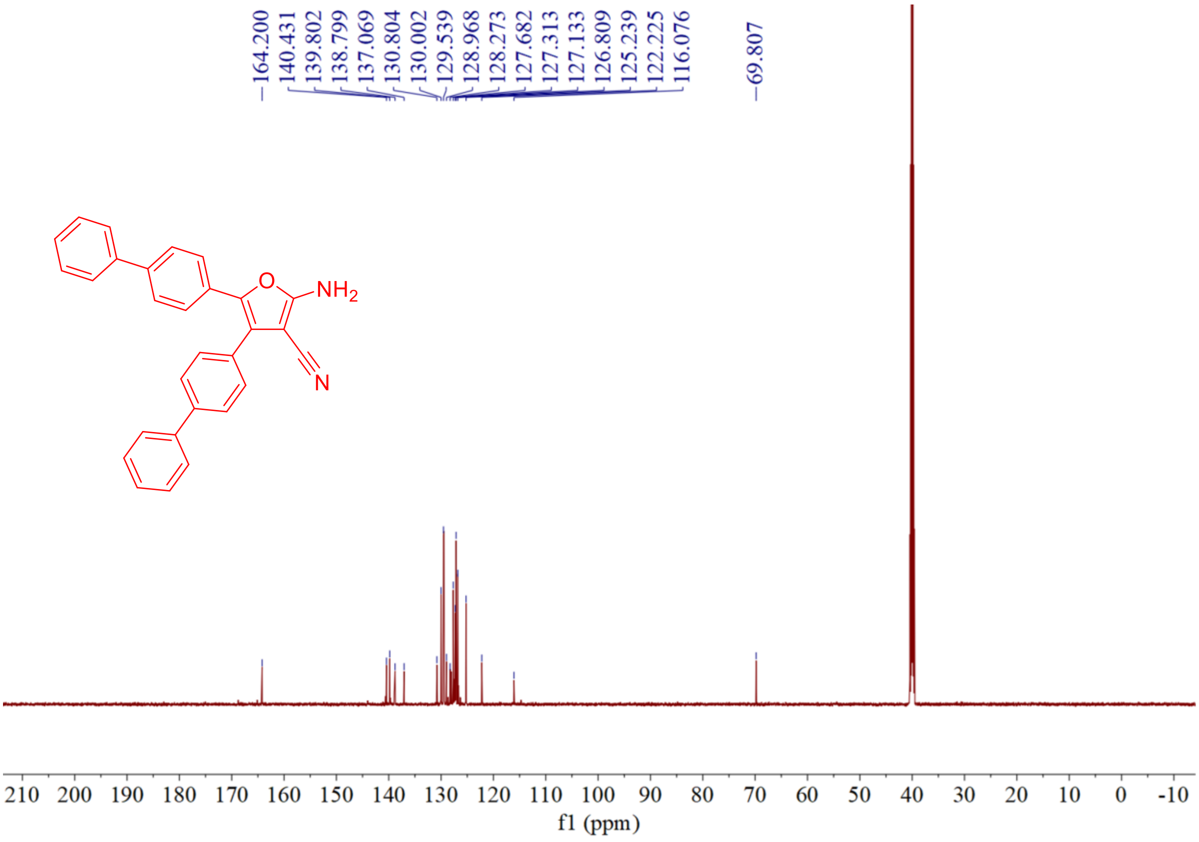

^{13}C NMR spectrum of compound **3u**

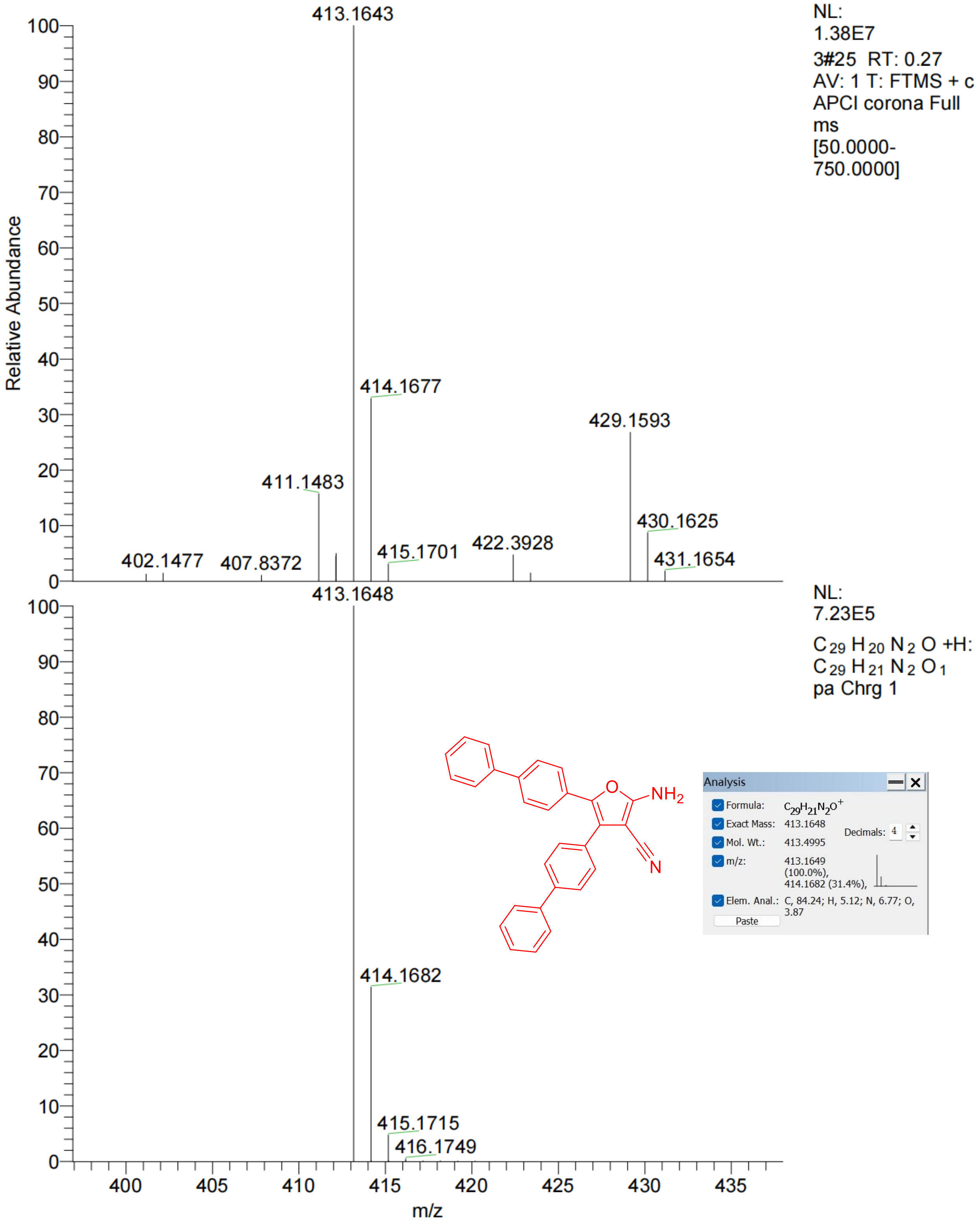

HR-MS spectrum of compound **3u**

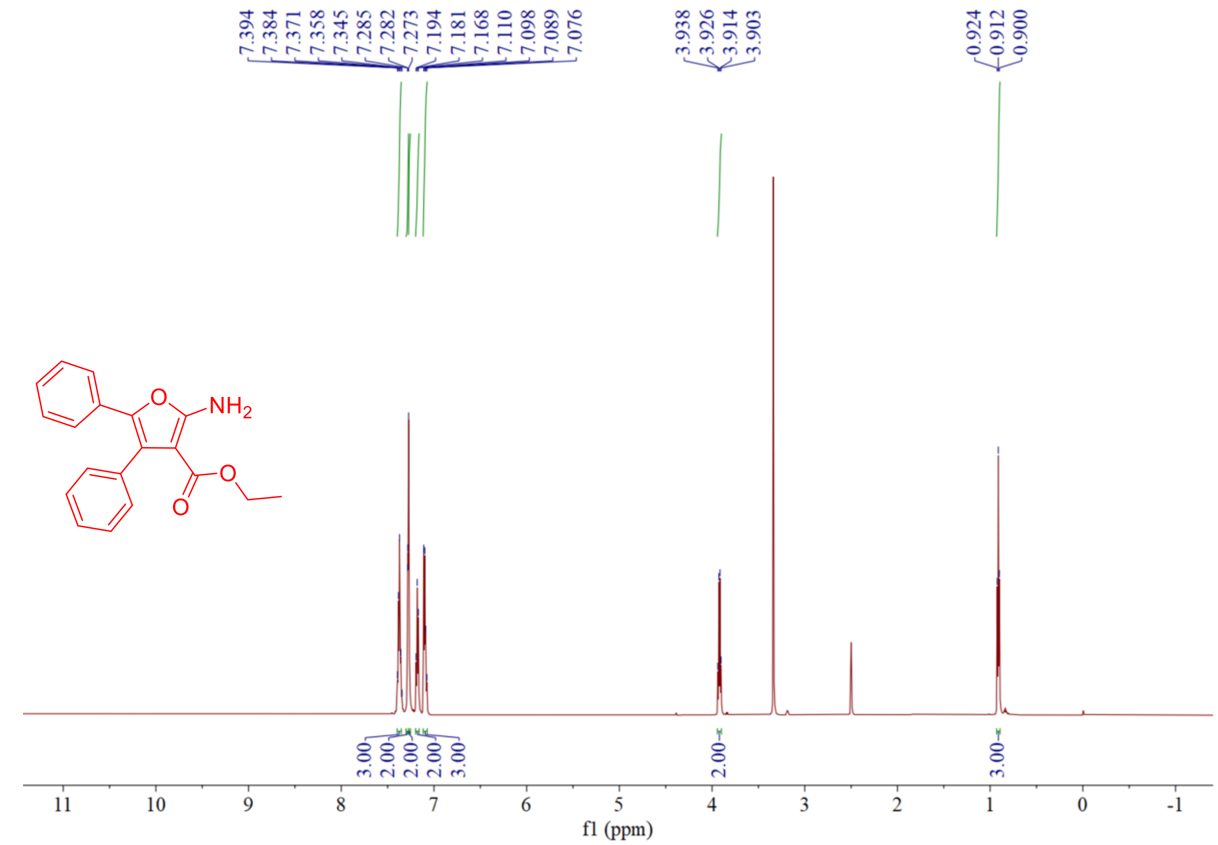

^{1}H NMR spectrum of compound **3v**

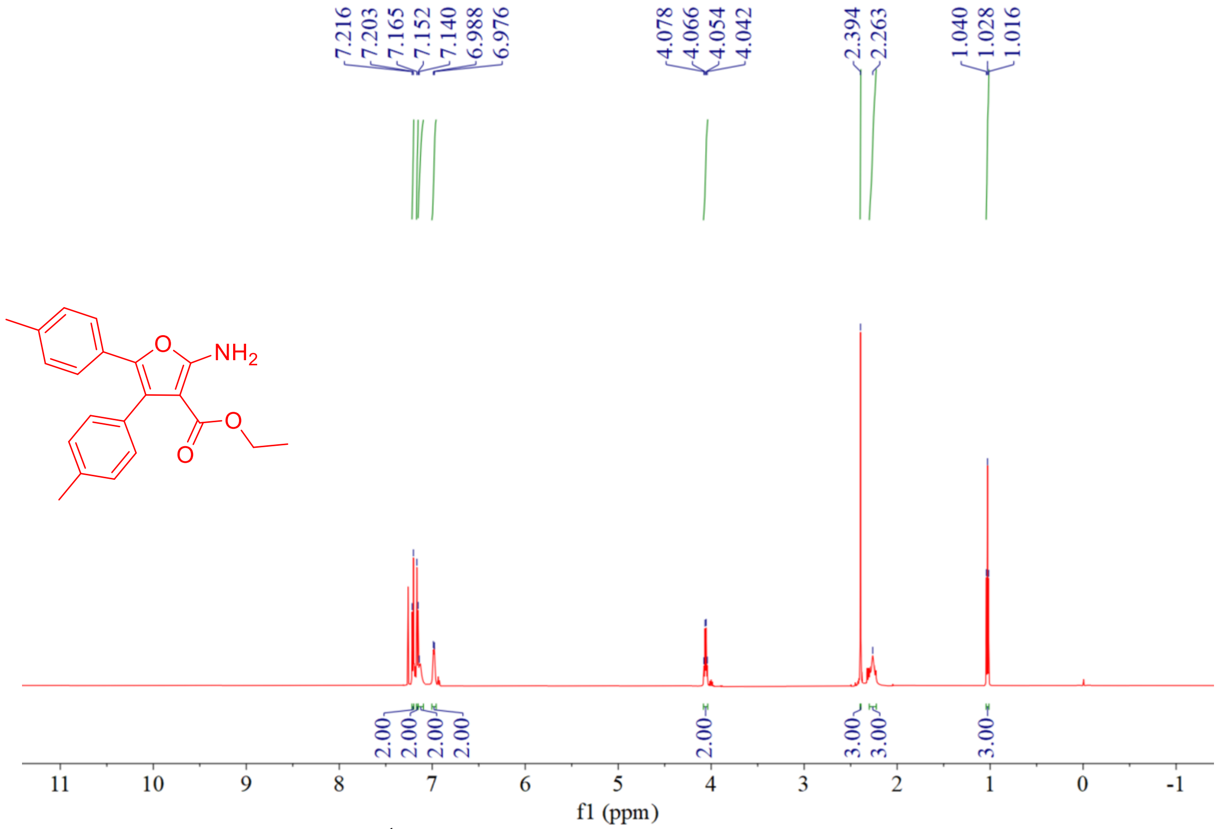

^{1}H NMR spectrum of compound **3w**

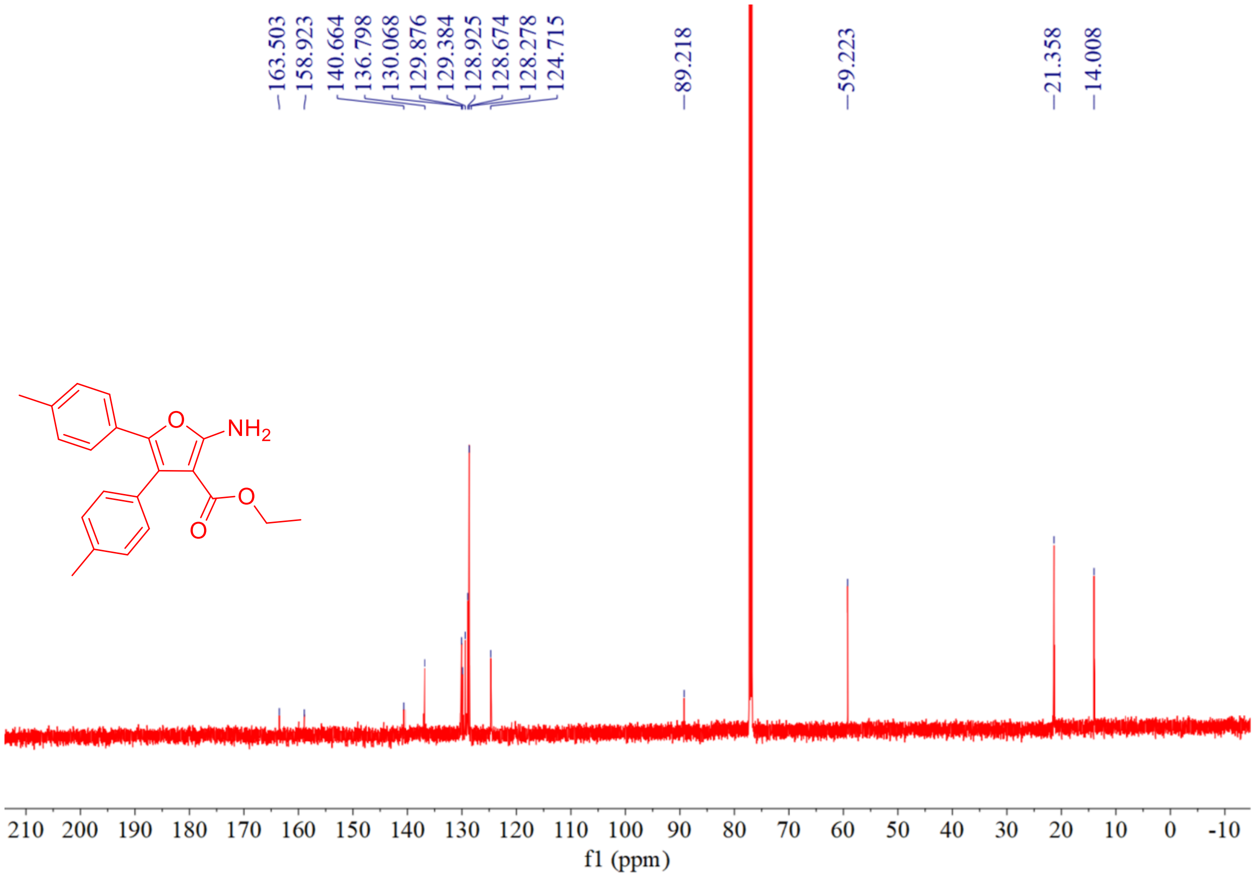

^{13}C NMR spectrum of compound **3w**

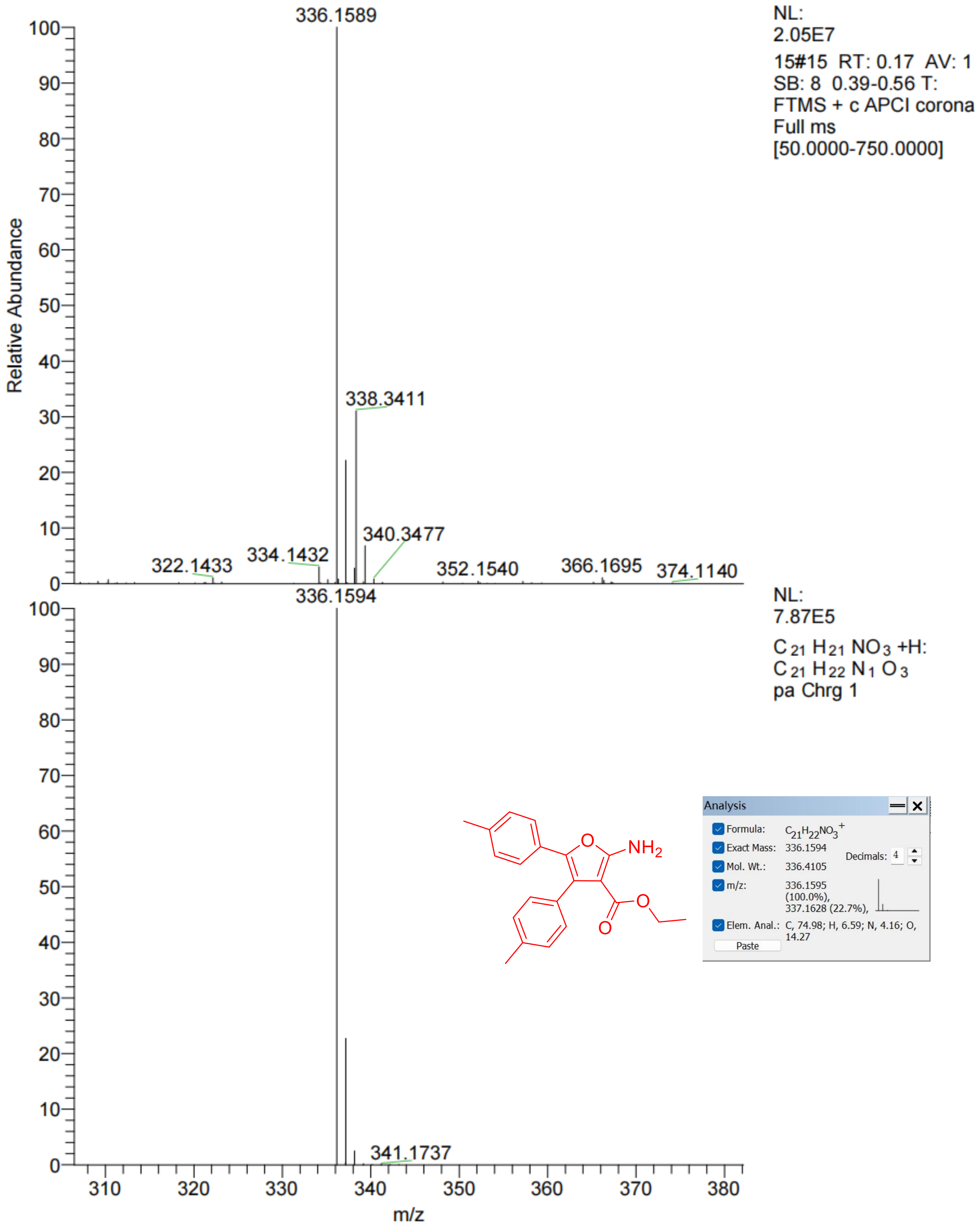

HR-MS spectrum of compound **3w**

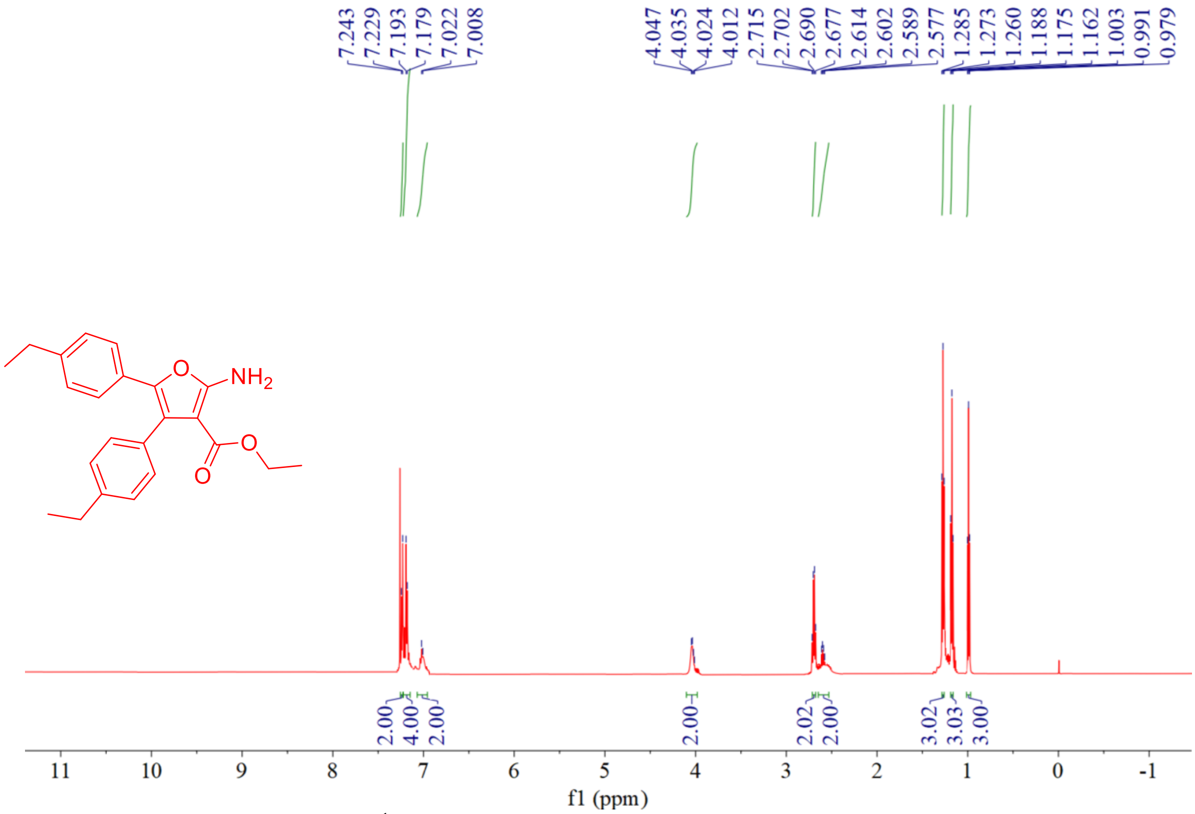

^{1}H NMR spectrum of compound **3x**

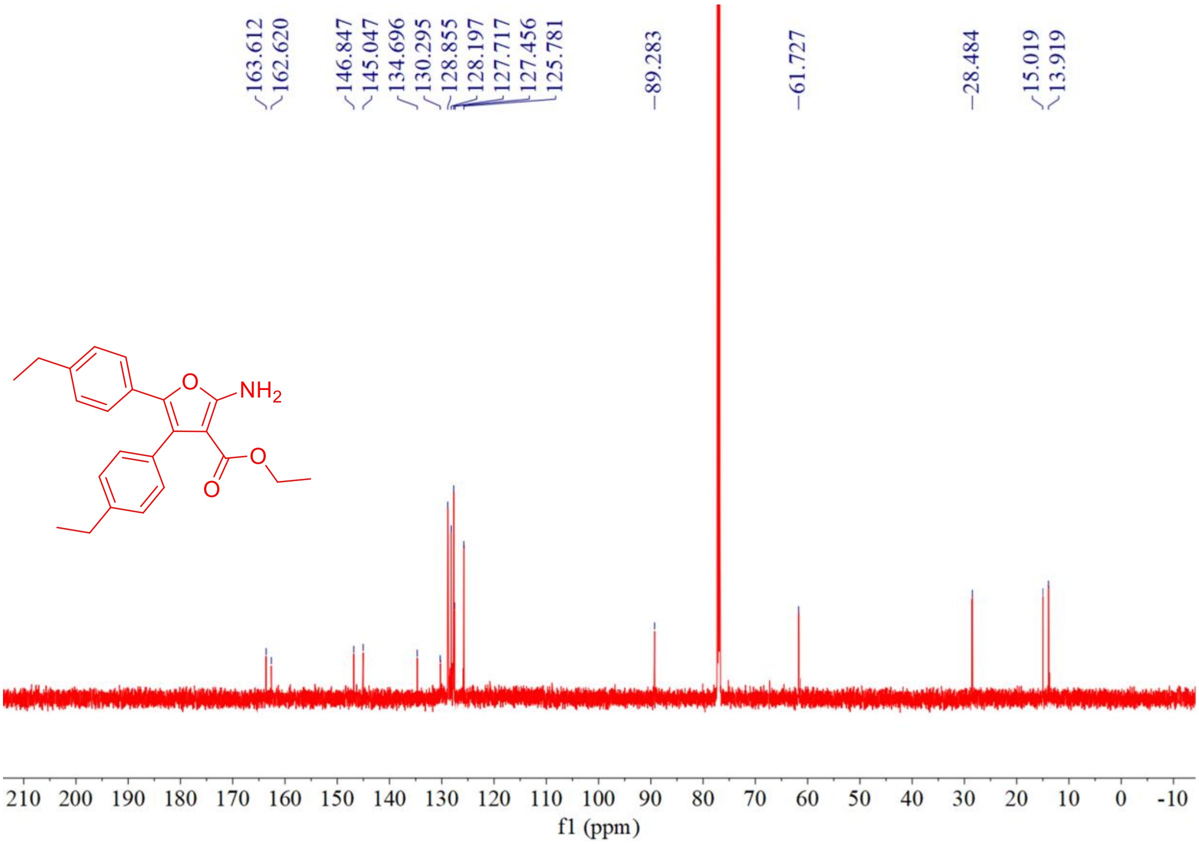

^{13}C NMR spectrum of compound **3x**

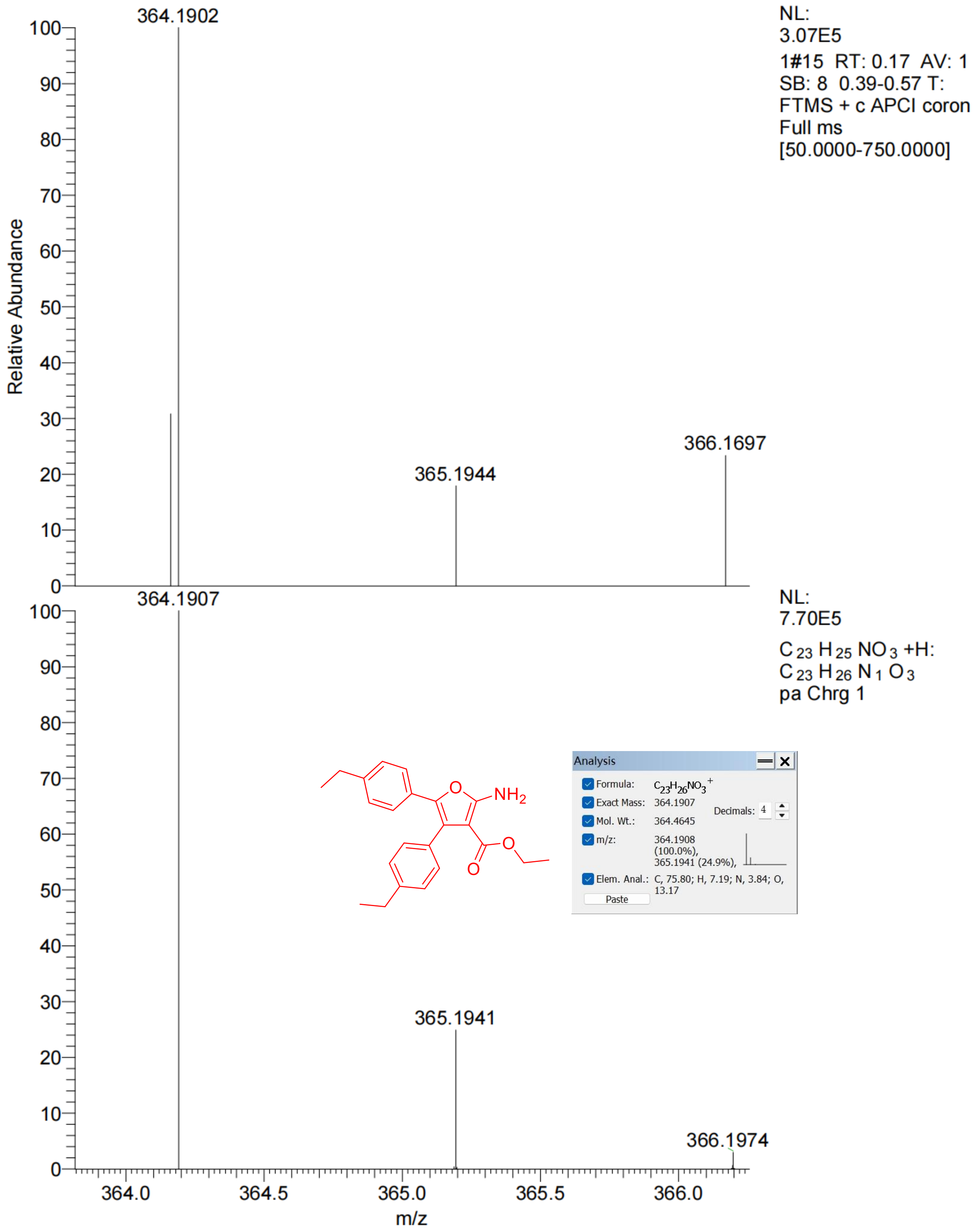

HR-MS spectrum of compound **3x**

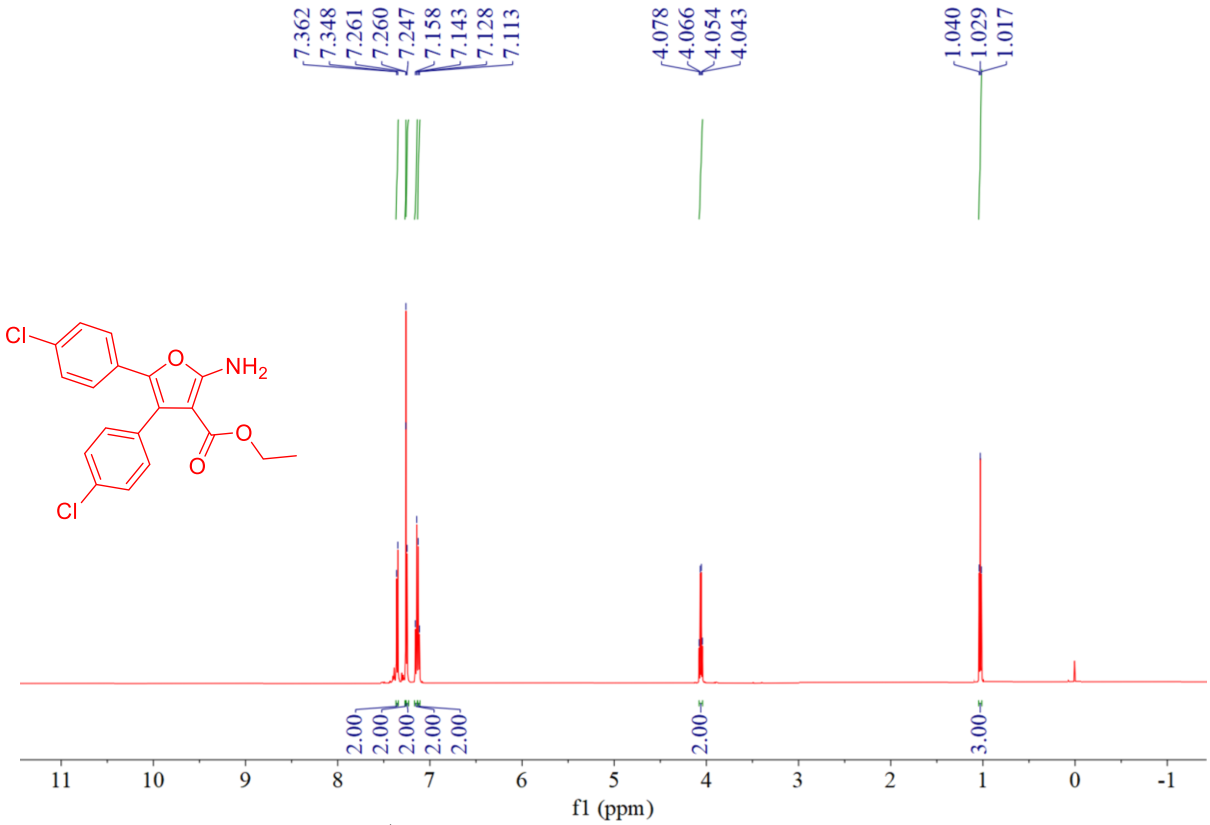

^{1}H NMR spectrum of compound **3y**

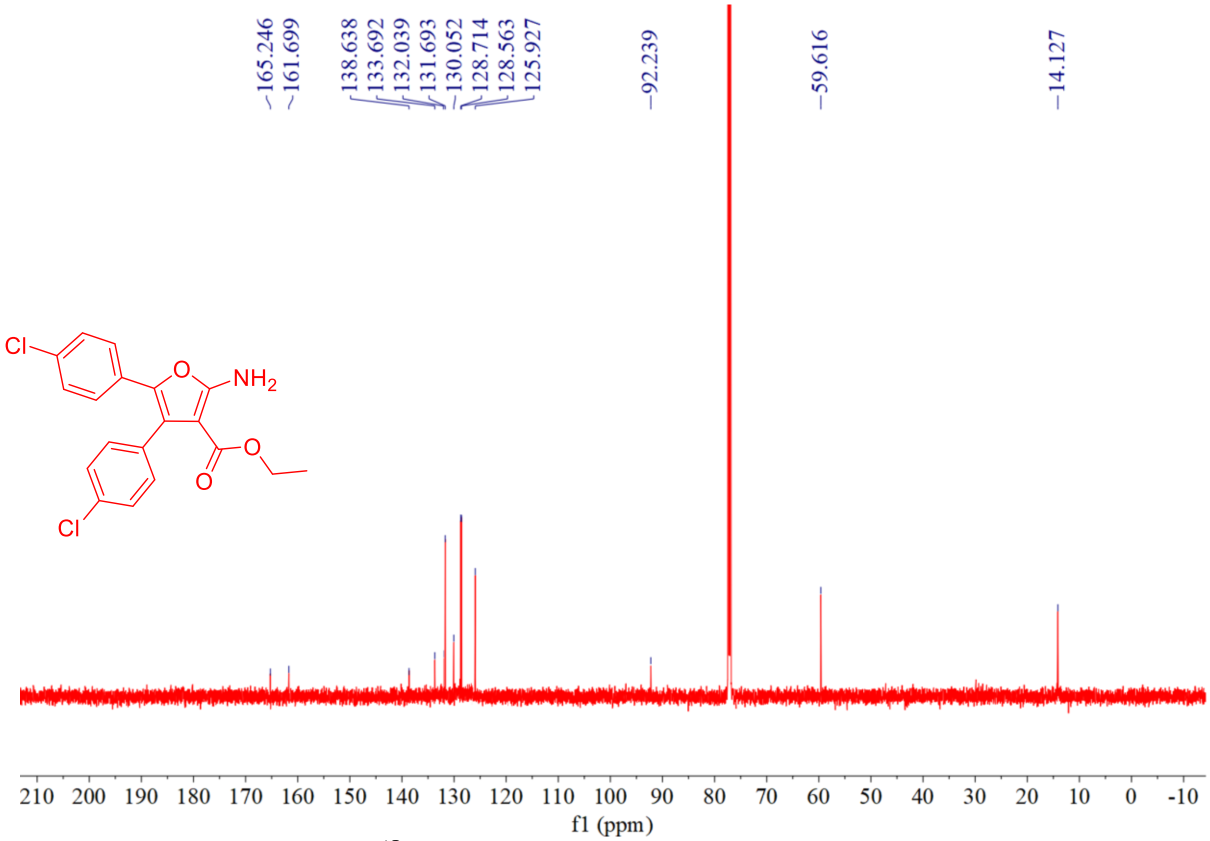

^{13}C NMR spectrum of compound **3y**

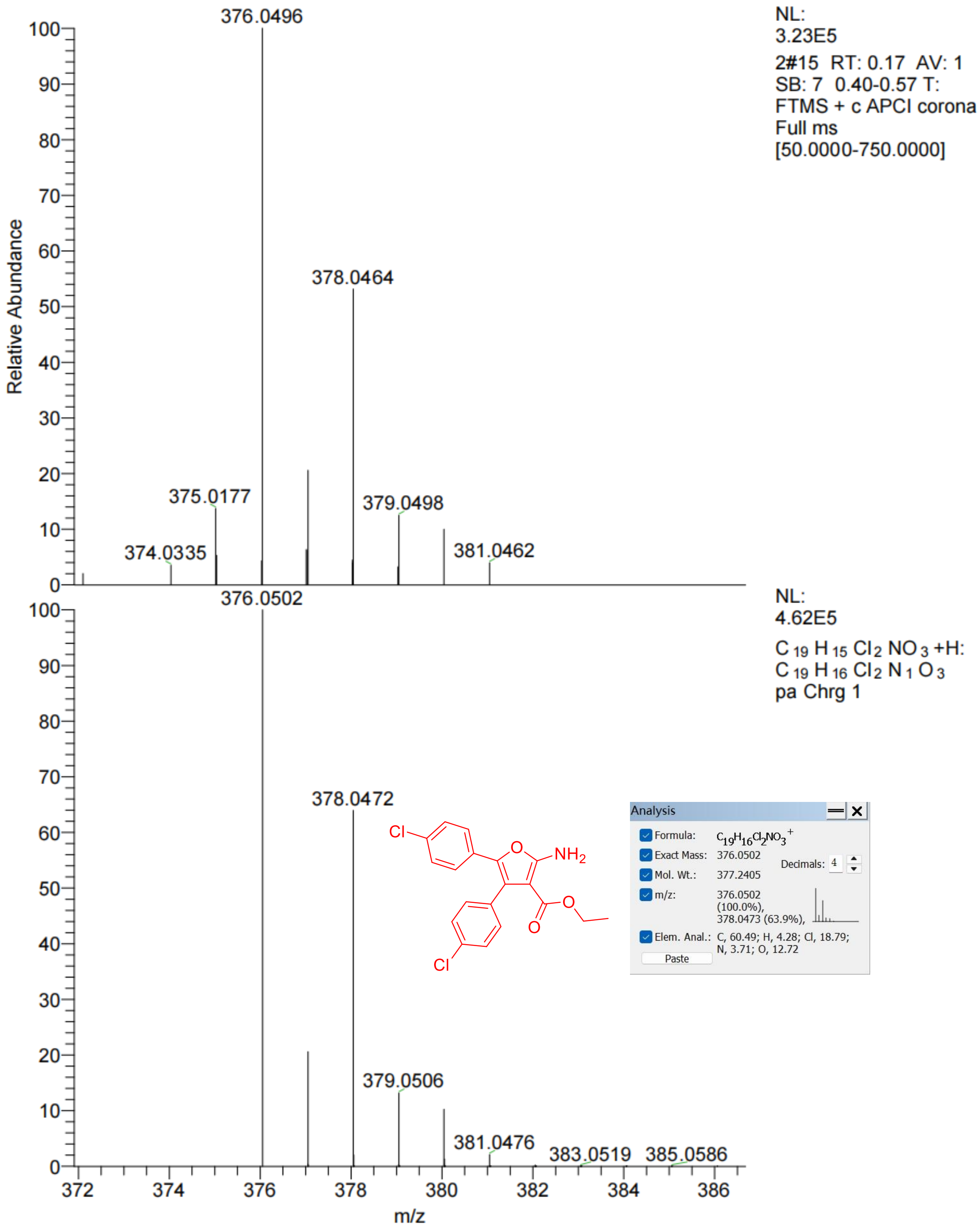

HR-MS spectrum of compound **3y**

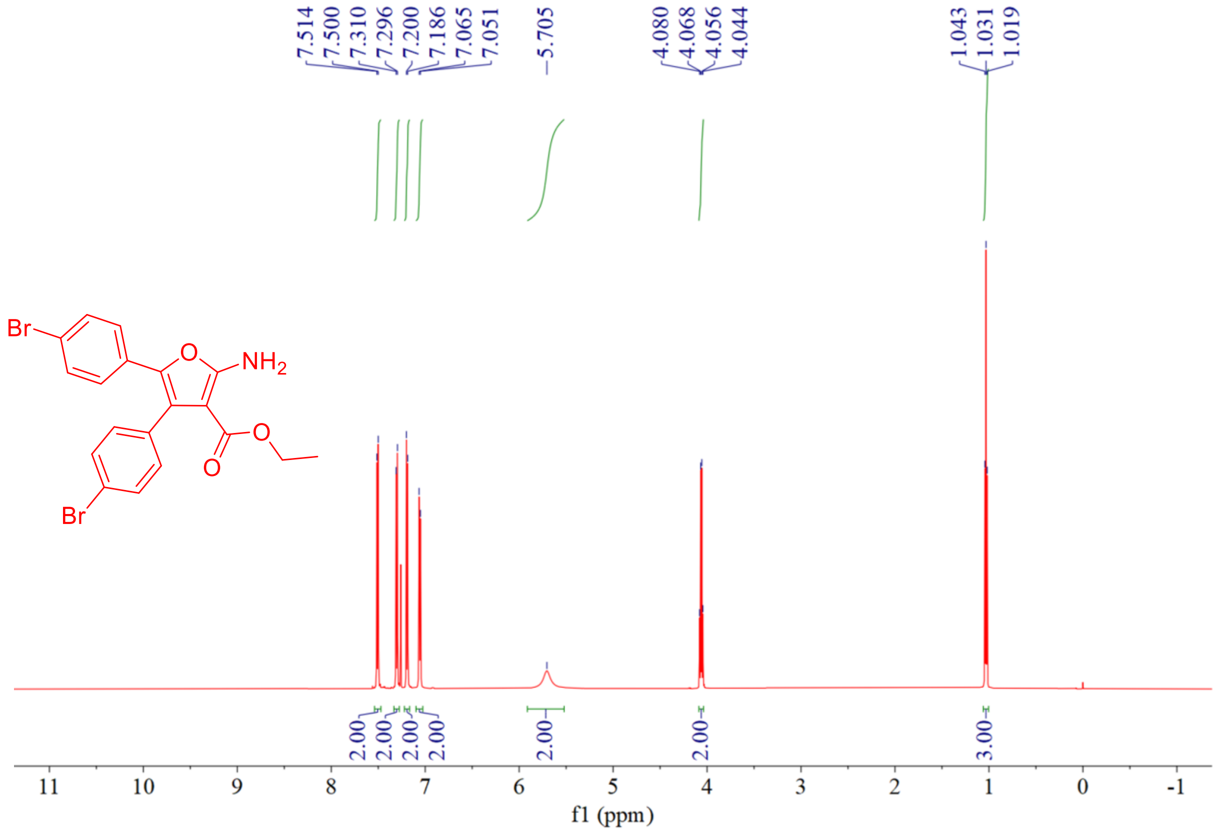

^{1}H NMR spectrum of compound **3z**

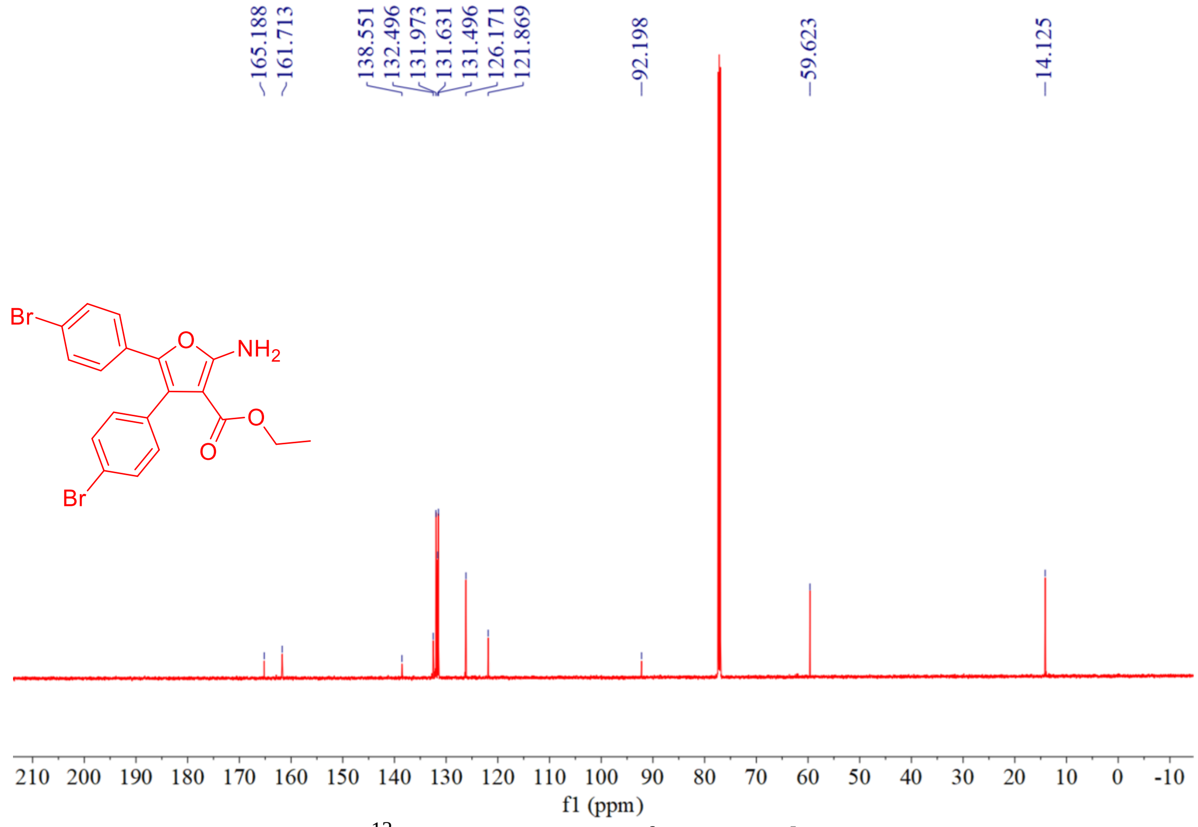

^{13}C NMR spectrum of compound **3z**

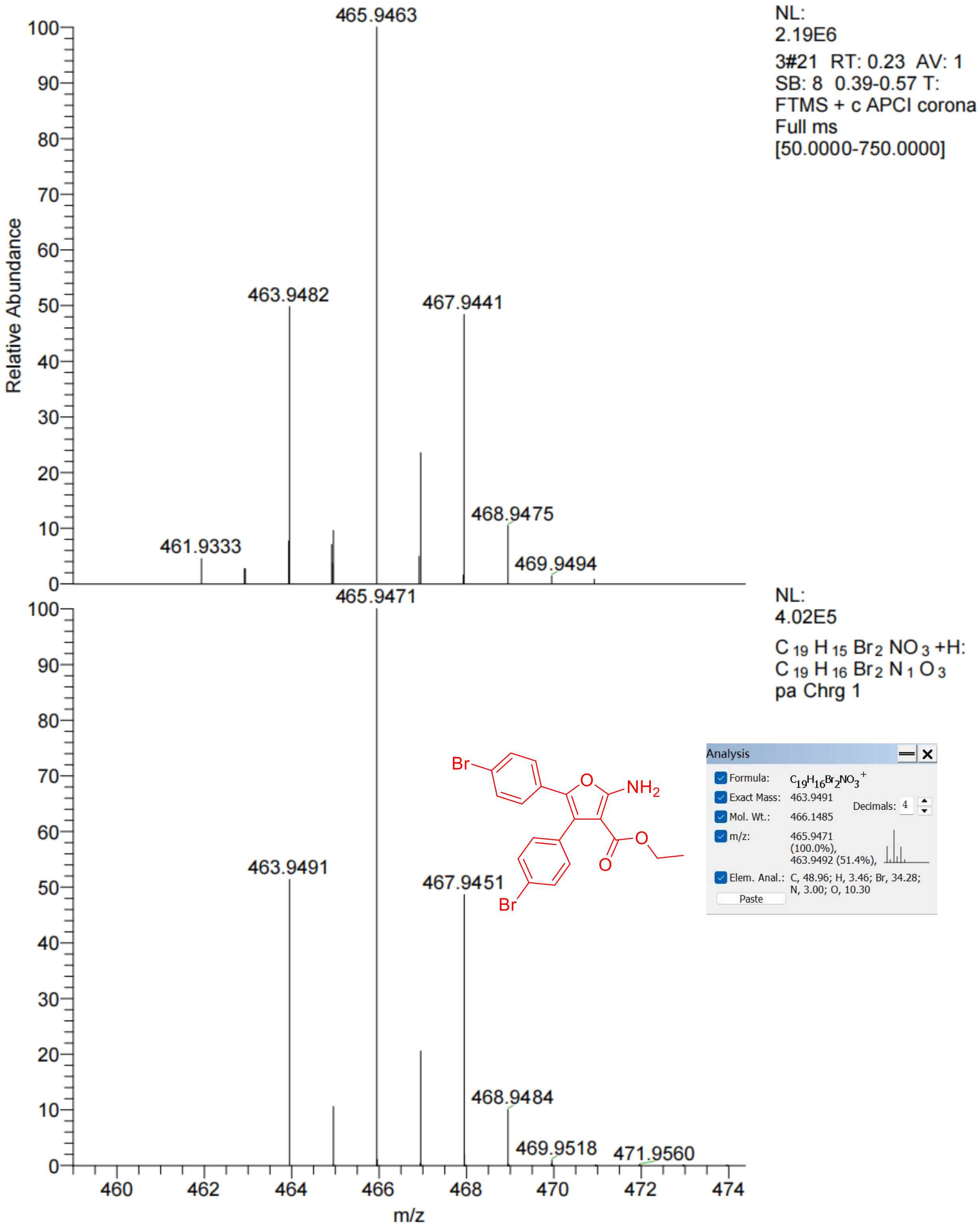

HR-MS spectrum of compound **3z**

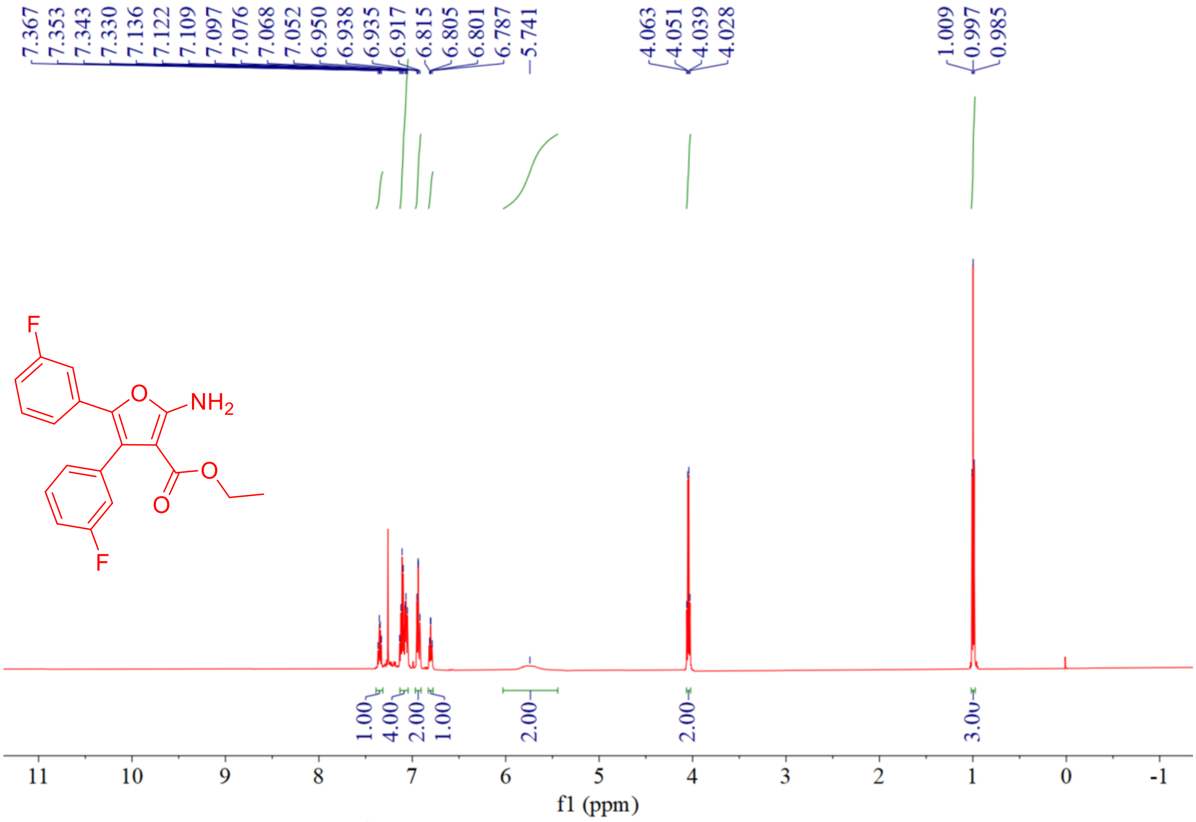

^{1}H NMR spectrum of compound **3aa**

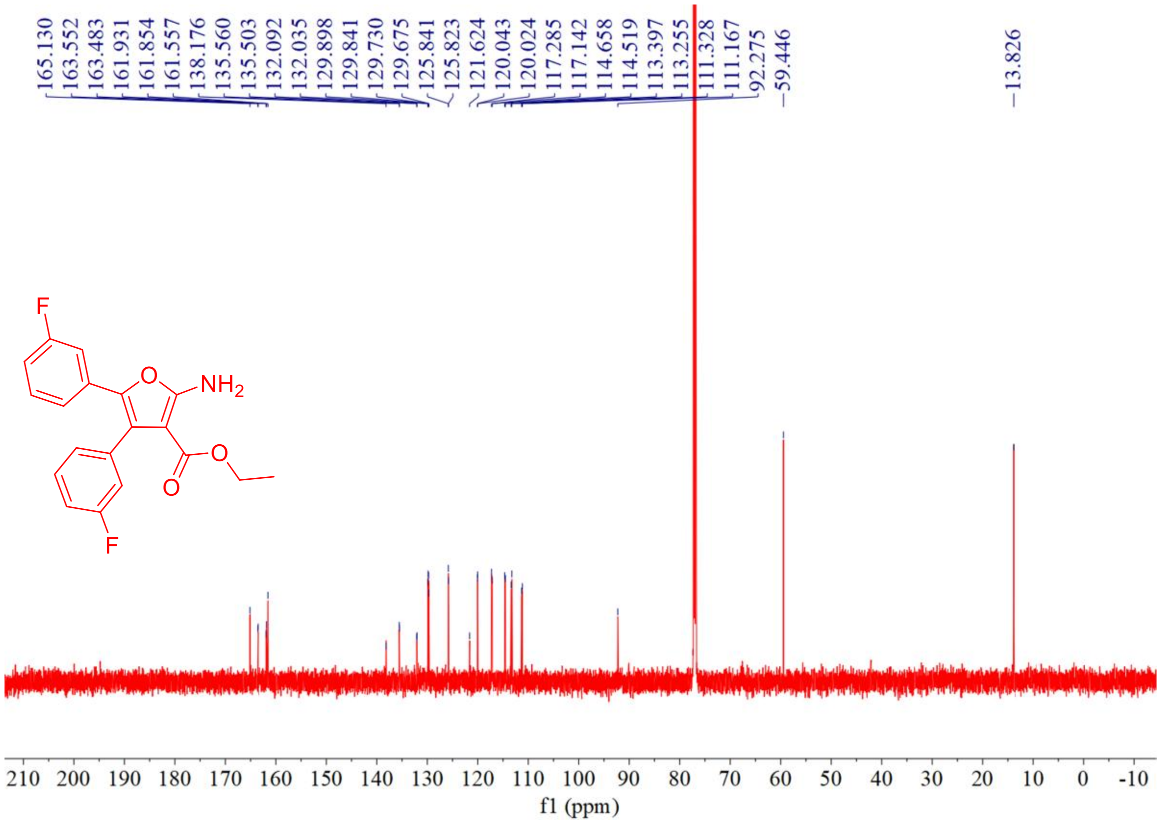

^{13}C NMR spectrum of compound **3aa**

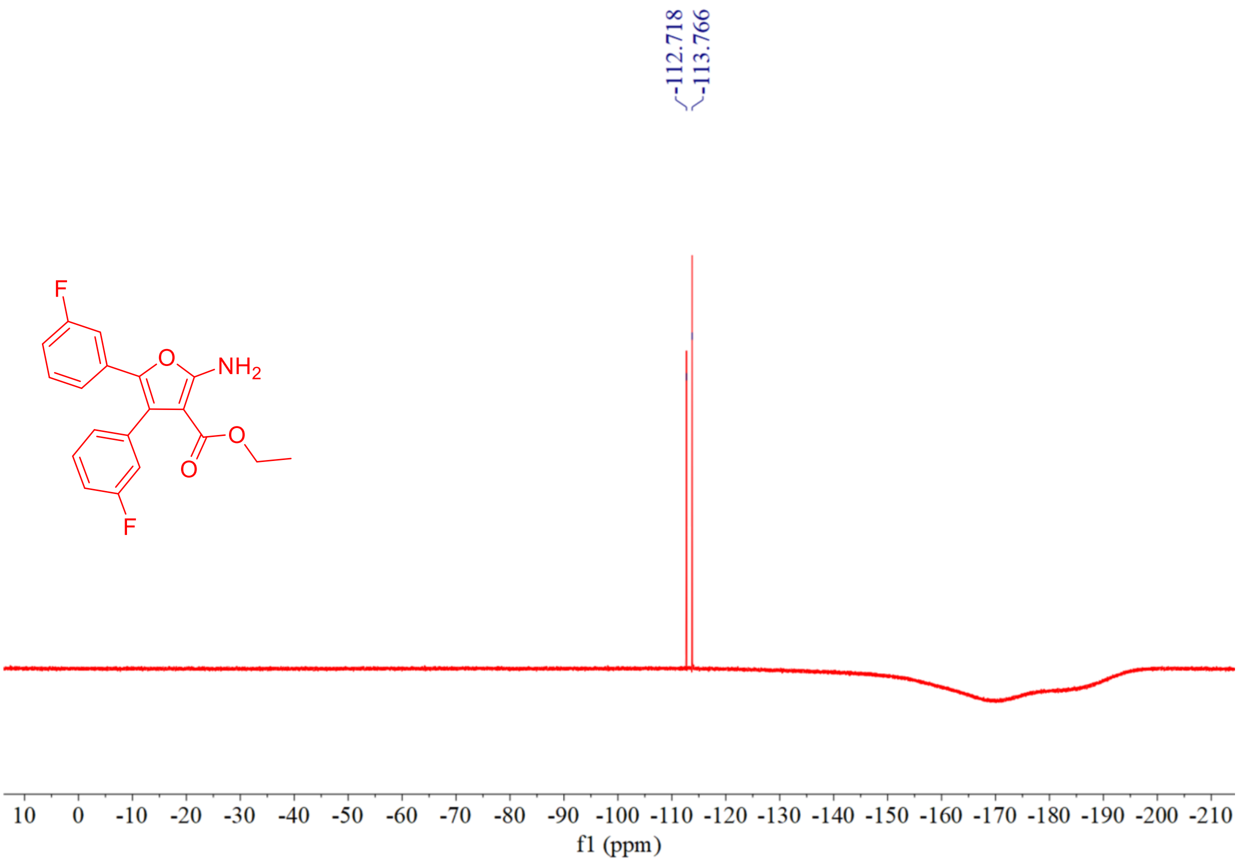

^{19}F NMR spectrum of compound **3aa**

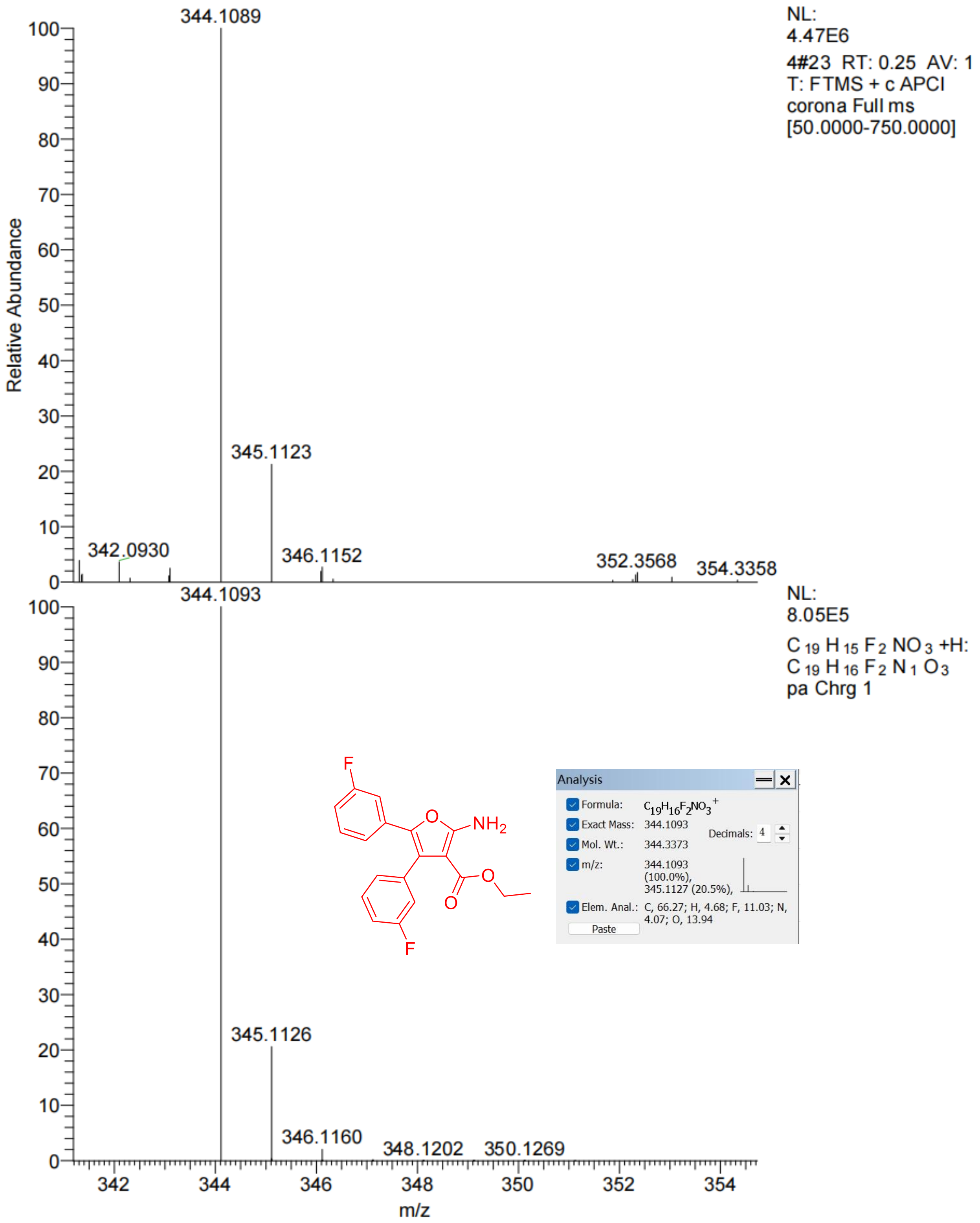

HR-MS spectrum of compound **3aa**

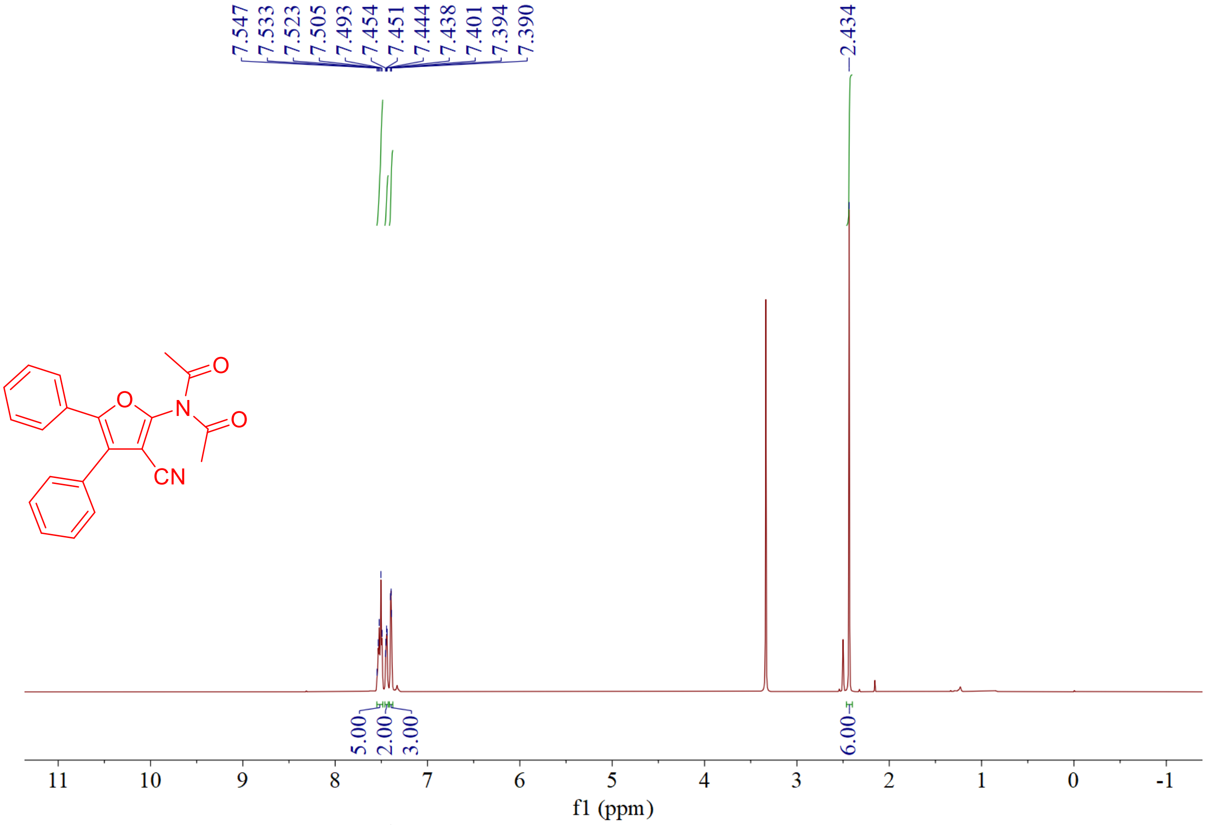

^{1}H NMR spectrum of compound **4a**

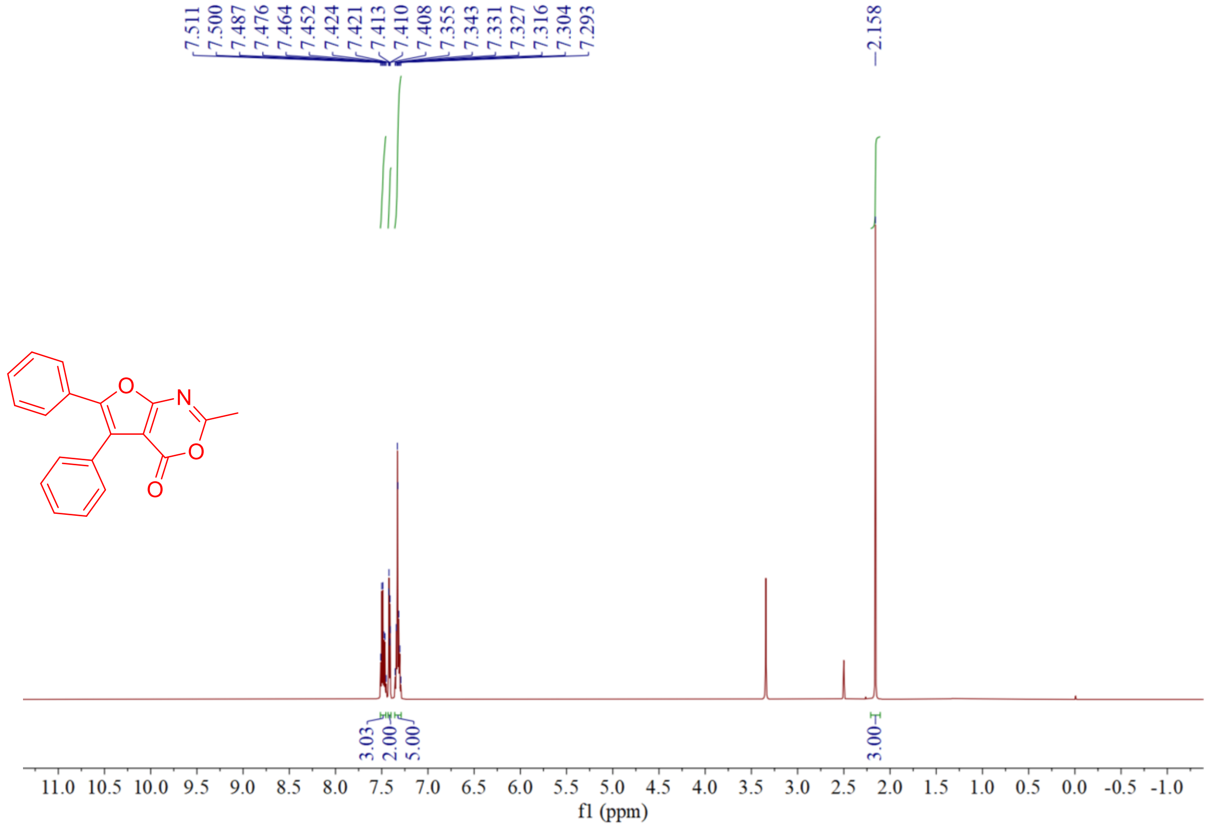

^{1}H NMR spectrum of compound **4b**

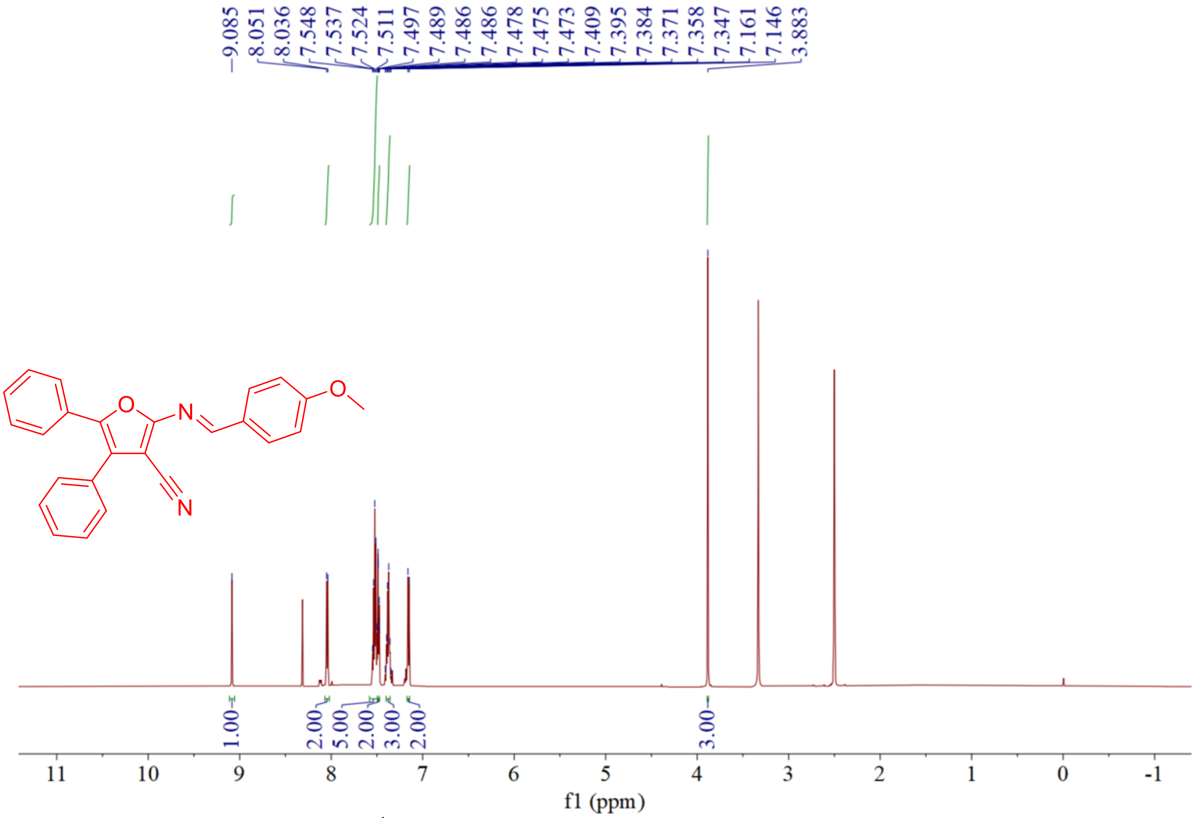

^{1}H NMR spectrum of compound **4c**

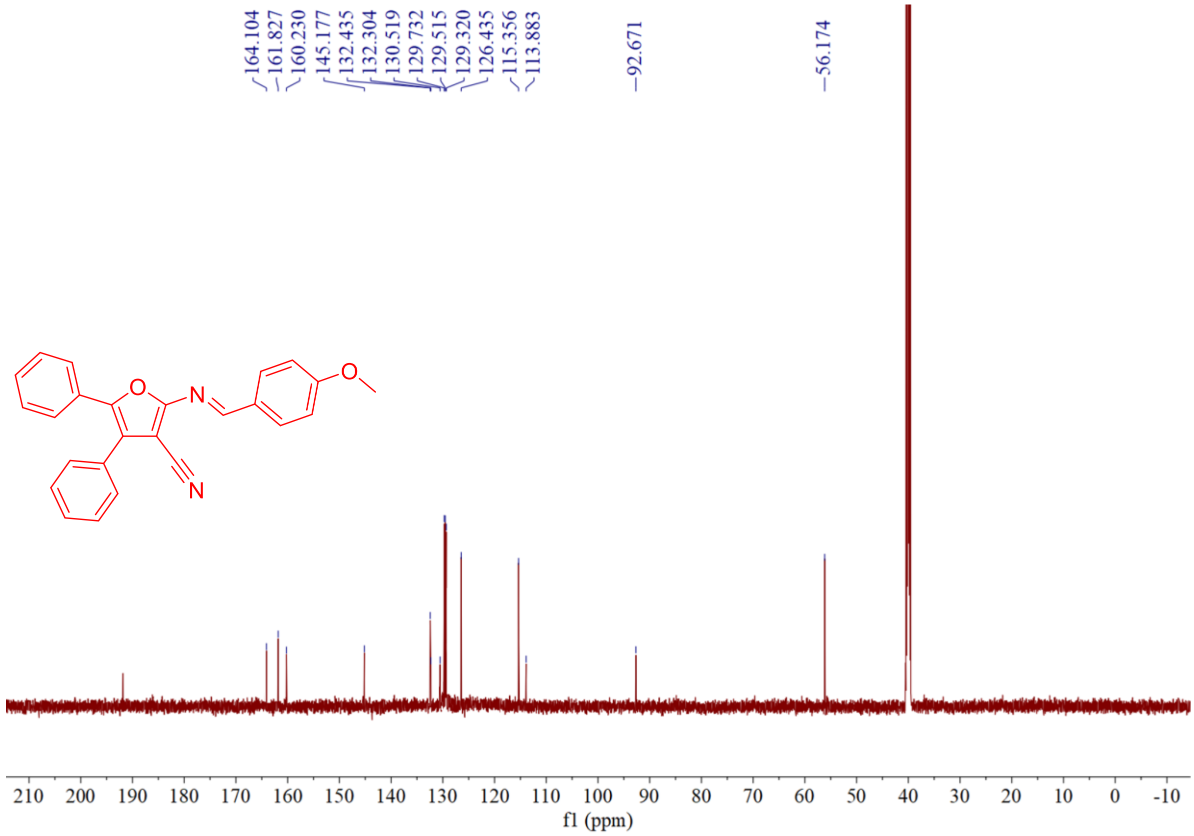

^{13}C NMR spectrum of compound **4c**

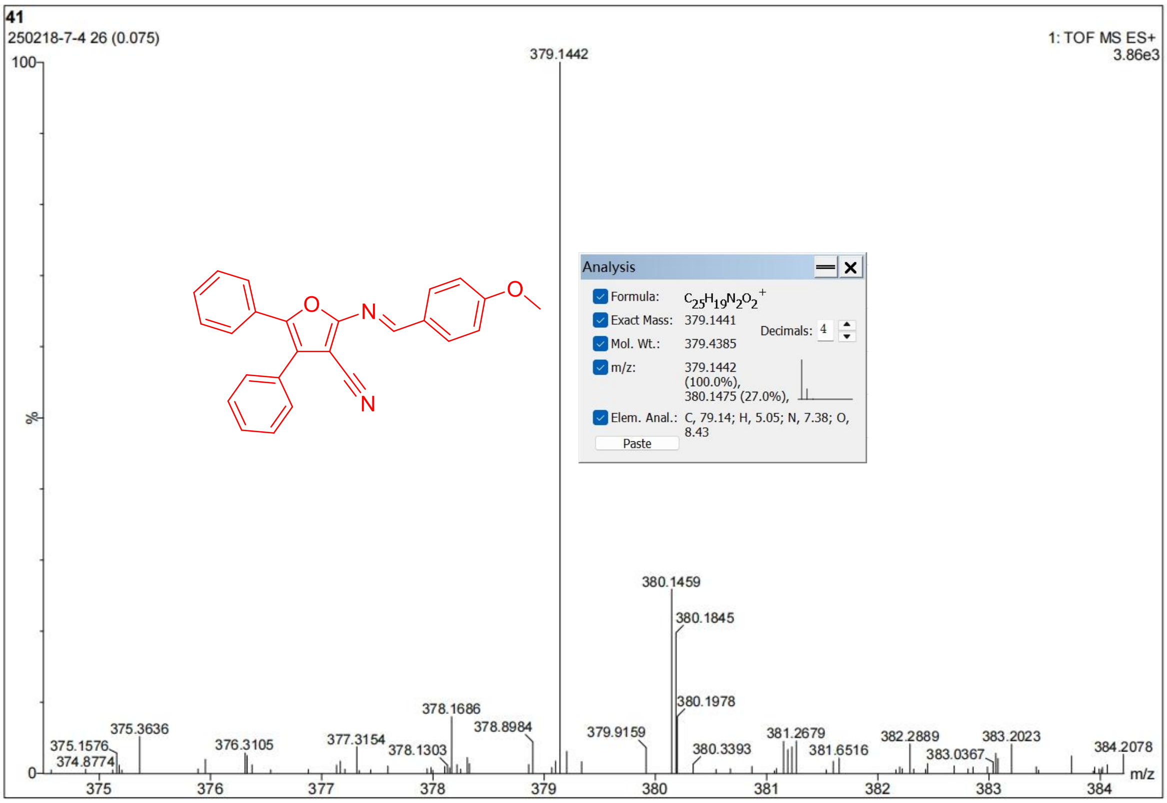

HR-MS spectrum of compound **4c**

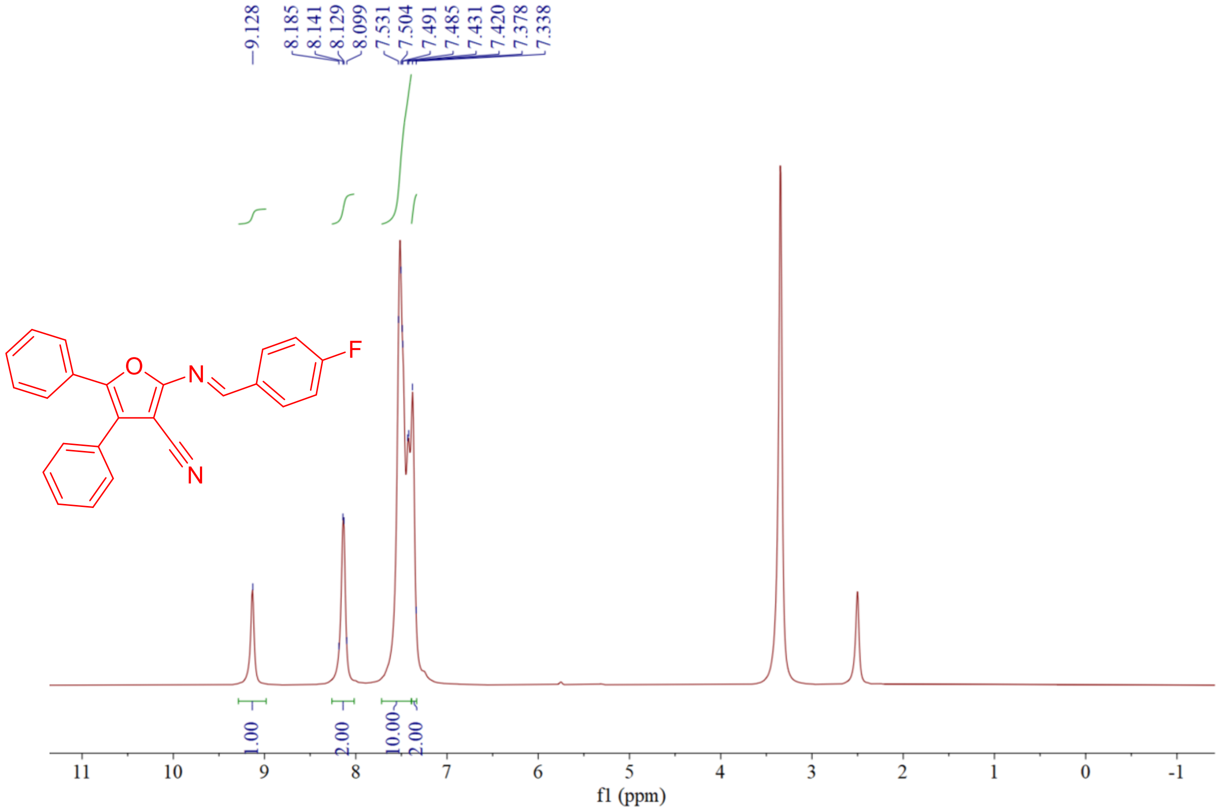

^{1}H NMR spectrum of compound **4d**

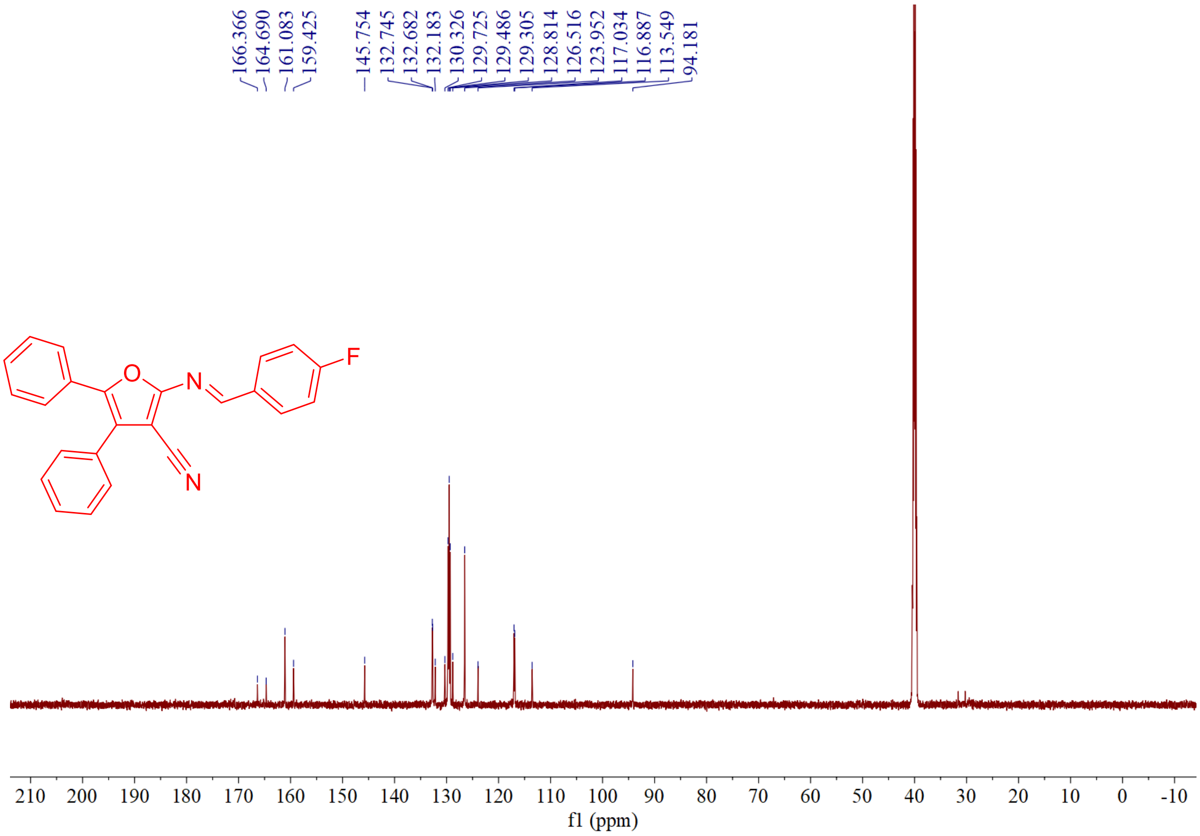

^{13}C NMR spectrum of compound **4d**

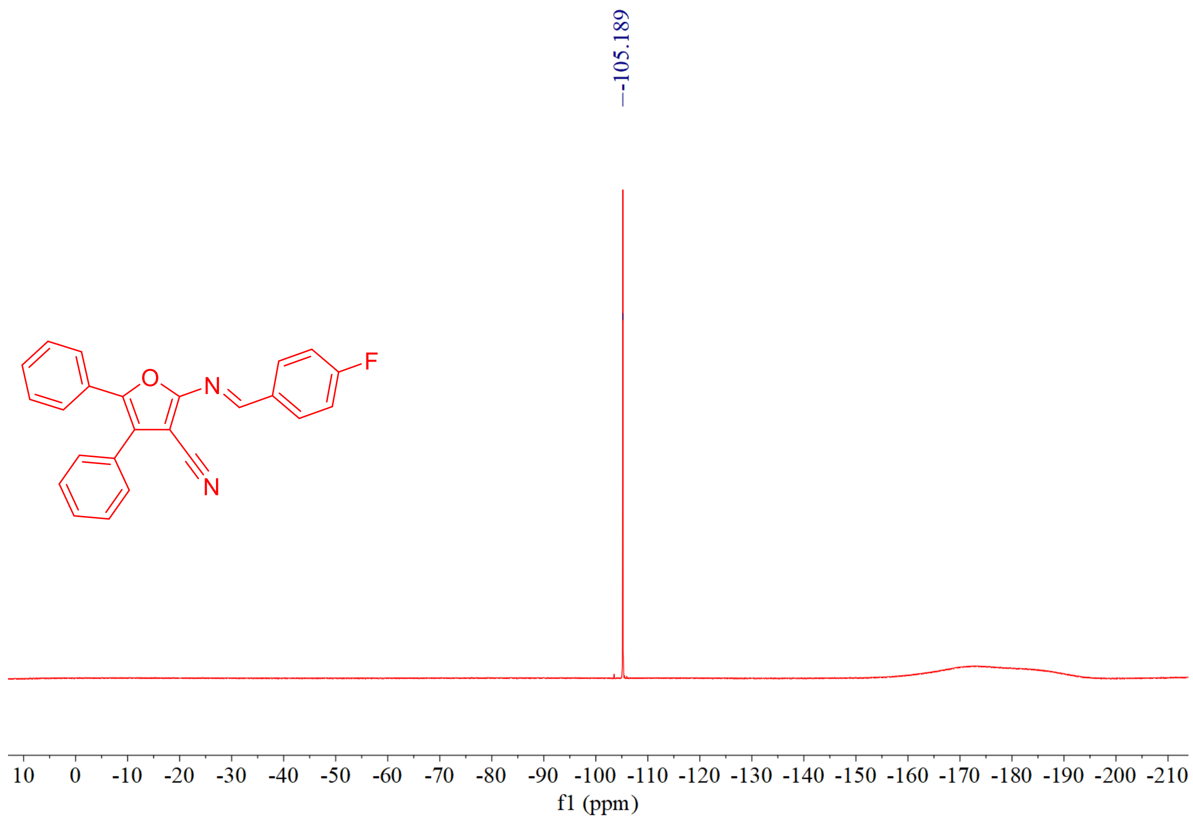

^{19}F NMR spectrum of compound **4d**

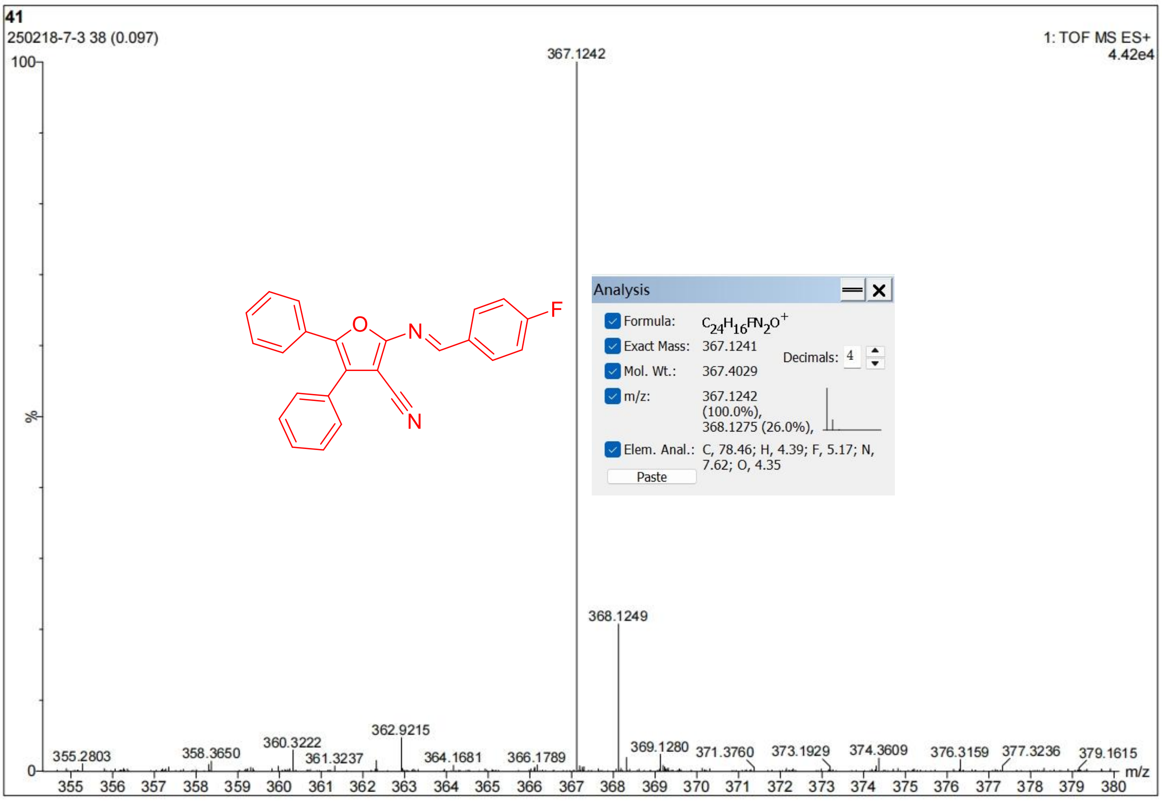

HR-MS spectrum of compound **4d**

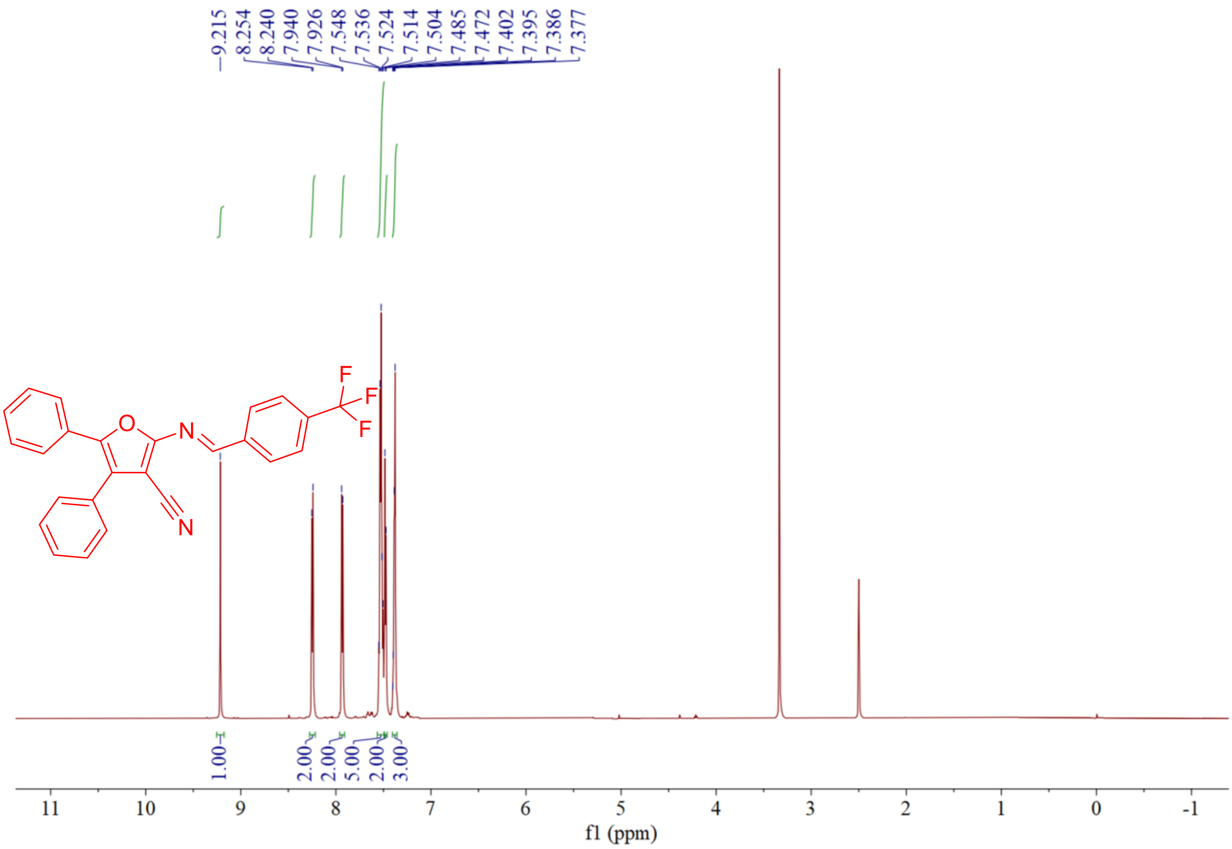

^{1}H NMR spectrum of compound **4e**

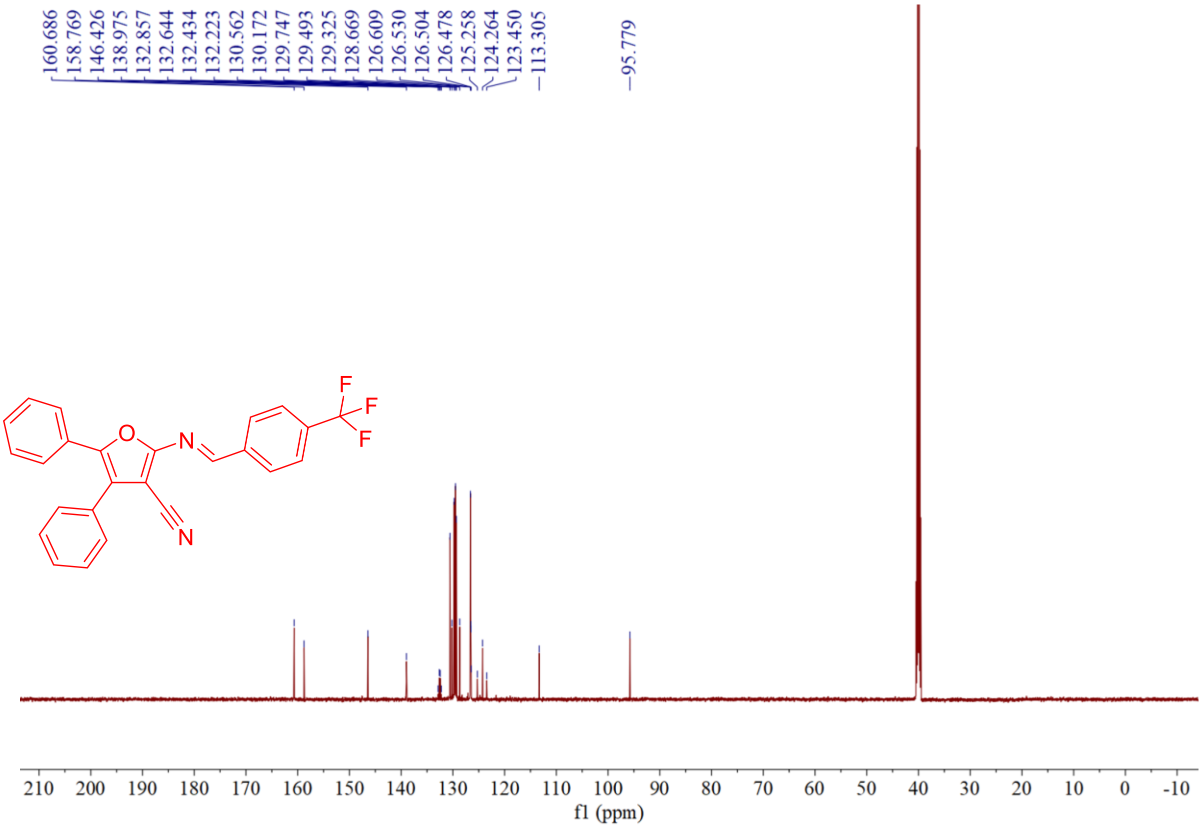

^{13}C NMR spectrum of compound **4e**

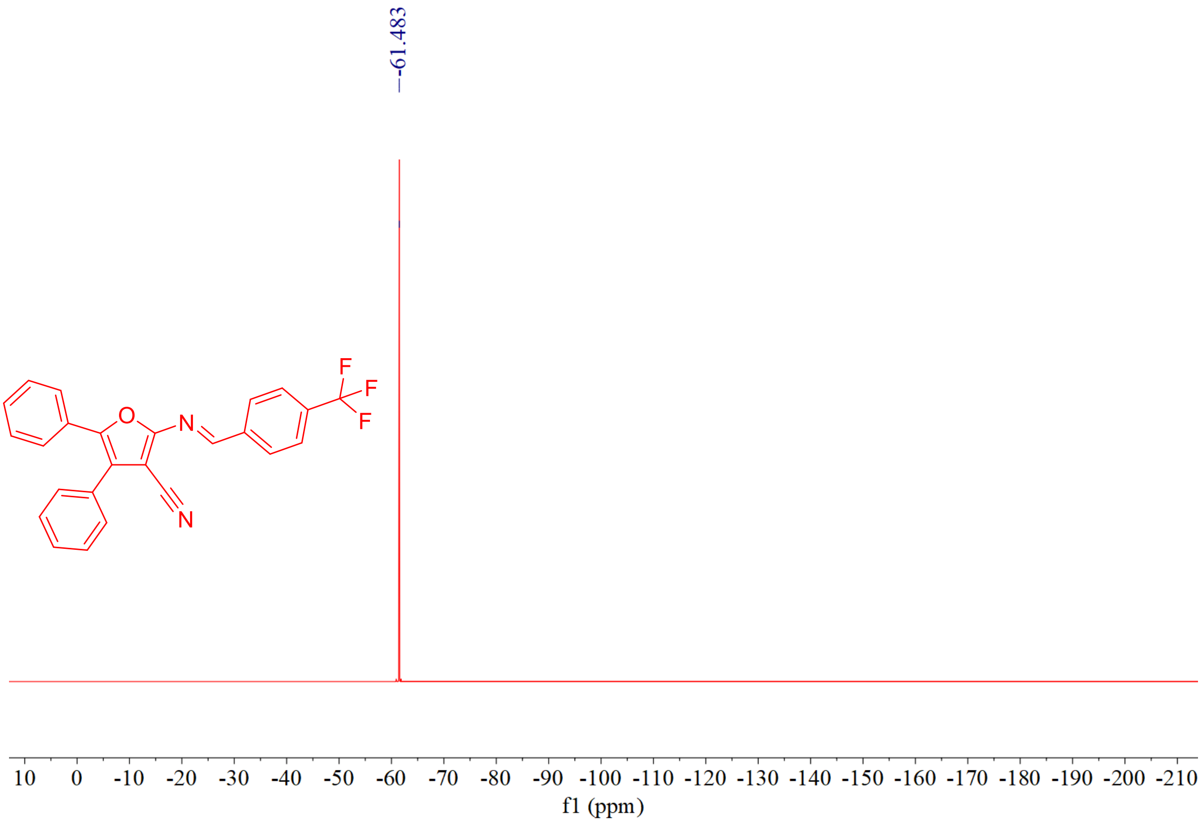

^{19}F NMR spectrum of compound **4e**

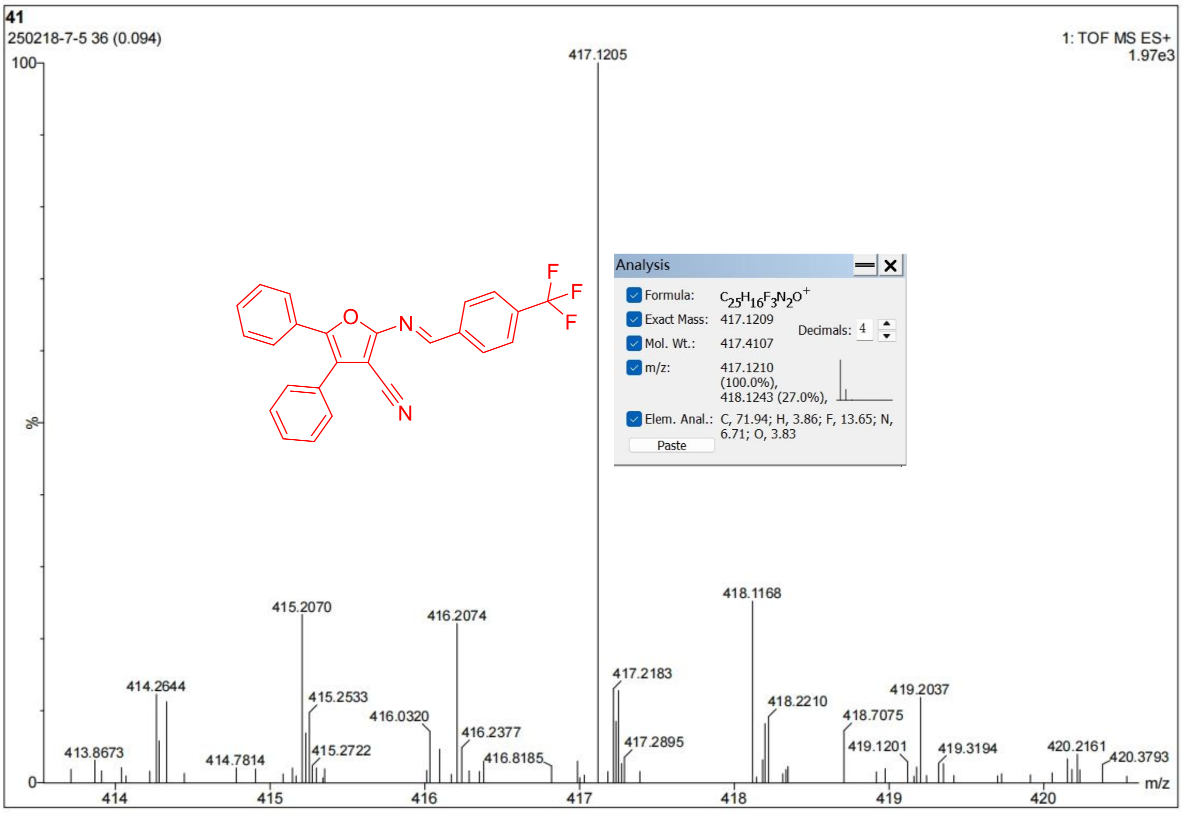

HR-MS spectrum of compound **4e**

References

[1] Yu, C. X.; Lu, J.; Li, T. J.; Wang, D. L.; Qin, B. B.; Zhang, H. H.; Yao, C. S. A NHC-involved, cascade, metal-free, and three-component synthesis of 2,3-diarylated fully substituted furans under solvent-free conditions. *Synlett,* **2011**, *16*, 2420-2424.

[2] Feng, X.; Lancelot, J. C.; Prunier, H.; Rault, S. First synthesis of 4*H*-furo[3,2-*f*]pyrrolo[1, 2-a][1,4]diazepines. *J. Heterocycl. Chem.*, **1996**, *33*(6), 2007-2011.

[3] Prousek, L.; Jurasek, A.; Kovac, L. Reactions and spectral properties of 2-amino-3-cyano-4,5-disubstituted furane derivatives. *Collection Czechoslov. Chem. Commun.*, **1980**, *45*, 1581-1588.

[4] Khan, M. W.; Uddin, M. K.; Ali, M.; Rahman, M. S.; Rashid, M. A.; Chowdhury, R. A convenient synthesis of new annelated pyrimidines and their biological importance. *J. Heterocycl. Chem.*, **2014**, *51*(1), E216-E221.

[5] El-Shahawi, M. M.; El-Ziaty, A. K. Enaminonitrile as building block in heterocyclic synthesis: Synthesis of novel 4*H*-furo[2,3-d][1,3]oxazin-4-one and furo[2,3-d]pyrimidin-4(3*H*)-one derivatives. *J. Chem.*, **2017**, *2017*, 5610707.
